# Proteomic Investigation of *Falciparum* and *Vivax* Malaria for Identification of Surrogate Protein Markers

**DOI:** 10.1371/journal.pone.0041751

**Published:** 2012-08-09

**Authors:** Sandipan Ray, Durairaj Renu, Rajneesh Srivastava, Kishore Gollapalli, Santosh Taur, Tulip Jhaveri, Snigdha Dhali, Srinivasarao Chennareddy, Ankit Potla, Jyoti Bajpai Dikshit, Rapole Srikanth, Nithya Gogtay, Urmila Thatte, Swati Patankar, Sanjeeva Srivastava

**Affiliations:** 1 Wadhwani Research Center for Biosciences and Bioengineering, Department of Biosciences and Bioengineering, Indian Institute of Technology Bombay, Powai, Mumbai, India; 2 Strand Life Sciences Pvt. Ltd., Bangalore, India; 3 Department of Clinical Pharmacology, Seth GS Medical College & KEM Hospital, Parel, Mumbai, India; 4 Proteomics Laboratory, National Centre for Cell Science, Ganeshkhind, Pune, India; 5 Wipro GE Healthcare, Mumbai, India; Kenya Medical Research Institute (KEMRI), Kenya

## Abstract

This study was conducted to analyze alterations in the human serum proteome as a consequence of infection by malaria parasites *Plasmodium falciparum* and *P. vivax* to obtain mechanistic insights about disease pathogenesis, host immune response, and identification of potential protein markers. Serum samples from patients diagnosed with *falciparum* malaria (FM) (n = 20), *vivax* malaria (VM) (n = 17) and healthy controls (HC) (n = 20) were investigated using multiple proteomic techniques and results were validated by employing immunoassay-based approaches. Specificity of the identified malaria related serum markers was evaluated by means of analysis of leptospirosis as a febrile control (FC). Compared to HC, 30 and 31 differentially expressed and statistically significant (*p*<0.05) serum proteins were identified in FM and VM respectively, and almost half (46.2%) of these proteins were commonly modulated due to both of the plasmodial infections. 13 proteins were found to be differentially expressed in FM compared to VM. Functional pathway analysis involving the identified proteins revealed the modulation of different vital physiological pathways, including acute phase response signaling, chemokine and cytokine signaling, complement cascades and blood coagulation in malaria. A panel of identified proteins consists of six candidates; serum amyloid A, hemopexin, apolipoprotein E, haptoglobin, retinol-binding protein and apolipoprotein A-I was used to build statistical sample class prediction models. By employing PLS-DA and other classification methods the clinical phenotypic classes (FM, VM, FC and HC) were predicted with over 95% prediction accuracy. Individual performance of three classifier proteins; haptoglobin, apolipoprotein A-I and retinol-binding protein in diagnosis of malaria was analyzed using receiver operating characteristic (ROC) curves. The discrimination of FM, VM, FC and HC groups on the basis of differentially expressed serum proteins demonstrates the potential of this analytical approach for the detection of malaria as well as other human diseases.

## Introduction

The burden of malaria continues to worsen globally with a devastating impact on human health and corresponding impediment to economic improvement [1]. Despite worldwide initiatives, emerging drug resistance in different species of Plasmodium and paucity of information about the exact underlying mechanism of the disease pathogenesis are creating challenges for the management and eradication of the disease. *Plasmodium falciparum (Pf)* infection represents the major cause of malaria associated morbidity and mortality worldwide. *Falciparum* malaria (FM) accounts for approximately 247 million cases and one million deaths annually, particularly in sub-Saharan Africa [2], while outside the African continents, *Plasmodium vivax (Pv)* is responsible for more than 50% of all malaria cases [3]. In order to survive within the host cells and ensure their reproduction, intracellular parasites like Plasmodium develop versatile mechanisms to exploit their host cells and induce new permeability pathways to permit the uptake of nutrients and the removal of waste products, resulting into activation of multiple host immune cascades and inflammatory responses [4]. Plasmodium infection also affects blood coagulation by diverse pathobiological mechanisms, which results into development of fatal hemorrhagic complication [5], [6]. Investigation of the parasite induced alterations in host proteome and modulation of different vital physiological processes have great clinical relevance in the light of diagnosis and prognosis. Recently, proteomic studies have contributed substantially to our understanding of the clinical proteome of human malaria parasites [7], profiling humoral immune responses to Plasmodium infection [8] and the malaria parasite infection-induced changes in host erythrocyte membrane proteins [9]. The findings obtained from such studies have provided better understanding of the disease pathogenesis, host-pathogen interactions and host immune response.

Analysis of human serum proteome is found to be very useful for the identification of potential disease-related markers, understanding disease pathogenesis and host immune response since various serum proteins exhibit rapid alteration in expression pattern in response to diseased conditions and show direct correlation with disease progression [10]. In recent years, a number of proteomic studies have been carried out to investigate the pathogen induced alterations in human serum/plasma proteome in different infectious diseases including dengue [11], SARS [12], leishmaniasis [13], and leptospirosis [14]. In this study we have investigated the alterations in human serum proteome due to the *P. falciparum* infection for obtaining mechanistic insight about the disease pathogenesis and host immune response in the most virulent form of human malaria. Additionally, serum proteome changes in FM were compared with *vivax* malaria (VM); another widely distributed human malaria to study the similarities and differences in host responses against these two major plasmodial infections. To achieve this comparative analysis we have utilized selected dataset of our previous serum proteomics study on VM [15], while additional proteomics and immune-assay-based experiments were performed using a bigger (compared to our previous report) clinical cohort of VM patients. The comparative study on FM and VM revealed that quite a few serum proteins associated with diverse essential physiological pathways, including acute phase response signaling, cytokine and chemokine signaling, complement cascades and blood coagulation are commonly altered in both of the plasmodial infections, while some uniquely modulated candidates such as calcium binding protein 39, calpain 10, regulator of G-protein signaling 7, serum paraoxonase/arylesterase, transthyretin in FM and ceruloplasmin, vitamin D-binding protein, serum amyloid P, alpha-2-macroglobulin, fibrinogen beta chain precursor in VM, were also identified. Recently, we performed serum proteomic alterations in another clinically relevant infectious disease, leptospirosis [14]. To evaluate the specificity of the identified protein targets and eliminate the generic febrile responses; expression level of the serum proteins differentially expressed in plasmodial infections (compared to the healthy subjects) was analyzed in leptospirosis patients from our previous study [14]. Another major intention of this present study was identification of the characteristics marker proteins, which can readily discriminate malaria patients (FM from VM as well) from healthy population as well as closely related infectious diseases with high accuracy. The potential serum protein biomarkers identified in our study were used to build statistical models, which successfully classified and predicted the clinical phenotypes of controls (healthy and febrile), FM and VM in a blinded study.

## Results

### Study Population Profiles

Stringent inclusion criteria were employed during the selection of malaria patients and controls (HC and FC) to reduce pre-analytical variations. Malaria patients (FM and VM) selected for this proteomic analysis were suffering from uncomplicated, non-severe plasmodial infections with comparable range of parasitemia. Blood samples were collected from the malaria patients before administration of any antimalarial drugs. Majority of the patients were suffering their first episode of malaria, while some of the subjects had a past history of this disease (relapse or recurrent). The average age of the FM and VM patients included in this proteomic analysis was 34.2 years (SD = 10.93; range 20–53; median 35) and 32.9 years (SD = 10.76; range 20–52; median 32), respectively (Table 1). To maintain uniform population profiles of test (FM and VM) and controls (HC and FC) for differential protein expression analysis, healthy and febrile control (leptospirosis patients) populations with comparable age distribution; mean values 33.4 years (SD = 8.69; range 20–44; median 31.6) and 30.5 years (SD = 8.31; range 23–42; median 26.5), respectively for HC and FC, were selected (Table 1).

**Table 1:** Demographics and clinical details of the malaria patients and febrile controls (Leptospirosis patients).

Disease	*Falciparum* malaria* [n = 20]	*Vivax* malaria* [n = 17]	Febrile control(Leptospirosis) [n = 6]
**Age (Year) #**	35 (20–53)	32 (20–52)	26.5 (23–42)
**Parasitemia (No./µL blood) #**	2300 (1160–35160)	3404 (1600–9028)	–
**Hematological parameters #**			
Hb (g/dL)	10.95 (6.2–13.2)	11.9 (9–16.3)	12.1 (8.5–14.4)
ESR (mm/hr)	50.5 (6–170)	35 (7–110)	74.5 (64–78)
RBC/µL (Millions)	4.12 (3.38–5.64)	4.77 (3.82–6.1)	NA^•^
Platelets/µL (Thousands)	116 (14–440)	160 (45–310)	109 (36–290)
**Liver function tests #**			
Total bilirubin (mg %)	1.35 (0.45–4.01)	0.96 (0.50–2.71)	1.13 (0.68–1.67)
SGOT (IU/L)	34.05 (17.68–152)	32.5 (9.6–101.9)	60.11(26.8–145.64)
SGPT (IU/L)	24.2 (11.7–76.02)	33.49 (17.68–106.1)	58.34 (23.89–108.7)
Alkaline phosphatase (IU/L)	78.32 (48.29–190.6)	72.41 (52.09–126.6)	86.54 (46.9–167.7)
**Type of infection**	Uncomplicated, non-severe Mostly 1^st^episode; few are recurrent cases	Uncomplicated, non-severe Mostly 1^st^episode; few are recurrent/relapse cases	Non-severe infection;1^st^ episode

# Data is represented as median (interquartile-range).

* Not suffering from mix infections [multiple *Plasmodium* species] and not treated with any antimalarial drugs prior to the sample collection process.

•Not available.

### Identification of Differentially Expressed Proteins in *Falciparum* and *Vivax* Malaria

In this proteomics study we have performed two levels of gel-based proteomic analysis using classical two-dimensional gel electrophoresis (2DE) and latter more advanced 2D-difference gel electrophoresis (2D-DIGE) technology using sub-sets of the patient and control populations studied by 2DE. All of the 63 samples (20 FM, 17 VM, 6 FC and 20 HC) were analyzed individually by classical 2DE in two technical replicates. While, a sub-set of 30 samples (8 each for FM, VM, HC, and 6 FC) were analyzed by 2D-DIGE. During selection of the sub-set cohorts for 2D-DIGE analysis age range, median age value, sex, level of parasitemia, and past history of systemic disease were considered. Particularly, *falciparum* and *vivax* malaria patients with similar levels of parasitemia (2000–5000 infected RBCs/µL blood) with first episode of infection and no past history of systemic disease were selected for 2D-DIGE analysis to reduce pre-analytical variations as much as possible. In classical 2DE analysis, patients suffering their first episode of malaria as well as few patients with a past history of malaria (relapse or recurrent) and higher level of parasitemia (>5000 infected RBCs/µL blood) were included since it was difficult to get bigger cohort of malaria patients with similar parameters. In gel-based proteomic analysis samples were studied individually (n = 63 for classical 2DE and n = 30 for 2D-DIGE) rather than sample pooling to achieve better insights about biological variability from individual samples.

In proteomic analysis two major high-abundance serum proteins; albumin and IgG were removed using Albumin & IgG Depletion SpinTrap (GE Healthcare) to reduce the dynamic range of the serum proteome. Depletion of these top two high-abundance proteins removes more than 60% of the total protein content in human plasma or serum allowing detection of more proteins by increasing the effective concentration of the low-abundance proteins. Depletion of albumin and IgG effectively increased the overall spot number in 2D gels (Figure S1A). The efficiency of albumin and IgG depletion from human serum was evaluated by densitometric analysis of SDS-PAGE gels containing resolved serum proteins before and after depletion (Figure S1B). The densitometric analysis revealed around 85% and 80% depletion of albumin and IgG, respectively (Figure S1C).

**Figure 1:**
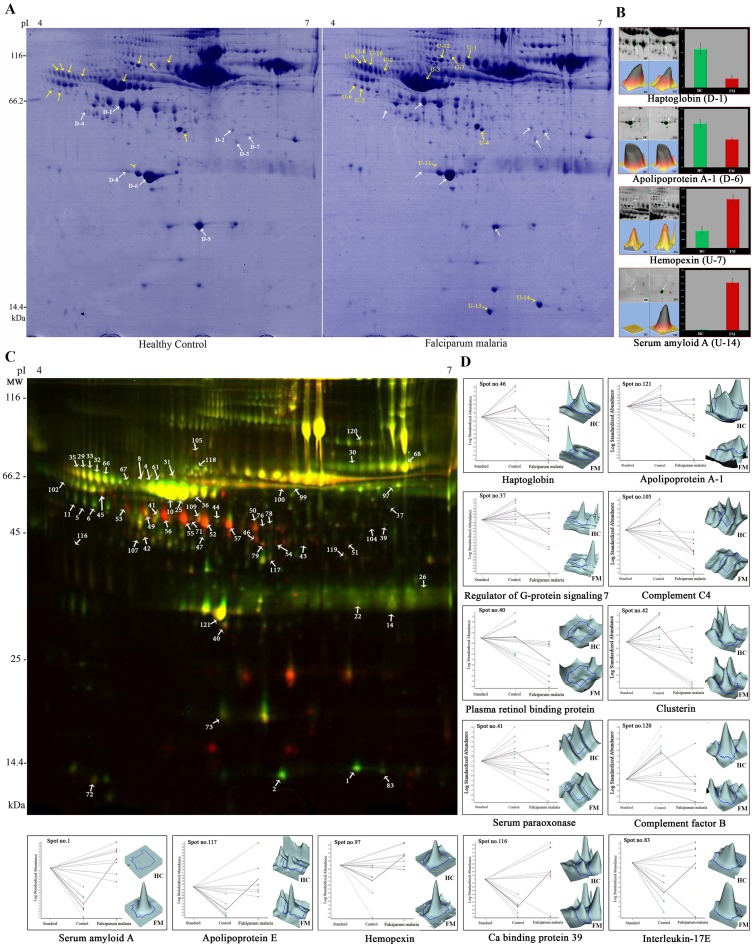
Differentially expressed serum proteins in *falciparum* malaria identified using 2DE and 2D-DIGE. (A) Representative 2D gels of serum from healthy controls and FM patients. 600 µg of total serum proteins were focused on linear pH 4–7 IPG strips (18 cm) and then separated on 12.5% polyacrylamide gels, which were stained with Gel Code Blue Stain. (B) The 3D images of some selected statistically significant (*p*<0.05) differentially expressed proteins identified in 2DE. Data is represented as mean ± SE (where n = 20). (C) Representative 2D-DIGE image to compare serum proteome of HC and FM patients. FM and HC samples were labeled with Cy3 and Cy5 respectively, while the protein reference pool (internal standard) was labeled with Cy2. (D) Graphical and 3D fluorescence intensity representations of few selected statistically significant (*p*<0.05) differentially expressed proteins in FM patients identified in biological variation analysis (BVA) using DeCyder 2D software. Graphs showing the decrease/increase in the standardized log abundance of spot intensity in FM compared to the HC cohort of the study (n = 8).

Serum proteome analysis of FM patients and healthy controls by 2DE identified 22 statistically significant (*p*<0.05) differentially expressed (with changes from −4.28 to +78.73-fold) protein spots (Table S1.1). After staining with GelCode Blue Safe Protein Stain, over 700 protein spots were detected reproducibly in each gel by IMP7 software. Representative 2DE images of serum proteome profile of FM subjects and healthy individuals, and bar-diagrammatic representation of the fold change and 3D views of few selected spots are illustrated in Figure 1A and B. In MS analysis 12 different proteins were identified from the 22 differentially expressed protein spots, since in few cases MS analysis revealed similar identity for multiple protein spots appearing as different entities in 2D gels. The similar identity of multiple spots indicates the possibility of presence of various isoforms of those particular proteins probably due to the complex combinations of post-translational modifications. Among the 12 identified proteins; 7 proteins were up-regulated (serum amyloid A, hemopexin precursor, apolipoprotein E, α-1-antitrypsin precursor, leucine-rich α-2-glycoprotein, α-1-BN glycoprotein and α-1-antichymotrypsin precursor) and 5 proteins were down-regulated (haptoglobin, ficolin 3 precursor, apolipoprotein A-I, clusterin precursor and serum albumin) (Figure S2; Table 2 and S2.1). Interestingly, serum amyloid A (spot U13 and U14) was found to be highly over expressed (>25-fold) in all the FM patients.

**Table 2:** List of differentially expressed serum proteins identified in *falciparum* malaria•.

Sl no	Name of protein with UniProtaccession number	Gene name		MW (kDa)	No of spots(spot ID)	Fold change	*p* value (t-test)	Protein score #	Total ion score#	No. of matched peptides#	Molecular functionŝ
**Down-regulated proteins**											
1	(P00738) Haptoglobin ** ^$^ †		HP	45.17	11 (44,46, 47,49.52, 53,55,56, 57,76,79)	1.88–3.25	0.000067–0.048	713	649	14	A, C
2	(P01028) Complement C4 *		C4A/C4B	192.6	2 (51,105)	1.53 & 2.48	0.041 & 0.0021	733	691	23	A, G, I
3	(P00751) Complement factor B (C3/C5 convertase)*		CFB	85.4	1 (120)	1.4	0.0068	717	603	26	A
4	(O75636) Ficolin 3 (Collagen/fibrinogendomain-containing protein 3)**		FCN3	32.9	1 (119)	1.21	0.015	515	463	10	A
5	(P10909) Clusterin (Complement-associatedprotein SP-40)** $		CLU	52.46	2 (42,107)	1.69 & 1.79	0.0011 & 0.0065	210	201	7	A
6	(P02768) Serum albumin ** $		ALB	69.32	4(43.50, 54,78)	1.85–2.59	0.00097–0.0026	1410	1296	26	A, B
7	(P02753) Retinol-binding protein *		RBP4	23.02	1 (40)	1.65	0.019	637	576	11	B, D, F
8	(P49802) Regulator of G-protein signaling 7*		RGS7	75.79	1 (37)	1.36	0.017	28	–	12	A
9	(P02787) Serotransferrin (Transferrin)*		TF	76.99	2 (39,68)	1.28 & 1.41	0.012 & 0.045	1160	906	42	A, B
10	(P27169) Serum paraoxonase/arylesterase 1*		PON1	39.59	1 (41)	1.73	0.014	272	227	11	A, B
11	(P14136) Glial fibrillary acidic protein, astrocyte*		GFAP	49.84	1 (104)	1.52	0.0042	38	23	7	A
12	(P30626) Sorcin (22 kDa protein)*		SRI	21.66	1 (72)	1.97	0.047	27	–	6	A
13	(P02765) Alpha-2-HS-glycoprotein (Fetuin-A)*		AHSG	39.3	1 (45)	1.9	0.0069	495	450	10	A, H, I
14	(P02766) Transthyretin (Prealbumin)*		TTR	15.9	1 (73)	2	0.02	88	76	3	A, B, D
15	(P02647) Apolipoprotein A-I **		APOA1	30.75	1 (121)	2.1	0.0051	486	351	20	A, B, G, H, I
16	(P06727) Apolipoprotein A-IV *		APOA4	45.34	2 (71,109)	1.87 & 2.08	0.016 & 0.024	760	592	24	A,B, G, I
**Up-regulated proteins**											
17	(P02735) Serum amyloid A ** ^$^ †	SAA1& SAA2		13.52	2 (1,2)	28.6 & 50.9	0.000002–0.0069	212	164	7	A
18	(P01009) Alpha-1-antitrypsin (Alpha-1-protease inhibitor)** $	SERPINA1		46.7	7 (4,10,25, 31,36,61, 67)	1.45–2.67	0.00022–0.049	670	538	23	A, H, I
19	(P02750) Leucine-rich alpha-2-glycoprotein ** $	LRG1		38.15	3 (5,6,11)	2.3–2.52	0.00025–0.0052	675	621	11	A
20	(P04004) Vitronectin (Serum spreading factor)* $	VTN		54.27	1 (8)	2.4	0.0017	184	164	7	A
21	(P01011) Alpha-1-antichymotrypsin **	SERPINA3		47.62	5 (29,32, 33,35,66)	1.49–1.67	0.0037–0.043	284	130	20	H, I
22	(Q9H293)Interleukin-17E (IL-17E)*	IL25		20.31	1 (83)	3.55	0.045	28	19	4	A
23	(P04217) Alpha-1B-glycoprotein (Alpha-1-BN glycoprotein)**	A1BG		54.3	1 (118)	1.02	0.0069	900	821	16	A
24	(Q9Y376) Calcium binding protein 39(Mo25 protein)*	CAB39		39.84	1 (116)	1.58	0.011	36	–	9	A
25	(P01834) Ig kappa chain C region* ^$^ †	IGKC		11.6	3 (14, 22, 26)	1.76–2.17	0.00009–0.00032	217	196	4	A, E
26	(P01876) Ig alpha-1 chain C region*	IGHA1		37.63	2 (99, 100)	1.5	0.0049 & 0.041	588	532	12	A, B
27	(P02649) Apolipoprotein E **	APOE		36.13	1 (117)	1.46	0.042	1100	955	21	A, B
28	(P02790) Hemopexin (Beta-1B-glycoprotein)**	HPX		51.64	1 (97)	1.58	0.0084	596	496	21	A, B
29	(P01871) Ig mu chain C region*	IGHM		49.52	1 (30)	1.56	0.028	522	484	15	E
30	(Q9HC96) Calpain 10 (Calcium-activatedneutral proteinase 10)*	CAPN 10		74.89	1 (102)	1.48	0.019	29	–	12	A

•Alterations in protein expression levels in *falciparum* malaria (FM) were measured using healthy subjects as controls.

* Proteins identified in 2D-DIGE experiment.

** Proteins identified in both 2DE and 2D-DIGE experiments.

$ Proteins significant after false discovery rate (FDR) correction (Benjamini-Hochberg).

† Proteins significant after Bonferroni correction.

# For proteins with multiple spots in the 2D gels, representative spot detail is provided. Exact values for each spot are provided in (Table S2).

^ ∧Revealed by GeneSpring software (version 11.5) analysis.

**A**-protein binding; **B**-lipid binding; **C**-hemoglobin binding; **D**-retinol/retinoid binding; **E**-antigen binding; **F**-vitamin transporter activity; **G**-sterol transporter activity; **H**-enzyme regulator activity; **I**-enzyme inhibitor activity.

Around 1300 protein spots were detected on each 2D-DIGE gels in DeCyder 2D software analysis. In 2D-DIGE analysis of FM and HC, total of 121 (around 9.3% of the entire detected spots) differentially expressed spots satisfied the statistical parameters (t-test; *p*<0.05), among which, 70 protein spots were up-regulated (with changes from 1.02 to 50.9-fold) and the remaining 51 were down-regulated (range from 1.2 to 24-fold) (Table S1.2). Out of 121 spots, 36 and 9 spots were found to be statistically significant after performing false discovery rate (FDR) correction (Benjamini–Hochberg) and Bonferroni correction, respectively (Table S1.2). All of the 121 differentially expressed spots (in FM compared to HC) identified in 2D-DIGE analysis were excised and subjected to MALDI-TOF/TOF MS analysis. We obtained reliable MS IDs for 63 protein spots out of 121 (Table S2.2); while remaining 58 spots remained unidentified and produced virtually empty spectra, most likely owing to the presence of extremely diminutive quantity of proteins as indicated by the retrospective scrutiny of the spot volumes. The 63 protein spots identified by MS represented 30 (14 up-regulated and 16 down-regulated) differentially expressed proteins in FM patients (Table 2; Figure 1C and S3). Proteins identified in 2DE experiment were also obtained in 2D-DIGE; additionally, new candidates were also identified by 2D-DIGE due to the higher sensitivity and reproducibility. 3D views and graphical representation of selected protein spots are shown in Figure 1D.

**Figure 2:**
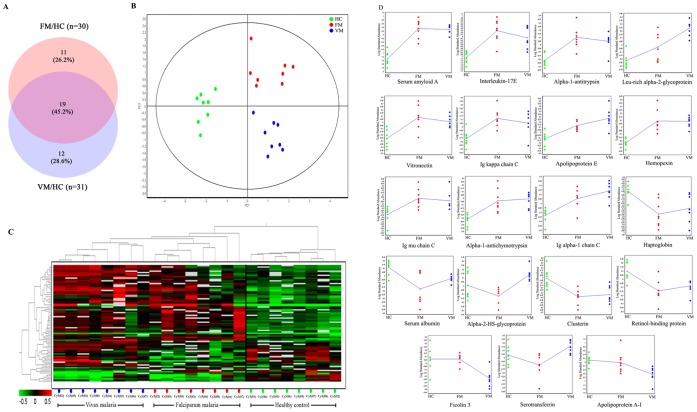
Comparative analysis of *falciparum* and *vivax* malaria. (A) Venn diagram showing the number of proteins differentially expressed in FM and VM, among which 46.2% were found to be common. (B) PCA analysis shows the clustering of different spot maps (green-HC, red-FM, blue-VM group). The PC1 component separates the control group from the rest, and the PC2 clusters the diseased groups separately. Proteins which participated in PCA analysis were present in at least 80% of the spot maps and passed the filter of one-way ANOVA (*p*<0.01) test. (C) The dendrogram showing the separation of different experimental groups after the hierarchical cluster analysis (red - up-regulated, green - down-regulated and black – no significant change in expression level). The spot maps are clustered together for each experimental group (HC, FM and VM). (D) Trend of differentially expressed proteins in malaria patients represented as standardized log abundance of spot intensity in FM, VM and HC cohort of the study. Compared to HC, all of the identified proteins (except serotransferrin and alpha-2-HS-glycoprotein) exhibited similar trend of differential expression in FM and VM; however, fold-change values are different.

A comprehensive comparative analysis of host responses in FM with that of VM (from the findings of our previous study on VM [15]) was carried out to categorize the common and unique proteomic alterations in human serum in *Pf* and *Pv* infections. Almost half (46.2%) of the total identified proteins were commonly modulated in both plasmodial infections; however, the magnitude of proteomic alteration was different in these two types of malaria (Figure 2A and D). Compared to healthy controls, quite a few serum proteins such as calcium binding protein 39, calpain 10, regulator of G-protein signaling 7, serum paraoxonase/arylesterase and transthyretin precursor were found to be differentially expressed in FM but not VM. In contrary, some proteins like ceruloplasmin, vitamin D-binding protein, serum amyloid P, alpha-2-macroglobulin, fibrinogen beta chain precursor exhibited altered expressions only in VM patients (Table S3). Among the 19 proteins, which were differentially expressed (compared to HC) in both of the malaria, only alpha-2-HS-glycoprotein and serotransferrin precursor (transferrin) exhibited opposite trends in *Pf* and *Pv* infections. Rest of the 17 proteins exhibited similar trend of differential expression in FM and VM; although, fold-change values were different (Figure 2D).

Compared to VM, 84 protein spots were found to be differentially expressed in FM (t-test; *p*<0.05). After FDR (Benjamini–Hochberg) and Bonferroni correction 10 and 3 protein spots (out of 84) remained significant, respectively (Table S1.3). Out of 84, MS IDs for 43 protein spots were obtained in MALDI-TOF/TOF MS analysis, which indicated differential expressions of 13 proteins in FM compared to VM. Among those 13 differentially expressed proteins, 5 proteins (interleukin-17E precursor, serum amyloid A, ficolin 3 precursor, alpha-1-antitrypsin and Ig kappa chain C region) were up-regulated while the remaining 8 proteins (alpha-2-HS-glycoprotein, apolipoprotein E, serotransferrin precursor, alpha-1-antichymotrypsin, leucine-rich alpha-2-glycoprotein, AMBP protein, vitamin D-binding protein and haptoglobin) exhibited reduced expression level in *Pf* infected patients (Table S3.4).

Principal component analysis (PCA) using the extended data analysis (EDA) module of the DeCyder software *v*7 revealed distinct clustering of the three experimental groups (FM, VM and HC) (Figure 2B). Proteins present in at least 80% of the spot maps, which passed the filter of one-way ANOVA (*p*<0.01) test were included in this multivariate analysis. Additionally, a hierarchical cluster analysis was performed using the same protein selection criteria (Figure 2C). 85 protein spots were found to be significantly differentially expressed in the malaria subjects compared to the healthy individuals.

Further comparative analysis was performed keeping leptospiral infection as a febrile control to appraise the specificity of the identified malaria related serum markers. Although, some of the identified proteins exhibited similar trends of differential expressions in malaria and febrile controls, interestingly, expression levels of quite a few candidates including serum amyloid A, haptoglobin precursor, ficolin 3 precursor, hemopexin precursor, interleukin-17E precursor, retinol-binding protein, serotransferrin precursor, and vitronectin precursor were found to be altered in malaria patients (both FM and VM) but not in leptospiral infection (Table S4). Altered expression levels of identified serum proteins in *falciparum* and *vivax* malaria and leptospirosis (FC) has been illustrated bar-diagrammatically in Figure S4.

### Validation of the Identified Differentially Expressed Proteins

**Figure 3:**
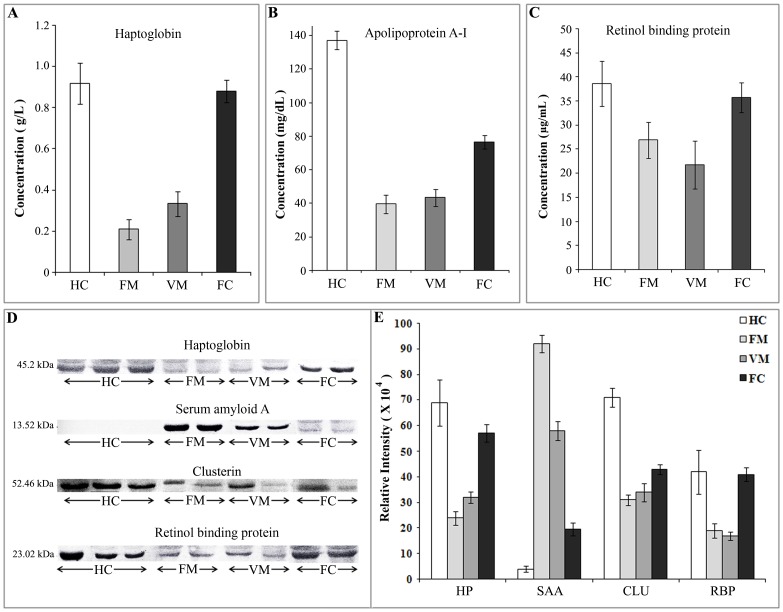
Immunoassay-based validation of differentially expressed proteins. Immunoturbidimetric measurement of serum haptoglobin (A) and Apo A−1 (B) levels in healthy subjects (n = 20), *falciparum* malaria (n = 20), *vivax* malaria (n = 17) and febrile controls (n = 6). Histograms depicting the haptoglobin and Apo A−1 concentrations determined by immunoturbidimetry. Patients with malaria infection found to have lower serum level of haptoglobin and Apo A−1 compared to the HC and FC (*p*<0.0001; Mann-Whitney test). (C) Retinol-binding protein 4 (RBP4) level was measured in sera derived from malaria patients (n = 24), healthy subjects (n = 20) and febrile controls (n = 6) by ELISA and results are shown as bar-diagrams (mean ± SE), which indicates down-regulation of RBP4 in FM and VM patients (*p*<0.01) compared to HC, while serum level of RBP4 found to be unaltered in FC (leptospirosis patients) (D &E) Western blot analysis of haptoglobin (HP) serum amyloid A (SAA), clusterin (CLU) and retinol-binding protein (RBP) from serum samples of HC (n = 12), FM (n = 12),VM (n = 12) and leptospirosis patients (n = 6). Representative blots of the target proteins (D) are depicted along with their respective relative intensities (X 10^4^) (E). Western blot analysis revealed up-regulation of serum amyloid A and down-regulation of haptoglobin, clusterin and retinol-binding protein in FM and VM patients (*p*<0.01) compared to the controls (HC and FC). All data are represented as mean ± SE.

Validation of a few differentially expressed proteins was performed using different immunoassay-based methods including immunoturbidimetric assay, ELISA and western blotting to confirm the results of proteomic analysis. Haptoglobin and apolipoprotein A-I (Apo A-I) concentrations were directly quantified turbidimetrically in the serum samples of malaria patients (n = 37), healthy subjects (n = 20) and febrile controls (n = 6). Compared to the healthy controls, both FM and VM patients found to have lower serum level of haptoglobin and Apo A-I (*p*<0.0001 in a Mann-Whitney test) (Figure 3A and B). The mean haptoglobin concentration was found to be 0.208±0.048 and 0.333±0.06 g/L in FM and VM patients respectively, while the healthy and leptospirosis (FC) populations exhibited a mean values of 0.918±0.1 g/L and 0.888±0.056 g/L (mean ± SE). Likewise, Apo A-I exhibited more than three times lower mean value in both the malaria patients compared to the healthy subjects (39.39±5.43, 43.19±4.96 and 137.05±5.33 mg/dL in FM, VM and HC respectively). While the febrile controls shown a mean value of 76.52±4.12 mg/dL, which is around 2-fold higher than the malaria patients. Serum retinol-binding protein (RBP) concentration was measured by sandwich ELISA. The serum levels of RBP was found to be significantly (*p*<0.01) lower in malaria patients (both FM and VM) compared to the HC and FC groups [38.67±4.69 µg/mL, 35.78±3.09 µg/mL, 26.89±3.78 µg/mL and 21.67±4.99 µg/mL (mean ± SE) in HC, FC, FM and VM respectively] (Figure 3C). Western blot analyses of four differentially expressed targets proteins; haptoglobin, serum amyloid A, clusterin and retinol-binding protein were performed on a subset of control [HC (n = 12) and FC (n = 6)] and diseased samples [FM and VM (n = 12 each)] (Figure 3D). CBB staining of the SDS-PAGE gels and Ponceau staining of the transferred blots containing the resolved proteins indicated equal loading of the samples in each lane (Figure S5). Western blot analysis showed up-regulation of serum amyloid A and down-regulation of haptoglobin, clusterin and retinol-binding protein in FM and VM patients (*p*<0.01) compared to the healthy and febrile controls. These results confirmed our findings from the proteomic analysis, and are illustrated graphically in Figure 3E.

### Interaction Networks and Functional Pathway Analysis

Thirty differentially expressed serum proteins identified in FM patients (compared to HC) were subjected to functional pathway analysis using Ingenuity Pathway Analysis (IPA). Out of those 30 candidates, 27 were eligible for network analysis (focus molecule) based on the IPA Knowledge Base criteria. Two overlapping interaction networks were identified where the highest scoring network included 14 out of the 27 focus molecules, while the second network included 10 focus molecules (Figure S6; Table S5.1). The most significant related functions derived from these overlapping networks included, lipid metabolism (14 proteins, *p* = 2.92E^−09^–5.48E^−03^), inflammatory response (18 proteins, *p* = 1.07E^−11^–5.48E^−03^), molecular transport (15 proteins, *p = *2.92E^−09^–5.48E^−03^), immune cell trafficking (10 proteins, *p* = 9.32E^−08^–4.12E^−03^) and humoral immune response (7 proteins, *p* = 1.10E^−05^–5.48E^−03^). According to this functional pathway analysis, *Pf* infection leads to the alteration of multiple serum proteins involved in diverse essential physiological pathways, including acute phase response (Ratio  = 0.067, *p* = 1.11E^−18^) and primary immunodeficiency signaling (ratio  = 0.048, *p* = 5.1E^−05^) (Table S5.2). Functional analysis of differentially expressed proteins was also performed using Protein ANalysis THrough Evolutionary Relationships (PANTHER) and Database for Annotation, Visualization and Integrated Discovery (DAVID) databases. In PANTHER analysis blood coagulation system (*p* = 4.88E^−05^) was again identified. Moreover, the heterotrimeric G-protein signaling, interleukin signaling pathway and inflammation mediated by chemokine and cytokine signaling pathways were identified as related physiological pathways with statistical significance (*p*<0.05) (Table S5.3). Further, DAVID analysis also confirmed modulation of complement and coagulation cascades (*p* = 1.28E^−04^) in FM (Table S5.4).

According to the molecular function analysis by GeneSpring, most of the differentially expressed proteins identified in FM are related to binding (59.5%) and enzyme regulation activity (24%). A small fraction is involved in transport (9.5%) and antioxidant activity (7%) (Figure S7A; Table S6). Majority of the proteins reside in the extracellular region (61%), while some are located in cell (15%), organelle (11%), macromolecular complex (9%), and lumen (4%) as depicted in Figure S7B by cellular component analysis (Table S6). Biological processes analysis by GeneSpring indicated that identified proteins are involved in following biological process: response to stimulus (20%), biological regulation (20%), localization (14.5%), cellular process (11.5%), metabolic process (9%), immune system (9%), multi-cellular organismal process (8.5%), biogenesis (3%), signaling and development process (<4%) (Figure S7C; Table S6).

**Figure 4:**
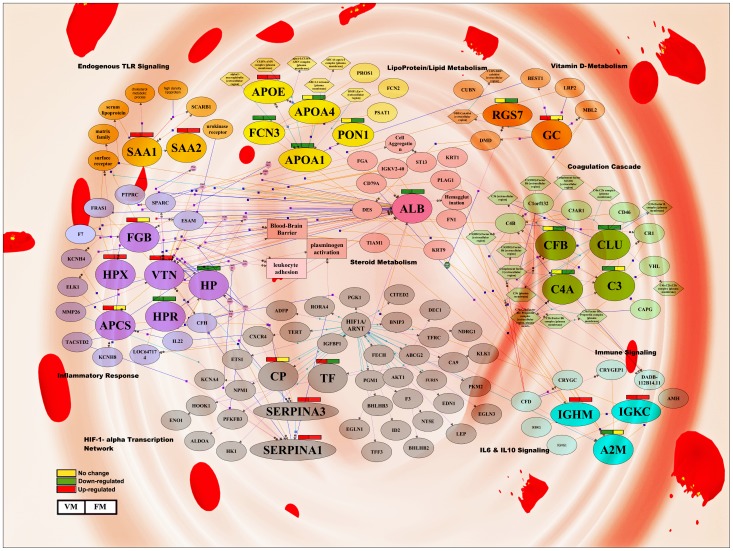
Modulation of essential physiological pathways in *falciparum* and *vivax* malaria. Members of multiple vital physiological processes including acute phase response signaling, chemokine and cytokine signaling, complement cascades, lipid transport and metabolism, and blood coagulation exhibited differential expression in response to *Pf* and *Pv* infection. Differential expression of serum proteins (up-regulated in red, down-regulated in green and no differences in expression level in yellow) are depicted in both plasmodial infections (FM and VM).

Further comparative analysis with VM [15] indicates that both *Pf* and *Pv* infection lead to alteration of multiple serum proteins involved in diverse essential physiological pathways, including acute phase response signaling, chemokine and cytokine signaling, complement cascades, lipid transport and metabolism, and blood coagulation (Figure 4). Table S7 and Figure S8 summarize different biological pathways and physiological functions associated with the differentially expressed serum proteins identified in FM and VM using multiple analytical tools.

### Discrimination of FM, VM and HC using Multivariate Statistical Analysis

Initially, the fidelity of a potential biomarker subset containing 5 proteins identified in 2DE (Table S8.1A) was evaluated for discrimination of FM and HC. As shown in Figure S9A, the two groups (FM and HC) could be clearly classified by phenotype, thereby providing an additional, unbiased estimate of class prediction. Secondly, we applied the class prediction model based on initial cohort (n = 10) to independently predict (assign) the phenotypic class to either FM or HC group on an independent blind group of 16 subjects (8 newly recruited FM patients and 8 HC). The model provided accurate phenotypic classification; and 100% of the FM (n = 19) and 94.74% of the HC (n = 19) subjects were accurately classified into correct phenotypes (Table S8.1C). We achieved 97.37% overall prediction accuracy on independent prediction [HC (n = 19) and FM (n = 19)] using partial least squares discriminant analysis (PLS-DA). For the final validation phase, we compared the performance of the biomarker subset using three well-known machine-learning methods: Decision Trees, Naïve Bayes and support vector machines (SVM). Table S8.1B summarizes the percentage of samples classified during model training, cross-validation and independent prediction respectively, using the three different classifiers. We achieved, 97.37% overall prediction accuracy with SVM, Decision Trees and Naïve Bayes as well, on blinded prediction using the biomarker dataset for FM and HC (n = 19 each).

Further, 7 differentially expressed proteins (Table S8.2A) identified in 2D-DIGE were implicated as potential classifiers for the discrimination of FM and VM patients and healthy subjects employing similar type of analysis (Figure S9B). We achieved 95.83% overall prediction accuracy on blinded prediction (n = 23) using PLS-DA. Table S8.2B summarizes the percentage of the samples classified during model training, cross-validation and independent prediction respectively. In the final validation phase; we achieved 100 and 95.83% overall prediction accuracy with Decision Trees and SVM respectively, followed by Naïve Bayes (91.66%) on blinded prediction using the biomarker dataset for FM (n = 8), VM (n = 7) and HC (n = 8).

**Figure 5:**
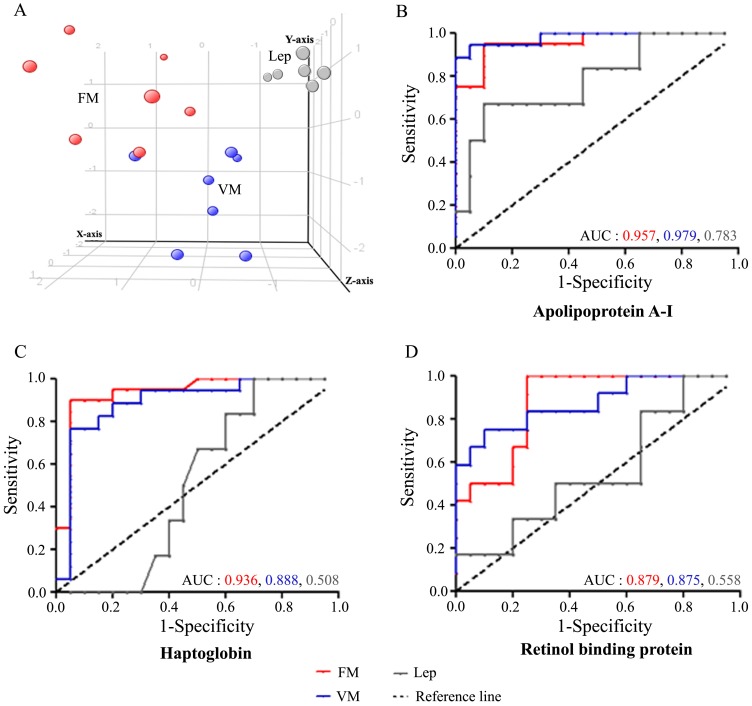
Discrimination of *falciparum* and *vivax* malaria patients from healthy and febrile controls. (A) PLS-DA scores plot for FM (red spheres, n = 6), VM (blue spheres, n = 5) and FC (leptospirosis) (gray spheres, n = 6) samples based on 6 differentially expressed proteins (Table S8.3A) identified using 2D-DIGE. The axes of the plot indicate PLS-DA latent variables. (B–D) Receiver operating characteristic (ROC) curves depicting accuracy of 3 classifier proteins; apolipoprotein A-I (B), haptoglobin (C) and retinol-binding protein (D) for malaria prediction. The area under the ROC curve (AUC) signifies the accuracy of the classifier proteins for distinguishing FM, VM and leptospirosis from healthy controls. AUC value close to 1 indicates an excellent prediction of the disease. The reference line denotes an uninformative test, with an AUC of 0.50.

Next round of multivariate statistical analysis was performed to evaluate the efficiency of the identified serum proteins to discriminate the FM, VM and FC (patients suffering from leptospiral infection) (Figure 5A). 6 differentially expressed proteins (Table S8.3A) identified in 2D-DIGE were implicated as potential classifiers. Table S8.3B summarizes the percentage of the samples correctly classified during model training, cross-validation and independent prediction respectively using different classifiers. We achieved, 100% overall prediction accuracy with Decision Trees; 95.83% with SVM and Naïve Bayes and 91.67% with PLS-DA on independent prediction [FM (n = 8), VM (n = 8) and Lep (n = 6)]. Table S8.1C, S8.2C and S8.3C, provides additional details on the confidence measure obtained on blinded prediction for each subject using a given statistical method. The confidence measure defines the strength of the prediction belonging to the particular class.

### Accuracy of the Classifier Serum Proteins in Prediction of FM and VM

Receiver operating characteristic (ROC) curve analysis was carried out to evaluate the individual performance of 3 classifier proteins; apolipoprotein A-I, haptoglobin and retinol-binding protein in malaria prediction. These 3 serum proteins were used as potential classifiers (along with other 3 candidates) to build statistical sample class prediction models employing PLS-DA and other classification methods for FM, VM, FC and HC discrimination. The area under the ROC curve (AUC) indicates the accuracy of different classifier proteins to distinguish FM, VM and leptospirosis from HC (Figure 5). ROC curves demonstrate apolipoprotein A-I (AUC = 0.957) and haptoglobin (AUC = 0.936) as efficient predictor proteins for *falciparum* malaria detection. A cut off value >112.1 mg/dL for Apo A-I revealed the specificity and sensitivity of 90% and 95%, respectively; while haptoglobin at a cut off value >0.465 g/L provided 95% specificity and 90% sensitivity in predicting *Pf* infection. Retinol-binding protein (AUC = 0.879) exhibited moderate sensitivity (66.6%) and specificity (80%) for FM at a cut off value >30.88 µg/mL (Figure S9C; Table S9). Precondition efficiency of the classifier proteins for *vivax* malaria was also evaluated and found to be highly appreciable for Apo A-I (AUC = 0.979; 94.12% sensitivity and 95% specificity at a threshold value >96.59 mg/dL), haptoglobin (AUC = 0.888; 76.47% sensitivity and 95% specificity at a threshold value >0.45 g/L) and retinol-binding protein (AUC = 0.875; 76% sensitivity and 90% specificity at a threshold value >28.61 µg/mL) as well (Figure S9D; Table S9).

Accuracy of classifier serum proteins in prediction of leptospirosis (FC) was also tested (Figure 5). Although Apo A-I (AUC = 0.783; 66.6% sensitivity and 90% specificity at a threshold value >111.1 mg/dL) exhibited fine proficiency in detection of leptospiral infection; performance of haptoglobin (AUC = 0.508; 66.6% sensitivity and 50% specificity at a threshold value >0.845 g/L) and retinol-binding protein (AUC = 0.558; 50% sensitivity and 65% specificity at a threshold value >34.99 µg/mL) were poor (Table S9). ROC analysis revealed that the serum levels of the classifier proteins, particularly Apo A-I and haptoglobin exhibited good correlation with plasmodial infections and could further be investigated as potential surrogate protein markers for malaria.

## Discussion

Among the four different species of Plasmodia, which cause malaria in human, *Pf and Pv* account for over 90% of the total malaria cases worldwide. In this study, we used proteomics to decipher the alteration in human serum proteome due to the *Pf* infection to gain insight into the disease pathogenesis and host immune response. We also performed a comparative analysis of host response in *Pf* and *Pv* infection. The comparative proteomic analysis of plasmodial and leptospiral infection (febrile control) was performed to appraise the generic febrile responses and specify the malaria related serum markers. The ultimate aspiration of this study was to identify potential marker proteins that can distinguish the malaria patients from the healthy or febrile controls as well as discriminate between the *Pf* and *Pv* infections with high accuracy. Although a number of earlier proteomic and immunoassay-based studies have demonstrated the alteration of limited set of serum proteins in malaria [16]–[18] hitherto, there was no attempt for a comprehensive analysis to establish a panel of classifier proteins that can readily discriminate the FM and VM groups from the controls.

Our results indicate that various vital physiological pathways, including acute phase response signaling, cytokine and chemokine signaling, complement cascades, lipid transport and metabolism, and blood coagulation are modulated in *Pf* and *Pv* infections (Figure 4). Alteration of the levels of several acute phase proteins (APPs) and multiple members of serum complement cascade as well as complement regulatory proteins (Table 2) due to the plasmodial infections is consistent with earlier findings [19]–[21]. Increased expression levels of circulating acute-phase amyloid proteins like serum amyloid A and P provide non-specific resistance against the pre-erythrocytic stages of Plasmodium, limit tissue damage and promote a rapid return to homeostasis [22], [23]. Interestingly, human serum paraoxonase (PON1) an HDL-associated esterase which protects lipoproteins against oxidation, found to be down-regulated in *falciparum* malaria patients. Acute inflammatory stimuli lead to reciprocal regulation of SAA and PON-1 [24]. Decreased serum PON-1 activity in context with *falciparum* malaria may in part be attributable to higher SAA level.

In course of the disease progression, malarial parasites growing in the erythrocytes degrade hemoglobin and generate reactive oxygen species (ROS), which lead to increased oxidative stress within the erythrocytes and outside the parasitized cells. As a result, to circumvent the situation, enhanced plasma levels of antioxidant defense associated enzymes/proteins such as superoxide dismutase-1 (SOD-1) are observed in acute malaria patients [25]. In both FM and VM patients we have identified elevated serum level of hemopexin, a heme-binding protein, which provides the second line of defense against hemoglobin-mediated oxidative damage during intravascular hemolysis [26]. Increased production of this acute phase protein by the host defense system could be helpful to circumvent the pro-inflammatory response with oxidative stress generated in patients with *Pf* or *Pv* infection. In contrast, haptoglobin (Hp) found to be significantly down-regulated in FM and VM patients. Hp removes free hemoglobin (Hb) released during parasite induced hemolysis, and disappears as the Hp-Hb complexes leading to the malaria associated hypo-or ahaptoglobinemia and is a promising inflammatory marker to evaluate the severity of the Plasmodium infection [21]. Earlier reports have demonstrated the possible role of this APP as an epidemiological marker for malaria [21], [27].

Erythrocyte invasion is an essential gateway to malaria disease and a key target for disease intervention. Signaling via the erythrocyte beta 2-adrenergic receptor and heterotrimeric guanine nucleotide–binding protein (Gαs) regulates the entry of the human malaria parasite *P. falciparum*. Disruption of the interaction between the G-alpha-s subunit of the Gs protein and the receptor results in a reduced erythrocyte invasion by the parasite and subsequent low level of parasitemia [28]. Down-regulation in regulator of G-protein signaling in FM patients might be due to some host response to combat this parasitic infection. Conversely, up-regulation of apolipoprotein E was observed in the malaria patients. This apolipoprotein also inhibits Plasmodium invasion, since it shares the cell entry mediators (heparan sulphate proteoglycans and/or low density lipoprotein receptor) with the parasite [29]. The pathway analyses and densely connected networks based on our results provide an insight into the underlying molecular mechanisms of malaria.

Early and accurate diagnosis is critical for the effective treatment and management of malaria. In recent years, multivariate projection methods are being successfully applied to analyze biological data obtained through genomic, transcriptomic or proteomic approaches to study various human diseases, with implications for diagnostics and clinical management [30], [31]. A sub-set of the proteins identified in our proteomic analysis was used to build statistical sample class prediction models to identify the classifier marker proteins for FM, VM and HC discrimination. Interestingly, two key classifier proteins: serum amyloid A and haptoglobin differentially expressed consistently in all of the malaria patients (FM and VM) compared to the control subjects (HC and FC) and remained statistically significant after FDR (Benjamini-Hochberg) and Bonferroni correction of the *p*-values obtained in t-test; indicating very strong correlation between the expression levels of these two serum proteins and plasmodial infections. The recognition ability of the prediction models for FM, VM and HC discrimination and cross-validation was almost 100% (Table S8). We controlled for the statistical false discovery rate using three distinct, iterative validation steps: (i) k-fold cross-validation algorithm for the original cohort, (ii) application of the marker subset identified in the original cohort to classify newly recruited patients, and (iii) performance of the marker subset validated using three well-known machine learning methods. In our study, biological replicates were investigated, i.e. each proteome profile was representative of a different human subject, and hence the data-sets are characterized by low homogeneity, conferring to the protocol a very high level of variability and complexity. Indeed the extreme heterogeneity or large biological variations including gender, age, genetic factors, dietary considerations, environmental factors and drug treatment affects the detection, validation and establishment of “gold standard” serological biomarkers [10], [32]. Nonetheless, the accurate discrimination among the FM, VM and control groups obtained by various prediction models on the basis of differentially expressed candidate proteins testifies to the excellent potential of this analytical approach for the detection and discrimination of VM and FM (Figure 5; Figure S9). It should also be noted that uncomplicated FM and VM patients with diverse range of parasitemia; mainly low and moderate parasitemic (<5000 parasites/µL blood), were used for the validation of the prediction models (Table 1). Even so, the discrimination accuracy of the study is very appreciable indicating the capability of our analytical approach for the detection of very low-level of parasitemia, which is highly promising from a diagnostic point of view. Although, diagnosis of malaria on the basis of microscopic examination of thin or thick smears of peripheral blood is the most commonly used and well-accepted method, but it requires highly trained personnel for smear interpretation, frequently fails to distinguish mixed-species infections or diagnose patients with “sub-microscopic” parasitemia below the detectable limit of blood smears, and in many areas of endemicity the operating characteristics of microscopy are poor [33], [34]. In quest of an early and accurate diagnosis of malaria and discrimination of *Pf*, *Pv* or mixed infection, establishment of serum protein markers can be an attractive approach apart from clinical symptoms and conventional microscopic examination of blood smears. To this end, some of the classifier candidate proteins identified in this study; such as serum amyloid A, paraoxonase, apolipoprotein A-I and E, haptoglobin, hemopexin, and complement C4 are very important due to their functional relevance in malaria pathogenesis and could further be investigated as potential surrogate protein markers for clinical implications. Various rapid diagnostic tests (RDTs) are in practice for malaria diagnosis, which diagnose the infection on the basis of detection of parasite proteins/antigens e.g. histidine-rich protein II (HRP-II) or lactate dehydrogenase (LDH) [35], whereas for the first time we have demonstrated the discrimination between FM and VM patients based on protein expression in human host. Malaria RDTs are used regularly in clinics due to the low cost, sensitivity and less detection time. However, analysis of frozen specimens of blood from parasitaemic patients using existing RDTs is bit challenging. Another limiting factor is the shelf-life of RDTs, since most of the existing RDTs deteriorate rapidly on exposure to moisture (humidity) and high temperature. Moreover, significant variations may appear between technicians in both RDT preparation and result interpretation process depending on experience of the performer, manual proficiency and visual perception [36]. To this end, serum protein markers can be potential candidates for development of an alternative sensitive diagnostic approach for malaria. Development of highly sensitive biosensors for the identified surrogate proteins might be attractive from a diagnostic point of view.

In summary, the present study demonstrates the application of diagnostic proteomics to decipher host responses against the human malaria parasites *Pf* and *Pv*, and identifies potential candidate biomarkers for these two plasmodial infections. In this comprehensive proteomic analysis we have identified multiple differentially expressed serum proteins with versatile biological functions, indicating the modulation of multiple vital physiological pathways in FM and VM patients. We anticipate that information obtained from this study will provide valuable insight into the underlying molecular mechanisms of malaria and may help to establish early detection surrogates for these infectious parasitic diseases to meet the need for better diagnostics and effective therapy. Some of our identified classifier proteins such as serum amyloid A, apolipoprotein A-I and E and haptoglobin, which successfully discriminated FM from VM might be prognostic host markers for disease severity. To this end, it would be interesting to elucidate the fate of the identified serum proteins in severe malaria patients and could be a future continuation of this study. Diagnostic impact of the identified serum biomarkers in clinics and specificity for malaria prediction can only be established after investigation of the disease patterns in large clinical cohorts.

## Materials and Methods

### Subject Selection and Sample Collection

This proteomic analysis was performed with the approval of the institutional ethics committee of Seth GS Medical College and King Edward Memorial hospital, Parel, Mumbai, India. Patients suffering from uncomplicated *Pf* or *Pv* infection with asexual parasite count more than 1000 per µL of blood were selected for this study. A total of 37 patients, with uncomplicated FM (n = 20) or VM (n = 17) confirmed through microscopic examination of a thin peripheral blood smear followed by RDT were enrolled for this proteomic study. In addition, blood specimens were collected from age and sex matched leptospirosis patients (n = 6) as febrile controls, and healthy subjects (n = 20) to perform comparative proteomic analysis. Written informed consent was taken from each participant (malaria patients and controls) prior to the sample collection process. Demographic, epidemiological and clinical details of all malaria patients and febrile controls (FC) selected for this proteomic study are provided in Table 1. Blood samples (5.0 mL) were collected from the antecubital vein of the subjects using serum separation tubes (BD Vacutainer®; BD Biosciences). Immediately after blood collection the tubes were kept in ice for 30 mins for clotting. Serum separation was performed as described previously [15]. In brief, after clotting, the samples were centrifuged at 2500 rpm at 20°C for 10 mins and serum was collected carefully from the upper surface. Collected serum was divided into multiple aliquots and stored at −80°C until time of analysis to prevent protein degradation. Prior to proteomic analysis, maximum 2–3 freeze/thaw cycles were allowed for any serum sample to reduce pre-analytical variations.

### Processing of Serum Samples and 2DE

Crude serum was diluted five times with phosphate buffer (pH 7.4) and subjected to mild sonication in a Vibra cell sonicator using the following settings: 6 cycles of 5 sec pulse; 30 sec gap in between; at 20% amplitude. After sonication, the top two high-abundance serum proteins (albumin and IgG) were removed using Albumin & IgG Depletion SpinTrap (GE Healthcare) following the manufacturer’s instructions. Extraction of protein from depleted serum samples was performed employing TCA/acetone precipitation method as described by Chen et al., with slight modifications [37]. In brief, depleted serum samples were diluted (1∶4 ratio) with ice-cold acetone containing 10% (w/v) TCA. Uniform mixing was performed using mild vortexing for 15 sec and the mixture was allowed to incubate at −20°C for 2 hrs for protein precipitation. After completion of the incubation period, tubes were centrifuged at 1000 g for 15 min at 4°C. Supernatants were separated and kept in fresh microcentrifuge tubes, and the pellets were dissolved in rehydration buffer [8 M urea, 2 M thiourea, 4% (w/v) CHAPS, 2% (v/v) IPG buffer (pH 4–7; Linear), 40 mM DTT and traces of bromophenol blue]. In order to precipitate the remaining amount of proteins present in the collected 10% TCA/acetone-containing supernatants, 1 mL ice-cold acetone was added to each tube and the samples were subjected one additional round of precipitation and extraction process. In all cases, prior to re-suspension in rehydration buffer, the pellet was briefly air-dried. Prior to proteomic analyses, protein concentration in the samples was quantified using the 2D-Quant kit (GE Healthcare) following the manufacturer’s instructions. A total of 600 µg of depleted serum protein extract dissolved in 350 µL of rehydration buffer was loaded on 4–7 pH range IPG strips (18 cm) and underwent passive rehydration for 14–16 hrs. Isoelectric focusing (IEF) was performed on an Ettan IPGphore 3 isoelectric focusing unit (GE Healthcare) for overall approximately 78 kVh using the following voltage settings: 200 V for 4 h (step and hold), 500 V for 1 h (step and hold), 1000 V for 1 h (step and hold), 8000 V for 3 h (gradient), and 8000 V for 7∶30 h (step and hold). After completion of IEF, the focused IPG strips were stored at −20°C until the second dimensional analysis was performed. Preceding to the second dimensional separation, each strip was equilibrated to reduce and alkylate the proteins (for 15 min each) using equilibration buffer containing 6 M Urea, 75 mM Tris-HCl pH 8.8, 29.3% (v/v) glycerol, 2% (w/v) SDS, and 0.002% (w/v) bromophenol blue. Just prior to use, 1% (w/v) DTT or 2.5% (w/v) IAA was added in the first (reducing) and second (alkylating) equilibration buffer, respectively. The second dimension was performed on 12.5% SDS polyacrylamide gels using an Ettan DALTsix electrophoresis unit (GE Healthcare). After electrophoresis GelCode Blue Safe Protein Stain (Thermo Scientific, USA) was utilized for visualization of the protein spots. Proteins extracted from each of the subjects were run in duplicate to verify the reproducibility and curtail technical artifacts.

### 2D-DIGE

Each CyDye (Cy3, Cy5 and Cy2) was resuspended in anhydrous N, N-dimethylformamide (DMF) to prepare a stock dye concentration of 1 mM. A working solution of 400 pmol of each CyDye was made by further dilution of the stock with DMF. Samples (test and control) were labeled with Cy3 and Cy5, while a mixture of equal amounts of all samples to be analyzed in the experiment, regarded as internal standard, was labeled with the third fluorescent dye; Cy2 according to the manufacturer’s instructions (GE Healthcare). In brief, the pH of each sample was adjusted to 8.5 using 100 mM NaOH. 50 µg of each protein sample [malaria, controls (HC/FC) and internal standard] were separately labeled with 400 pmol of CyDyes. After addition of CyDyes, samples were incubated on ice for 30 in the dark. Labeling reaction was stopped by addition of 10 mM lysine followed by incubation on ice for additional 10 min. Dye-swapping was performed while labeling the test and control samples for eliminating any type of dye effects. After labeling, samples labeled with Cy3, Cy5 and Cy2 were mixed, diluted with the rehydration buffer and loaded on 18 cm, 4–7 pH IPG strips. Subsequent IEF and SDS-PAGE separation were performed following the same protocol as previously described in the 2DE section.

### Image Acquisition and Software Analysis

Image acquisition and data analysis was performed as described previously [15]. In brief, after staining, the 2D gels were scanned by using LabScan software version 6.0 (GE Healthcare) and analyzed by using ImageMaster 2D Platinum 7.0 software (GE Healthcare). Comparative analysis of FM samples was performed by creating different “match sets” and using the HC samples as reference. Spot detection parameters were specified as: Smooth: 7, Saliency: 100 and Min Area: 5. After automatic detection of the spots through IMP7, manual refinement was performed to eliminate any contaminating artifacts, such as streaks or dust particles. Spot quantification was performed in % vol value using ImageMaster algorithm. It provided normalized value that remains relatively independent of variations due to staining or protein loading. The gel analysis tables, histograms and 3D images generated by the software were used for further analysis.

2D-DIGE gels were scanned using Typhoon 9400 variable mode imager (GE Healthcare) at a 100 µm resolution employing suitable excitation/emission wavelengths for each of the CyDye [Cy3 (523/580 nm), Cy5 (633/670 nm) and Cy2 (488/520 nm)]. After scanning, gel images were cropped properly using ImageQuant software; version 5.0 (GE Healthcare) prior to importing in DeCyder 2D software; version 7.0 (GE Healthcare) for comparative analysis and relative protein quantification across the FM and control samples. Comparative analysis was performed using two different modules, differential in-gel analysis (DIA) and biological variation analysis (BVA) of the DeCyder software. Preliminary analysis was performed using DIA module to detect spots on a cumulative image derived from merging up to three individual images from an in-gel linked image set (malaria, controls and internal standard). It permits the pair-wise comparisons of each normal and malaria samples to the mixed standard present in each gel and offers spot-wise protein abundance as ratios. Further analysis was performed using BVA module to get the variation in protein expression levels between any of the two experimental groups (FM vs. VM, FM vs. HC and VM vs HC) across all the sets. Statistical significance of the average ratio of expressions was analyzed by Student’s t-test. Protein spots exhibiting differential expression with reproducibly and statistical significance (*p*<0.05) were considered for further analysis. Bonferroni correction (for reducing Type I errors) of the *p*-values obtained from Student’s t-test was performed using standard Bonferroni procedure to recognize those marker proteins which have very strong connection with the diseased state (remains significant after Bonferroni correction). Since Bonferroni correction is extremely conservative; comparatively less stringent false discovery rate (FDR) correction was also performed as detailed in Benjamini and Hochberg [38].

We also performed a comparative analysis of FM data-set obtained in this study with our previously published VM data [15]. Clustering of the three experimental groups (FM, VM and HC) was performed by principal component analysis (PCA) using an algorithm included in the extended data analysis (EDA) module of the DeCyder software. Proteins present in at least 80% of the spot maps and passed the filter of the one-way ANOVA (*p*<0.01) test were included in this multivariate analysis. Additionally, a hierarchical cluster analysis was performed using the same protein selection criteria.

### In-gel Digestion

Statistically significant (t-test, *p*<0.05) differentially expressed proteins spots identified in regular 2DE and 2D-DIGE experiments were selected for further MS analysis to establish protein identity. GelCode Blue stained preparative gels containing much higher amount of protein (1 mg) were used for excision of the spots of interest specified in the 2D-DIGE experiment. Spot excision was performed manually. In-gel digestion of the proteins separated by 2D gel electrophoresis was performed as described by Shevchenko et al., with slight modifications [39]. In short, gel slices were cut into small cubes (∼1×1 mm) and washed with 50 µL of stain removal solution (25 mM ammonium bicarbonate buffer) for removal of CBB stain. After washing, 50 µL of 25 mM ammonium bicarbonate/acetonitrile (1∶1 v/v) was added, followed by 5 min incubation with occasional vortexing at room temperature. After incubation, the solutions were removed. These two steps are repeated for three times. Then, 50 µL reduction solution (10 mM DTT in 100 mM ammonium bicarbonate) was added and the gel pieces were incubated for 60 mins at 56°C in an air thermostat. Tubes were allowed to cool to room temperature after incubation, and 50 µL of 25 mM ammonium bicarbonate buffer was added to wash the gel pieces followed by dehydration with 25 mM ammonium bicarbonate/acetonitrile (1∶1, v/v). After this step, alkylation solution (50 mM IAA in 100 mM ammonium bicarbonate) was added and the tubes containing the gel pieces were incubated for 30 mins at room temperature in dark. Rehydration and dehydration steps were performed twice and gel pieces were allowed to dry. Once the gel slices were properly dried, trypsin solution (Trypsin Gold; Promega, Madison, Wisconsin, United States) was added to the gel pieces keeping the ratio of trypsin: protein around 1∶10 (w/w) and incubated at ice for 30 mins for absorption of the solution. After this step, the tubes were incubated overnight at 37°C. Adequate amount of ammonium bicarbonate buffer was added to cover the gel pieces. Extraction of the digested peptides from the gel matrix was performed using 100 µL of extraction buffer (0.2% formic acid in 66% acetonitrile) after completion of the enzymatic reaction. Extraction step was repeated thrice to ensure maximum recovery of the digested peptides. The collected supernatants were pooled in a single tube and concentrated using speed vac. After extraction trypsin digested samples were further processed using Zip-Tip C18 pipette tips (Millipore, USA) according to the manufacturer’s protocol for removal of salts and enrichment of the peptides.

### MALDI-TOF/TOF Analysis and Protein Identification

Subsequent to enrichment and purification through the Zip-Tip pipette tips, peptide mixtures were dissolved in 0.5 µL of CHCA matrix solution (5 mg/mL CHCA in 50% ACN/0.1% TFA) and spotted onto a freshly cleaned MALDI target plate. Spots were allowed to dry for 30 mins at room temperature. After air drying, the crystallized spots were analysed using a 4800 MALDI-TOF/TOF mass spectrometer (AB Sciex, Framingham, MA) linked to 4000 series explorer software (version 3.5.3). All mass spectra were recorded in a reflector mode within a mass range from 800 to 4000 Da, using a Nd:YAG 355 nm laser. The acceleration voltage and extraction voltage were kept at 20 kV and 18 kV respectively. Six point calibration of the instrument was automatically performed by a peptide standard Kit (AB Sciex) that included des-Arg1-bradykinin (*m*/*z* 904.468), Angiotensin I (*m*/*z* 1296.685), Glu1-fibrinopeptide B (*m*/*z* 1570.677), ACTH (18–39, *m*/*z* 2465.199), ACTH (1–17, *m*/*z* 2903.087), and ACTH (7–38, *m*/*z* 3657.923). All the MS spectra were obtained from accumulation of 900 shots. MS/MS spectra were acquired for the 15 most abundant precursor ions, with a total accumulation of 1500 laser shots and collision energy of 1 kV. Once the MS survey scans were completed, the data were processed to generate a list of precursor ions for interrogation by MS/MS. The combined MS and MS/MS peak lists were searched using the GPS™ Explorer software version 3.6 (AB Sciex). Protein identification was performed by MS/MS ion search using MASCOT version 2.1 (http://www.martixscience.com) search engine against the Swiss-Prot database. Searches were carried out with the following parameters; all entries taxonomy, trypsin digestion with one missed cleavage, fixed modifications: carbamidomethylation of cysteine residues, variable modifications: oxidation of methionine residues, mass tolerance 150 ppm for MS and 0.4 Da for MS/MS. Identified proteins having at least two unique matched peptides were selected for further analysis. We have reported only those proteins with a protein identification confidence interval of ≥95%.

### Immunoturbidimetric Assay

Quantitative immunological measurement of two of the differentially expressed proteins identified in this study; haptoglobin and apolipoprotein A-I, in serum samples of healthy controls (n = 20), *falciparum* malaria (n = 20) and leptospirosis patients (n = 6) were performed using COBAS INTEGRA 400 PLUS system (ROCHE). The serum concentration of those two target proteins in *vivax* malaria was taken in account for a comparative analysis from our previous report [15]. Crude individual serum samples were subjected directly to immunoturbidimetric quantification using the Tina-quant ver.2 kits (Roche Diagnostics) according to the manufacturer’s instructions. Samples and controls were automatically prediluted 1∶21 with NaCl solution by the instrument. In this immunological assay the target proteins form precipitates with the specific antiserum which are determined turbidimetrically at 340 nm. Anti-human haptoglobin (rabbit) and Apo A-I (sheep) antibodies were applied for the immunoturbidimetric quantification of haptoglobin and Apo A-I respectively. The instrument was monitored at absorbance measuring mode where the absorbance increase was directly proportional to the concentration of the target proteins.

### ELISA

Quantification of another interesting target; retinol-binding protein (RBP) was performed using ELISA. Concentrations of RBP4 in serum samples of HC (n = 20), FC (n = 6), FM (n = 12) and VM (n = 12) patients were measured using AssayMax Human Retinol-Binding Protein-4 (RBP4) ELISA kit (Cat# ER3005-1) from AssayPro (USA) following the manufacturer’s instructions. Briefly, quantitative sandwich enzyme assay was employed where RBP4 standard and serum samples (HC, FC, FM and VM) at a dilution of 1∶100 were subjected to a microplate pre-coated with a polyclonal antibody specific for RBP4. Samples were sandwiched by the immobilized antibody and biotinylated polyclonal antibody specific for RBP4, which was recognized by a streptavidin-peroxidase complex. Color development was performed through the addition of a peroxidase enzyme substrate and optical densities were measured at 450 nm and 570 nm using a SpectraMax M2^e^ (Molecular Devices, USA).

### Western Blot Analysis

Prior to the western blotting experiment protein concentration in each sample [malaria patients (n = 24), FC (n = 6) and HC (n = 12)] was accurately estimated using the 2D-Quant kit (GE Healthcare) and BCA Protein Assay (Thermo Fisher Scientific). Western blot analysis was performed as described previously [40]. Briefly, serum proteins were separated by 12% SDS-PAGE (50 µg per track) and then transferred onto PVDF membranes under semidry conditions by using ECL semi-dry transfer unit (GE Healthcare). Western blot was performed by using monoclonal/polyclonal antibody against serum amyloid A (Santacruz Biotechnology, sc-20651), haptoglobin (Santacruz Biotechnology, sc-71207), clusterin (Santacruz biotechnology, sc-8354) and retinol-binding protein (RBP) (Santacruz Biotechnology, sc-69795) and appropriate secondary antibody conjugated with HRP (GeNei (MERCK)-621140380011730 or 621140680011730). Candidate proteins for validation were selected on the basis of fold changes, possible association of the proteins with malaria pathobiology and accessibility of the required antibodies. ImageQuant software; version 5.0 (GE Healthcare) was applied for quantitation of signal intensity of the bands in western blots.

### Proteins Networks and Functional Analysis

Differentially expressed serum proteins in FM were subjected to functional pathway analysis using IPAversion 9.0 (Ingenuity® Systems, www.ingenuity.com) to determine association of the identified proteins with various physiological pathways. The significance of association between our dataset and identified networks/pathways was considered on basis of two parameters, ratio and *p*-values. Differentially expressed proteins in FM patients were also analyzed using PANTHER system; version 7 (http://www. pantherdb.org) [41] and DAVID database version 6.7 (http://david.abcc.ncifcrf.gov/home.jsp) [42], [43]. The list of UniProt Accession from each dataset was uploaded in tab delimited text format at once, which was mapped against the reference *Homo sapiens* dataset to extract and summarize functional annotations associated with individual or group of genes and proteins.

The gene ontology (GO) categories for 30 proteins was assigned using GeneSpring software package (version 11.5; Agilent Technologies, Santa Clara, USA). Since, GO vocabulary is organized in a hierarchical fashion, the second level of GO terms were presented as a balance between GO term for specificity and maximal coverage. GO terms that were enriched in two or more proteins were considered. In addition, a significance *p-*value of the enrichment was computed using the hypergeometric probability distribution, which identifies GO categories represented by the 30 proteins relative to their representation on the Biological Genome for Human created using information available at NCBI (ftp://ftp.ncbi.nlm.nih.gov/gene/DATA).

To determine the biological pathways with significant enrichment of the input proteins, algorithms in GeneSpring software package performs a standard hyper-geometric calculation to obtain *p*-value, which signifies the enrichment. Prior to analysis, manually curated biological pathways from Reactome, Biocarta, NCI and PathwayCommons (in Biopax level 2 format) were populated in GeneSpring’s database. Pathways with significance *p*-value (*p*<0.05) were chosen for subsequent analysis and interpretation. We used GeneSpring’s pathway database to create Shortest Connect network from the selected pathways. The Expand Selection algorithm was performed on the above network to include first and second degree neighbors. Expand Selection uses the GeneSpring pathway database for finding expansion on entities and takes a series of expansions to connect processes/functions/other biomolecules to the given entities (proteins). This algorithm allows listing all processes and functions in which the given entities participate.

### Multivariate Statistical Analysis and Development of Statistical Classifier

We applied proteomics data obtained from 2DE and 2D-DIGE analyses to discriminate among FM, VM, FC and HC groups using multivariate statistical analysis. 5 proteins (haptoglobin, apolipoprotein A-I, hemopexin, apolipoprotein E and serum amyloid A) identified by 2DE (Table S8.1A) and 7 proteins (haptoglobin, apolipoprotein A-I, hemopexin, apolipoprotein E serum amyloid A, serum amyloid P and serum paraoxonase/arylesterase 1) identified by 2D-DIGE (Table S8.2A) were used to develop statistical classifier designed to categorize and predict clinical phenotypes (i.e., FM, VM and HC). Discrimination of malaria (FM and VM) from FC (leptospirosis) and statistical sample class prediction was performed on the basis of differential expression levels of 6 candidate proteins (serum amyloid A, hemopexin, apolipoprotein E, haptoglobin, retinol-binding protein and apolipoprotein A-I) (Table S8.3A). Selection of the candidate proteins was executed on the basis of their level of differential expression and ability to discriminate between the clinical phenotypes. For multivariate statistical analysis and machine learning, the data were mean centered; scaled and logarithmic transformation was performed in order to lower relatively large differences among the respective spot abundances. 2DE data was additionally normalized using Quantile method to correct for batch difference. 3 levels of validation were used to establish the reliability of identified differentially expressed proteins to detect correct phenotypic classes using Mass Profiler Professional (MPP).

We used PLS-DA, SVM, Decision Trees and Naïve Bayes implemented in MPP software package (version 2.2, Agilent Technologies, Santa Clara, USA) for all multivariate and machine learning analysis in this study. Partial least squares is a regression method using the information contained in X data matrix (predictor variables) to predict the behavior of Y data matrix (response variables). PLS method models both X and Y variables simultaneously to find the latent variables in X that will predict the latent variables in Y [44]. The application of PLS as a classification method is indicated as PLS-DA [45], [46]. SVM separates two classes by generating the hyperplane (in a high-dimensional feature space) which maximizes the distance from the hyperplane to the closest training examples [47]. In Decision Trees a sample gets classified by following the appropriate path down the decision tree. The Naive Bayesian model is built based on the probability distribution function of the training data along each feature. Since Decision Trees and Naïve Bayes directly handle multi-class problems, we have used the default parameters for these techniques. The SVMs are trained using sequential minimal optimization with a linear kernel. Validation of the obtained predictive models was performed using a standard K-fold cross-validation procedure: observations in input data were randomly divided into three equal parts, two parts were used for model training, and the remaining samples were classified using the constructed model. The whole process was repeated for 10 times.

### Receiver Operating Characteristic (ROC) Analysis

Efficiency of 3 classifier proteins; haptoglobin, apolipoprotein A-I and retinol-binding protein for prediction of malaria (FM and VM) and leptospirosis (febrile control) was analyzed using receiver operating characteristic (ROC) curves [plot of true positives (sensitivity) vs false positives (1- specificity) for each possible cutoff] using GraphPad Prism software package (version 5.02). ROC curve analysis was performed for only those 3 classifier proteins (out of 6) for which absolute serum concentration values (immunoturbidimetric assay/ELISA) were measured. Sensitivity and specificity values for the marker proteins were calculated at different threshold points. Two-sided *p-*values less than 0.05 were considered statistically significant.

## Supporting Information

Figure S1 **Evaluation of the depletion efficiency for albumin and IgG from human serum.** Two major high-abundance serum proteins; albumin and IgG were removed using Albumin & IgG Depletion SpinTrap (GE Healthcare) to reduce the dynamic range of serum protein concentration. (A) Levels of albumin and IgG in CBB stained 2D gel before and after depletion. 600 µg of total serum proteins were focused on linear pH 4–7 IPG strips (18 cm) and then separated on 12.5% polyacrylamide gels. Depletion of the top two high-abundance proteins (albumin and IgG) introduced nearly two-fold increase in overall spot number in 2D gels. (B) Levels of albumin and IgG in CBB stained 1D-SDS-PAGE gel before and after depletion showing the efficiency of the depletion process. 10 µg of total crude [C] and depleted [D] serum proteins were loaded onto each lane and separated on 10% polyacrylamide gels. (C) Densitometric analysis of the 1D-SDS-PAGE gels revealed around 85% and 80% depletion of albumin and IgG respectively.
(PDF)

Figure S2 **Trends of differentially expressed proteins in** ***falciparum*** **malaria patients visualized in 2DE gels.** (A) Representative 2D gels of serum from healthy controls and FM patients. 600 µg of total serum proteins were focused on linear pH 4–7 IPG strips (18 cm) and then separated on 12.5% polyacrylamide gels, which were stained with Gel Code Blue Stain. Protein spots exhibiting significantly altered expression levels are marked on the gels. Down (B) and up (C) -regulation of protein expression levels in FM patients. The 3D images of statistically significant (*p*<0.05) differentially expressed spots were analyzed using IMP7 software. Data is represented as mean ± SEM (where n = 20).
(PDF)

Figure S3 **Trends of differentially expressed proteins in** ***falciparum*** **malaria patients visualized in 2D-DIGE gels.** (A) Representative 2D-DIGE image comparing the serum proteome of healthy subjects and FM patients. Differentially expressed protein spots identified in FM are marked on the gel. Graphical and 3D fluorescence intensity representations of statistically significant, MS identified protein spots down (B) or up-regulated (C) in FM patients (*p*<0.05) obtained in biological variation analysis (BVA) using DeCyder 2D software. Graphs showing the decrease/increase in the standardized log abundance of spot intensity in the FM cohort of the study.
(PDF)

Figure S4 **Comparative serum proteome analysis of** ***falciparum*** **malaria (FM),** ***vivax*** **malaria (VM) and febrile control (Leptospirosis).** Bar-diagrams showing altered expression levels of different serum proteins in malaria (FM and VM) and febrile control (Leptospirosis patients). * Indicates “no differential expression” (alterations in expression level not statistically significant). Alterations in protein expression levels in malaria and leptospirosis patients were determined using healthy subjects as controls. Fold change values were calculated by keeping the expression level of the proteins (mean value) in healthy population as baseline.
(PDF)

Figure S5 **Equal loading of protein samples during western blot experiment.** Representative CBB stained SDS-PAGE gel (A) and Ponceau stained blot (B) containing the resolved proteins depicting equal loading (50 µg) of the samples [malaria patients (FM and VM) and controls (FC and HC)] in every lane during western blot experiment.
(PDF)

Figure S6 **IPA defined interaction networks associated with the differentially expressed proteins in** ***falciparum*** **malaria.** Differentially expressed serum proteins identified in FM patients were entered as focus molecules in the analytical software to generate biological processes, pathways and molecular networks associated with the identified proteins. (A) The top-scoring network (score 35); cell signaling, molecular transport, vitamin and mineral metabolism. This network incorporated 14 out of the 27 differentially expressed proteins (focus molecules), (B) The second net-work; lipid metabolism, molecular transport, small molecule biochemistry (score 23). This network incorporated 10 focus molecules. Green and red symbols represent proteins that were down and up-regulated in *falciparum* malaria, respectively (identified in this study). White symbols represent associated proteins identified in the functional analysis for which the difference in expression level did not achieve statistical significance in our study.
(PDF)

Figure S7 **Gene Ontology (GO) terms for molecular functions, cellular components and biological processes associated with the differentially expressed serum proteins identified in** ***falciparum*** **malaria.** A total of 1394 Gene Ontology (GO) terms were identified, of which the distribution of second level of GO terms that were enriched in two or more proteins is shown as molecular functions (A) and cellular components (B) and biological processes (C).
(PDF)

Figure S8 **Biological process regulated by differentially expressed serum proteins identified in** ***falciparum*** **and** ***vivax*** **malaria patients.** Regulations were based on Natural Language Processing performed on MEDLINE abstracts as available in GeneSpring software package (version 11.5, Agilent Technologies). Identified process (A) common in both the plasmodial infections (B) specific for *P. falciparum* (C) specific for *P. vivax* infection. Red triangles and blue squares represent positive and negative regulations, respectively.
(PDF)

Figure S9 **Discrimination of** ***falciparum*** **and** ***vivax*** **malaria from healthy controls on the basis of differential expressions of selected serum proteins.** PLS-DA scores plot for (A) FM (red spheres, n = 10) and HC (green spheres, n = 10) samples, based on 5 differentially expressed proteins (Table S8.1A) identified using 2DE, (B) FM (red spheres, n = 6), VM (blue spheres, n = 5) and HC (green spheres, n = 5) samples based on 7 differentially expressed proteins (Table S8.2A) identified using 2D-DIGE. The axes of the plot indicate PLS-DA latent variables. (C & D) Receiver operating characteristic (ROC) curves depicting accuracy of 3 classifier proteins; haptoglobin, apolipoprotein A-I and retinol-binding protein for malaria prediction. The area under the ROC curve (AUC) signifies the accuracy of the different classifier proteins for distinguishing *falciparum* malaria (C) and *vivax* malaria (D) from the healthy controls.
(PDF)

Table S1 **Details of all statistically significant (** ***p*** **<0.05) down-regulated (A) and up-regulated (B) proteins spots in** ***falciparum*** **malaria (compared to healthy controls and** ***vivax*** **malaria).**
(DOC)

Table S2 **Master tables for all MALDI-TOF/TOF identified proteins in** ***falciparum*** **malaria.**
(DOC)

Table S3 **Comparison of differentially expressed serum proteins in** ***falciparum*** **and** ***vivax*** **malaria.**
(DOC)

Table S4 **Comparative serum proteome analysis of malaria (FM and VM) and febrile control (Leptospirosis) by 2D-DIGE.**
(DOC)

Table S5 **Details of the pathways associated with the differentially expressed proteins identified in** ***falciparum*** **malaria defined by IPA, PANTHER and DAVID analysis.**
(DOC)

Table S6 **Gene Ontology (GO) terms for molecular functions, cellular components and biological processes associated with the differentially expressed proteins identified in** ***falciparum*** **malaria.**
(DOC)

Table S7 **Summary of different physiological pathways associated with the differentially expressed proteins identified in** ***falciparum*** **and** ***vivax*** **malaria.**
(DOC)

Table S8 **Prediction models for discrimination of malaria (FM and VM) and controls (HC and FC).**
(DOC)

Table S9 **Receiver operating characteristic (ROC) curve for evaluating performance of different serum proteins on malaria prediction.**
(DOC)

## References

[1] Snow RW, Guerra CA, Noor AM, Myint HY, Hay SI (2005) The global distribution of clinical episodes of Plasmodium falciparum malaria. Nature 434: 214–217. 15759000 10.1038/nature03342 PMC3128492

[2] WHO (2008) WHO World Malaria Report (WHO, Geneva) Available at http://www.who.int/malaria/wmr 2008/malaria2008.pdf. Accessed April 05, 2012.

[3] Price RN, Tjitra E, Guerra CA, Yeung S, White NJ, et al (2007) *Vivax* malaria: neglected and not benign. Am J Trop Med Hyg 77 (6 Suppl). 79–87. PMC2653940 18165478

[4] Olszewski KL, Morrisey JM, Wilinski D, Burns JM, Vaidya AB, et al (2009) Host-parasite interactions revealed by Plasmodium falciparum metabolomics. Cell Host Microbe 5: 191–199. 19218089 10.1016/j.chom.2009.01.004 PMC2737466

[5] Mishra SK, Newton CR (2009) Diagnosis and management of the neurological complications of *falciparum* malaria. Nat Rev Neurol 5: 189–198. 19347024 10.1038/nrneurol.2009.23 PMC2859240

[6] Francischetti IM (2008) Does activation of the blood coagulation cascade have a role in malaria pathogenesis? Trends Parasitol 24: 258–263. 18467176 10.1016/j.pt.2008.03.009 PMC2882796

[7] Acharya P, Pallavi R, Chandran S, Chakravarti H, Middha S, et al (2009) A glimpse into the clinical proteome of human malaria parasites Plasmodium falciparum and Plasmodium vivax. Proteomics Clin appl 3: 1314–1325. 21136953 10.1002/prca.200900090

[8] Crompton PD, Kayala MA, Traore B, Kayentao K, Ongoiba A, et al (2010) A prospective analysis of the Ab response to Plasmodium falciparum before and after a malaria season by protein microarray. Proc Natl Acad Sci U S A 107: 6958–6963. 20351286 10.1073/pnas.1001323107 PMC2872454

[9] Fontaine A, Bourdon S, Belghazi M, Pophillat M, Fourquet P, et al (2012) Plasmodium falciparum infection-induced changes in erythrocyte membrane proteins. Parasitol Res 110: 545–556. 21744020 10.1007/s00436-011-2521-2

[10] Ray S, Reddy PJ, Jain R, Gollapalli K, Moiyadi A, et al (2011) Proteomic technologies for the identification of disease biomarkers in serum: advances and challenges ahead. Proteomics 11: 2139–2161. 21548090 10.1002/pmic.201000460

[11] Albuquerque LM, Trugilho MR, Chapeaurouge A, Jurgilas PB, Bozza PT, et al (2009) Two dimensional difference gel electrophoresis (DiGE) analysis of plasmas from dengue fever patients. J Proteome Res 8: 5431–5441. 19845402 10.1021/pr900236f

[12] Chen JH, Chang YW, Yao CW, Chiueh TS, Huang SC, et al (2004) Plasma proteome of severe acute respiratory syndrome analyzed by two-dimensional gel electrophoresis and mass spectrometry. Proc Natl Acad Sci USA 101: 17039–17044. 15572443 10.1073/pnas.0407992101 PMC535397

[13] Rukmangadachar LA, Kataria J, Hariprasad G, Samantaray JC, Srinivasan A (2011) Two-dimensional difference gel electrophoresis (DIGE) analysis of sera from visceral leishmaniasis patients. Clin Proteomics 8: 4. 21906353 10.1186/1559-0275-8-4 PMC3167202

[14] Srivastava R, Ray S, Vaibhav V, Gollapalli K, Jhaveri T, et al. (2012) Serum profiling of leptospirosis patients to investigate proteomic alterations. J Proteomics. (In press:10.1016/j.jprot.2012.04.007).. 10.1016/j.jprot.2012.04.007 PMC7185557 22554907

[15] Ray S, Kamath KS, Srivastava R, Raghu D, Gollapalli K, et al (2012) Serum proteome analysis of *vivax* malaria: An insight into the disease pathogenesis and host immune response. J Proteomics 75: 3063–3080. 22086083 10.1016/j.jprot.2011.10.018

[16] Bahk YY, Na BK, Cho SH, Kim JY, Lim KJ, et al (2010) Proteomic analysis of Haptoglobin and Amyloid A protein levels in patients with *vivax* malaria. Korean J Parasitol 48: 203–211. 20877498 10.3347/kjp.2010.48.3.203 PMC2945794

[17] Armah HB, Wilson NO, Sarfo BY, Powell MD, Bond VC, et al (2007) Cerebrospinal fluid and serum biomarkers of cerebral malaria mortality in Ghanaian children. Malaria J 6: 147. 10.1186/1475-2875-6-147 PMC2186349 17997848

[18] Kassa FA, Shio MT, Bellemare MJ, Faye B, Ndao M, et al (2011) New inflammation-related biomarkers during malaria infection. PLoS One 6: e26495 doi:10.1371/journal.pone.0026495. 22028888 10.1371/journal.pone.0026495 PMC3197653

[19] Taylor-Robinson AW (2000) Increased production of acute-phase proteins corresponds to the peak parasitaemia of primary malaria infection. Parasitol Int 48: 297–301. 10725693 10.1016/s1383-5769(99)00029-x

[20] Wenisch C, Spitzauer S, Florris-Linau K, Rumpold H, Vannaphan S, et al (1997) Complement activation in severe Plasmodium falciparum malaria. Clin Immunol Immunopathol. 85: 166–171. 10.1006/clin.1997.4417 9344699

[21] Rougemont A, Bouvier M, Perrin L, Yerly S, Brenner E, et al (1988) Hypohaptoglobinaemia as an epidemiological and clinical indicator for malaria. Results of two studies in a hyperendemic region in West Africa. Lancet 2: 709–712. 2901568 10.1016/s0140-6736(88)90186-9

[22] Pied S, Tabone MD, Chatellier G, Marussig M, Jardel C, et al (1995) Non specific resistance against malaria pre-erythrocytic stages: involvement of acute phase proteins. Parasite 2: 263–268. 8520801 10.1051/parasite/1995023263

[23] Steel DM, Whitehead AS (1994) The major acute phase reactants: C-reactive protein, serum amyloid P component and serum amyloid A protein. Immunol Today 15: 81–88. 8155266 10.1016/0167-5699(94)90138-4

[24] Kappelle PJ, Bijzet J, Hazenberg BP, Dullaart RP (2011) Lower serum paraoxonase-1 activity is related to higher serum amyloid A levels in metabolic syndrome. Arch Med Res 42: 219–225. 21722818 10.1016/j.arcmed.2011.05.002

[25] Pabon A, Carmona J, Burgos LC, Blair S (2003) Oxidative stress in patients with non-complicated malaria. Clin Biochem 36: 71–78. 12554064 10.1016/s0009-9120(02)00423-x

[26] Delanghe JR, Langlois MR (2001) Hemopexin: a review of biological aspects and the role in laboratory medicine. Clin Chim Acta 312: 13–23. 11580905 10.1016/s0009-8981(01)00586-1

[27] Yerly S, Bouvier M, Rougemont A, Srivastava I, Perrin LH (1990) Development of a haptoglobin ELISA. Its use as an indicator for malaria. Acta Trop 47: 237–244. 1973025 10.1016/0001-706x(90)90015-r

[28] Harrison T, Samuel BU, Akompong T, Hamm H, Mohandas N, et al (2003) Erythrocyte G protein-coupled receptor signaling in malarial infection. Science 301: 1734–1736. 14500986 10.1126/science.1089324

[29] Wozniak MA, Faragher EB, Todd JA, Koram KA, Riley EM, et al (2003) Does apolipoprotein E polymorphism influence susceptibility to malaria? J Med Genet 40: 348–351. 12746397 10.1136/jmg.40.5.348 PMC1735474

[30] Verhoeckx KC, Gaspari M, Bijlsma S, van der Greef J, Witkamp RF, et al (2005) In search of secreted protein biomarkers for the anti-inflammatory effect of ß_2_-adrenergic receptor agonists: application of DIGE technology in combination with multivariate and univariate data analysis tools. J Proteome Res 4: 2015–2023. 16335946 10.1021/pr050183u

[31] Pérez-Enciso M, Tenenhaus M (2003) Prediction of clinical outcome with microarray data: a partial least squares discriminant analysis (PLS-DA) approach. Hum Genet 112: 581–592. 12607117 10.1007/s00439-003-0921-9

[32] Omenn GS (2004) Advancement of biomarker discovery and validation through the HUPO plasma proteome project. Dis Markers 20: 131–134. 15502245 10.1155/2004/579363 PMC3839274

[33] Payne D (1988) Use and limitations of light microscopy for diagnosing malaria at the primary health care level. Bull World Health Organ 66: 621–626. 2463112 PMC2491188

[34] Mockenhaupt FP, Rong B, Till H, Eggelte TA, Beck S, et al (2000) Submicroscopic Plasmodium falciparum infections in pregnancy in Ghana. Trop Med Int Health 5: 167–173. 10747278 10.1046/j.1365-3156.2000.00532.x

[35] WHO (2008) World Health Organization. List of known commercially available antigen-detecting malaria RDTs. Available at: http://www.wpro.who.int/sites/rdt. Accessed April 05, 2012..

[36] Bell D, Peeling RW (2006) WHO-Regional Office for the Western Pacific/TDR. Evaluation of rapid diagnostic tests: malaria. Nat Rev Microbiol 4 (9 Suppl). S34–38. 10.1038/nrmicro1524 17034070

[37] Chen YY, Lin SY, Yeh YY, Hsiao HH, Wu CY, et al (2005) A modified protein precipitation procedure for efficient removal of albumin from serum. Electrophoresis 26: 2117–2127. 15880626 10.1002/elps.200410381

[38] Benjamini Y, Hochberg Y (2000) On the adaptive control of the false discovery rate in multiple testing with independent statistics. J Educ Behav Stat 25: 60–83.

[39] Shevchenko A, Tomas H, Havlis J, Olsen JV, Mann M (2006) In-gel digestion for mass spectrometric characterization of proteins and proteomes. Nat Protoc 1: 2856–2860. 17406544 10.1038/nprot.2006.468

[40] Gollapalli K, Ray S, Srivastava R, Renu D, Singh P, et al. (2012) Investigation of serum proteome alterations in human glioblastoma multiforme. Proteomics. (In press: DOI 10.1002/pmic.201200002).. 10.1002/pmic.201200002 22684992

[41] Thomas PD, Kejariwal A, Guo N, Mi H, Campbell MJ, et al (2006) Applications for protein sequence-function evolution data: mRNA/protein expression analysis and coding SNP scoring tools. Nucleic Acids Res 34: W645–50. 16912992 10.1093/nar/gkl229 PMC1538848

[42] Huang da W, Sherman BT, Lempicki RA (2009) Systematic and integrative analysis of large gene lists using DAVID bioinformatics resources. Nat Protoc 4: 44–57. 19131956 10.1038/nprot.2008.211

[43] Huang da W, Sherman BT, Lempicki RA (2009) Bioinformatics enrichment tools: paths toward the comprehensive functional analysis of large gene lists. Nucleic Acids Res 37: 1–13. 19033363 10.1093/nar/gkn923 PMC2615629

[44] Boulesteix AL, Strimmer K (2007) Partial least squares: a versatile tool for the analysis of high-dimensional genomic data. Brief Bioinform 8: 32–44. 16772269 10.1093/bib/bbl016

[45] Barker M, Rayens W (2003) Partial least squares for discrimination. J Chemom 17: 166–73.

[46] Wold S, Esbensen K, Geladi P (1987) Principal component analysis. Chem Intell Lab System 2: 37–52.

[47] Burges CJC (1998) A tutorial on support vector machines for pattern recognition. Data Min Knowl Discov 2: 121–167.

