# Supplementary figures and images for: Proteomic Investigation of Falciparum and Vivax Malaria for Identification of Surrogate Protein Markers
Source: PLoS One. 2012 Aug 9;7(8):e41751. doi: 10.1371/journal.pone.0041751 (PMC3415403; doi:10.1371/journal.pone.0041751)

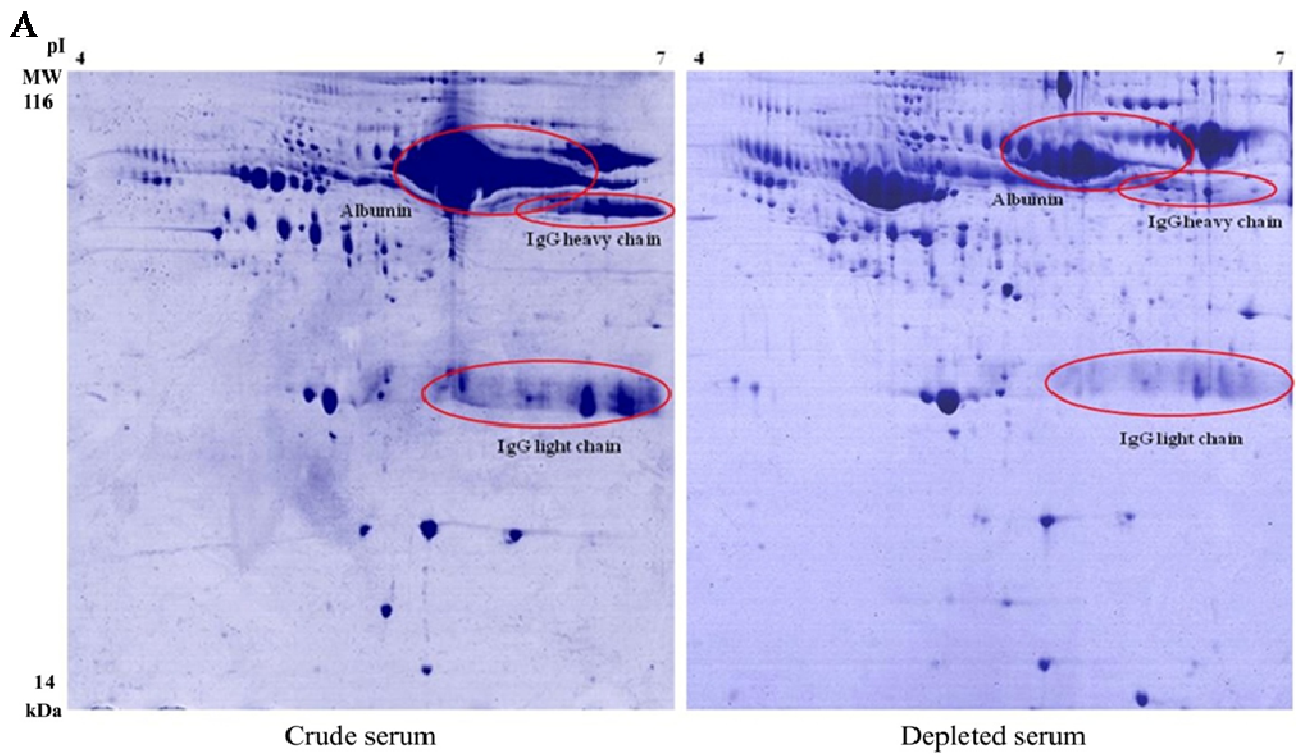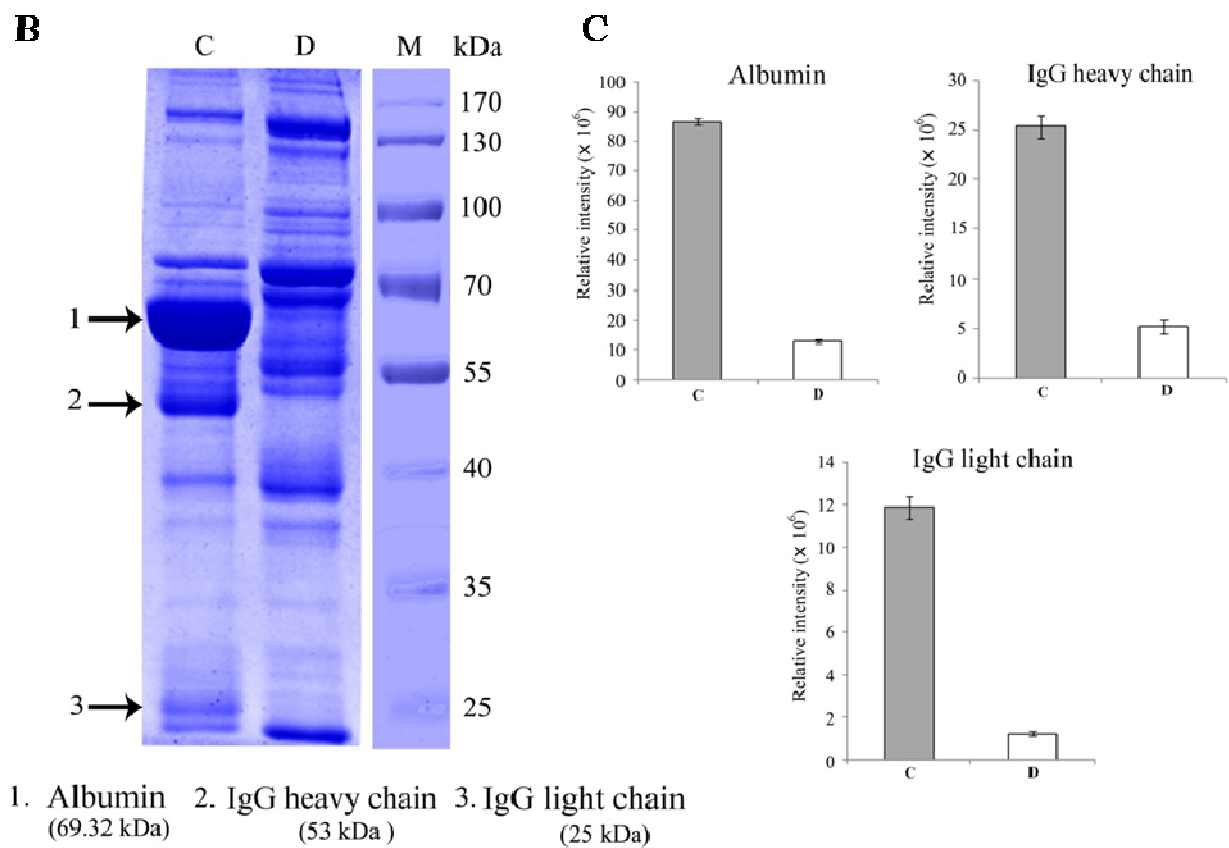

**Figure S1**

Supplement: Figure S1 — Evaluation of the depletion efficiency for albumin and IgG from human serum. Two major high-abundance serum proteins; albumin and IgG were removed using Albumin & IgG Depletion SpinTrap (GE Healthcare) to reduce the dynamic range of serum protein concentration. (A) Levels of albumin and IgG in CBB stained 2D gel before and after depletion. 600 µg of total serum proteins were focused on linear pH 4–7 IPG strips (18 cm) and then separated on 12.5% polyacrylamide gels. Depletion of the top two high-abundance proteins (albumin and IgG) introduced nearly two-fold increase in overall spot number in 2D gels. (B) Levels of albumin and IgG in CBB stained 1D-SDS-PAGE gel before and after depletion showing the efficiency of the depletion process. 10 µg of total crude [C] and depleted [D] serum proteins were loaded onto each lane and separated on 10% polyacrylamide gels. (C) Densitometric analysis of the 1D-SDS-PAGE gels revealed around 85% and 80% depletion of albumin and IgG respectively. (PDF) [file pone.0041751.s001.pdf]

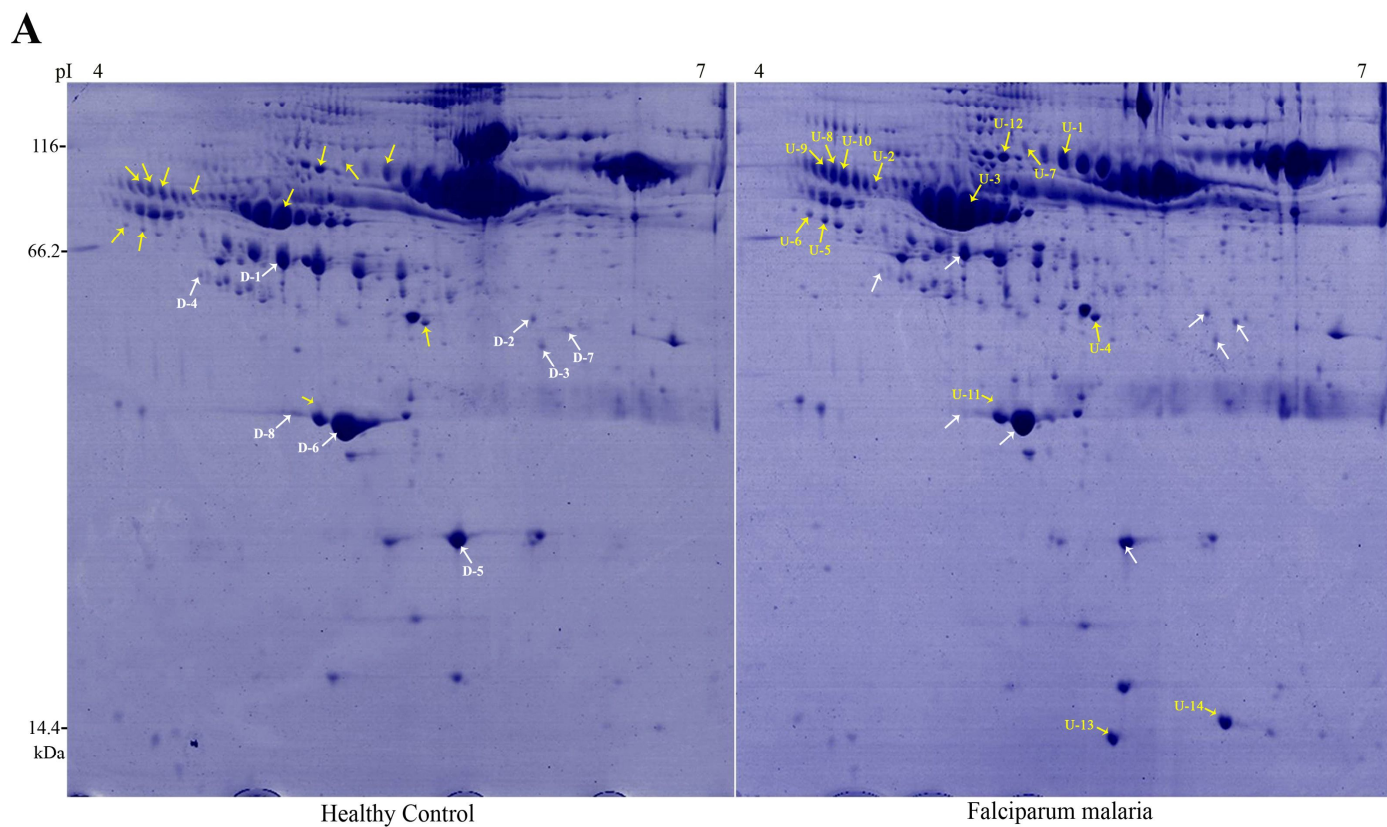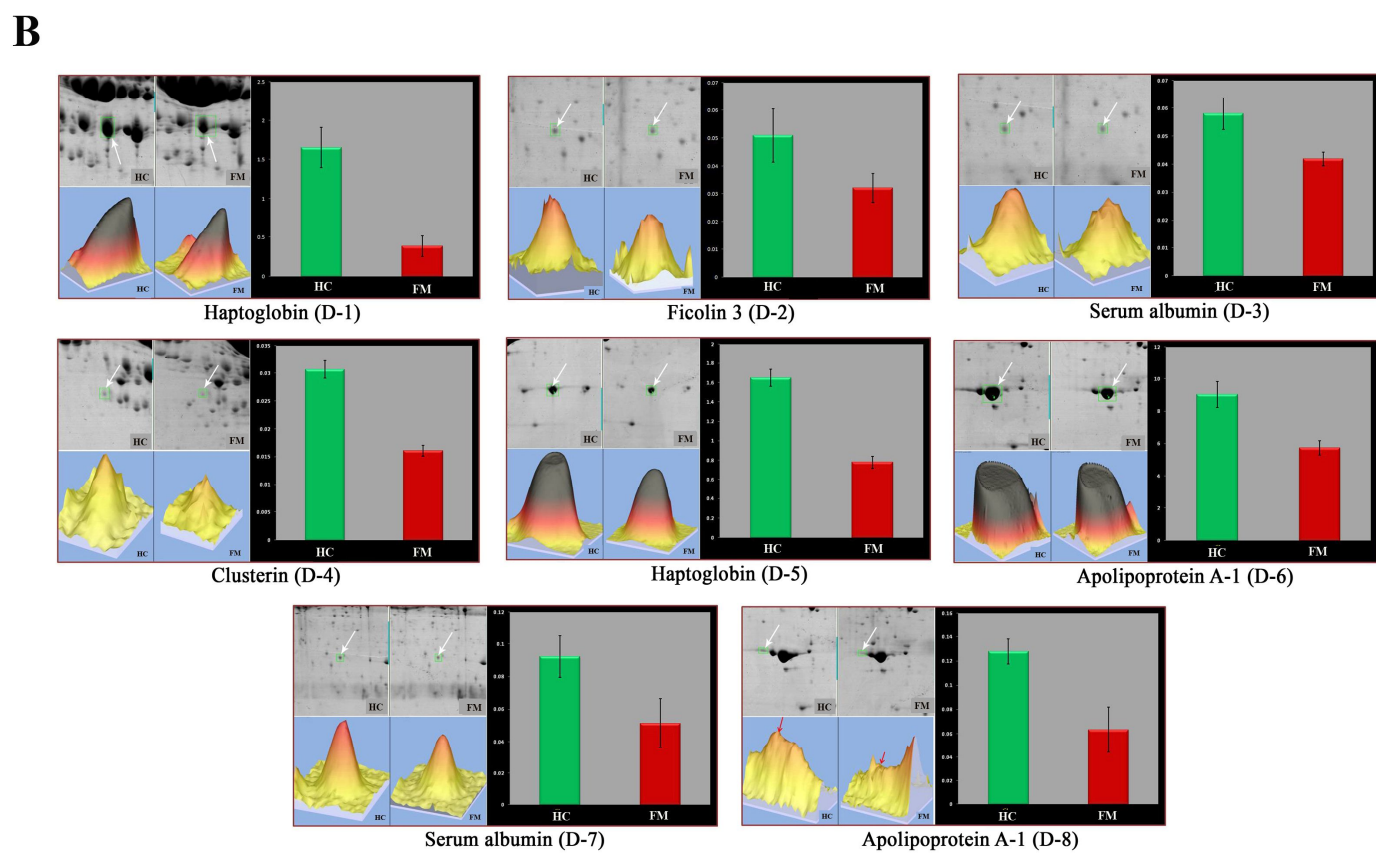

C

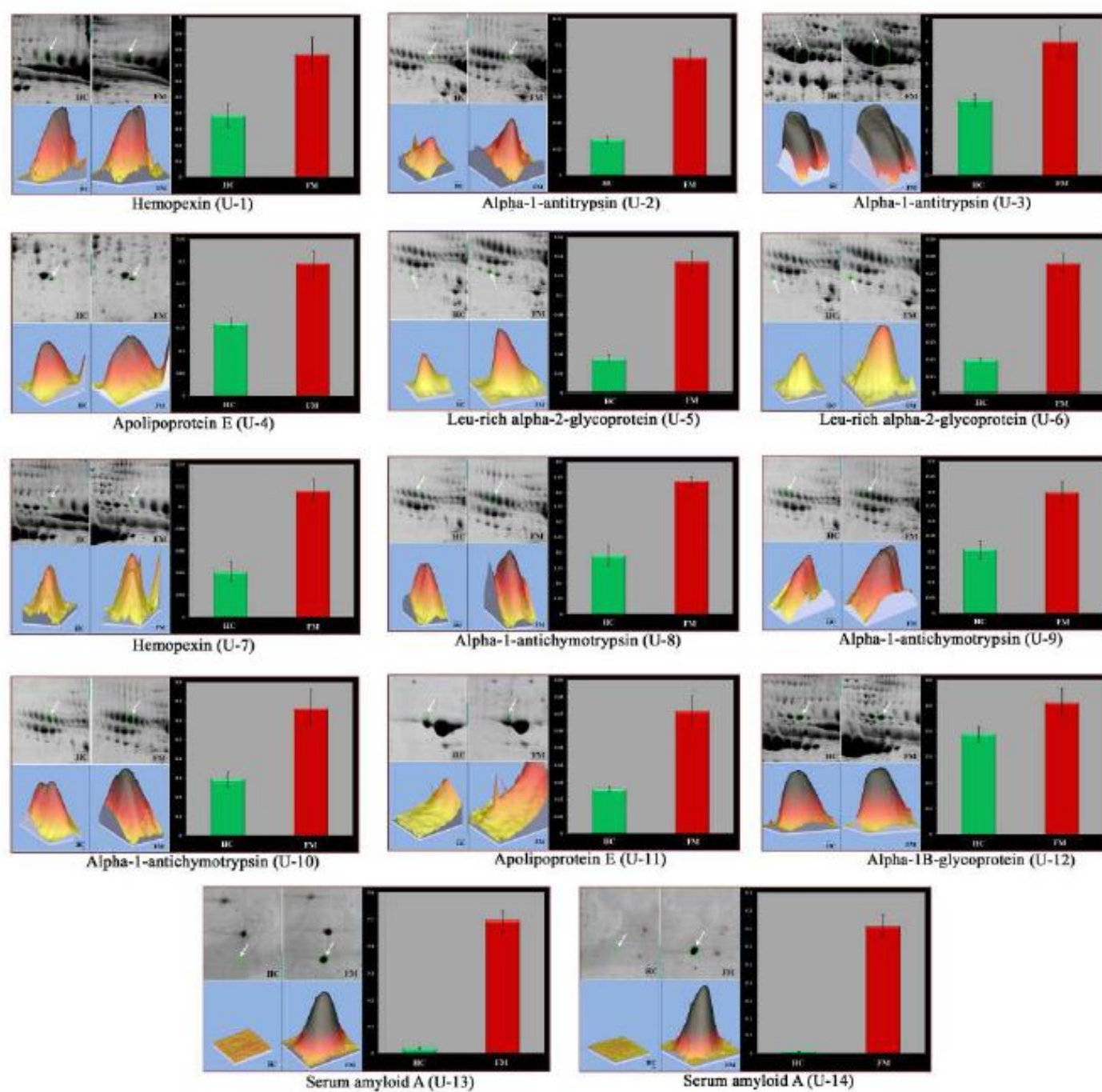

Figure S2

Supplement: Figure S2 — Trends of differentially expressed proteins in falciparum malaria patients visualized in 2DE gels. (A) Representative 2D gels of serum from healthy controls and FM patients. 600 µg of total serum proteins were focused on linear pH 4–7 IPG strips (18 cm) and then separated on 12.5% polyacrylamide gels, which were stained with Gel Code Blue Stain. Protein spots exhibiting significantly altered expression levels are marked on the gels. Down (B) and up (C) -regulation of protein expression levels in FM patients. The 3D images of statistically significant (p<0.05) differentially expressed spots were analyzed using IMP7 software. Data is represented as mean ± SEM (where n = 20). (PDF) [file pone.0041751.s002.pdf]

**A** pI 4

7

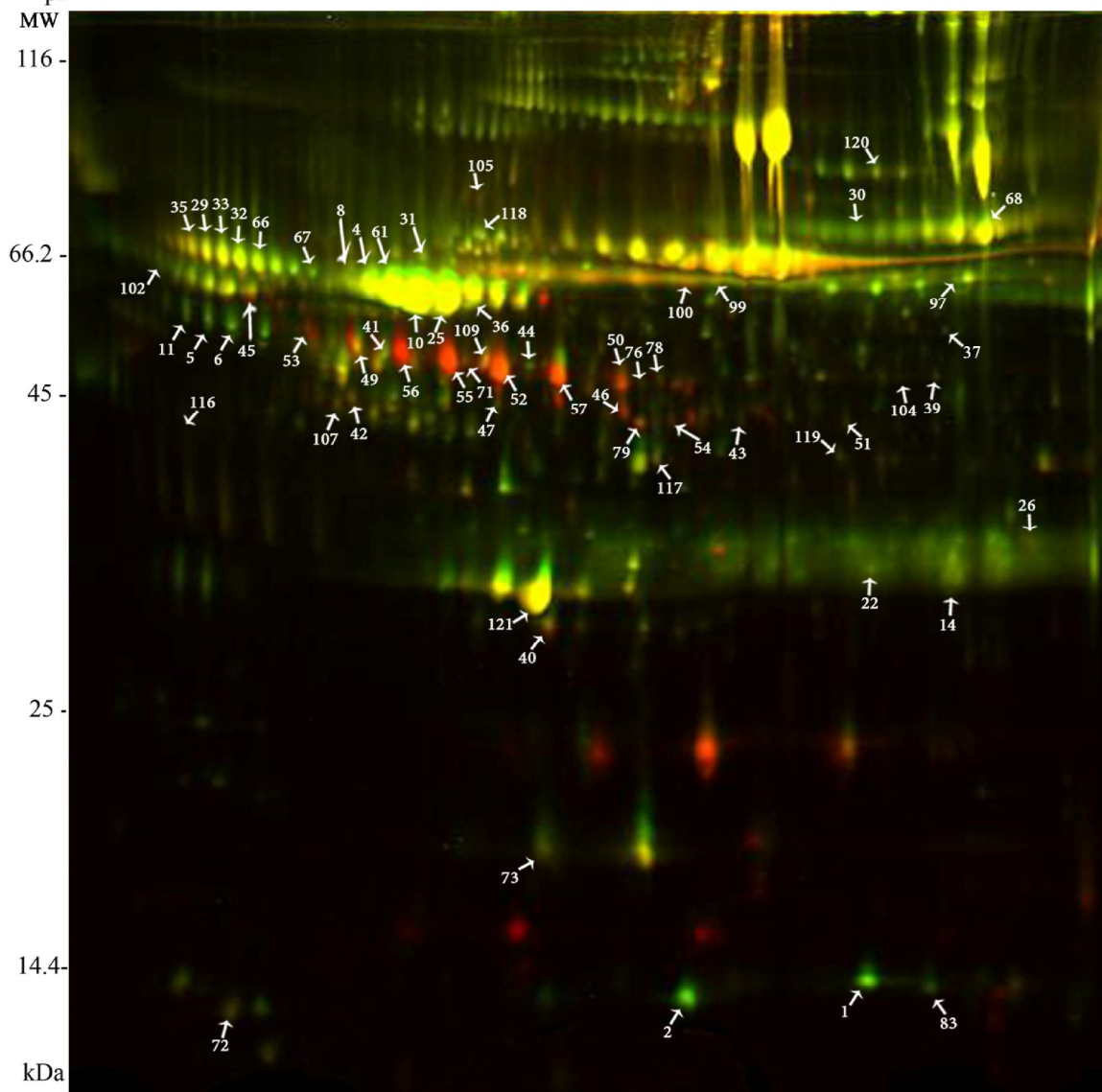

**B**

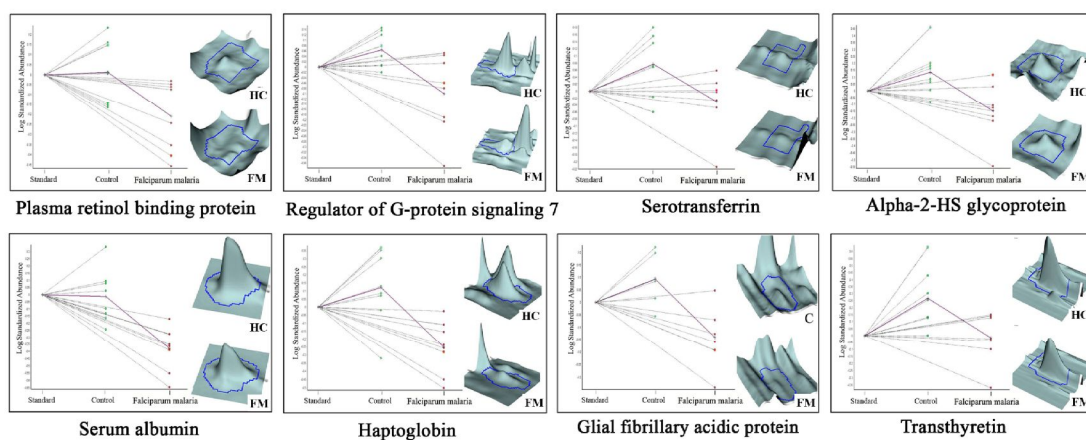

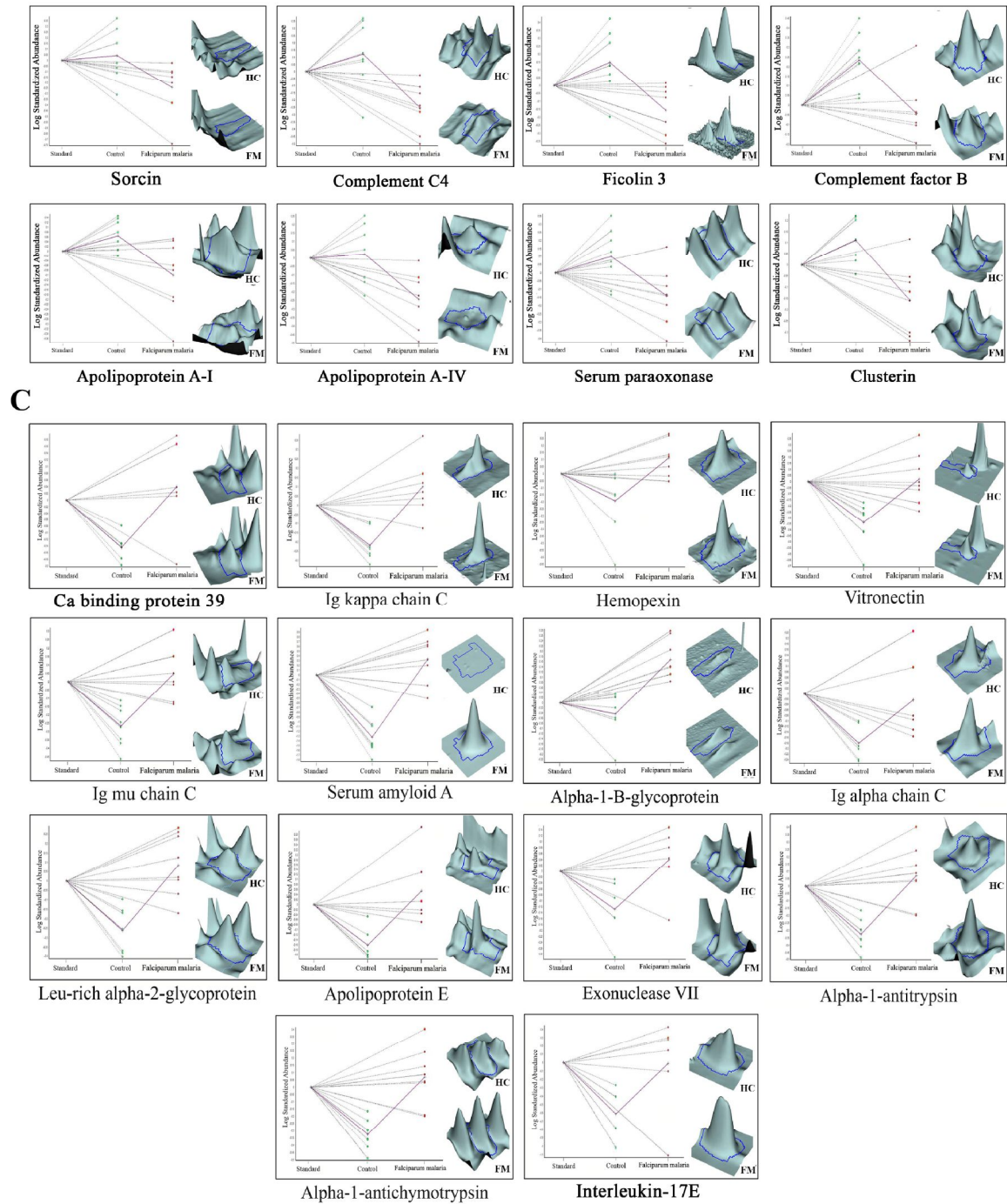

**Figure S3**

Supplement: Figure S3 — Trends of differentially expressed proteins in falciparum malaria patients visualized in 2D-DIGE gels. (A) Representative 2D-DIGE image comparing the serum proteome of healthy subjects and FM patients. Differentially expressed protein spots identified in FM are marked on the gel. Graphical and 3D fluorescence intensity representations of statistically significant, MS identified protein spots down (B) or up-regulated (C) in FM patients (p<0.05) obtained in biological variation analysis (BVA) using DeCyder 2D software. Graphs showing the decrease/increase in the standardized log abundance of spot intensity in the FM cohort of the study. (PDF) [file pone.0041751.s003.pdf]

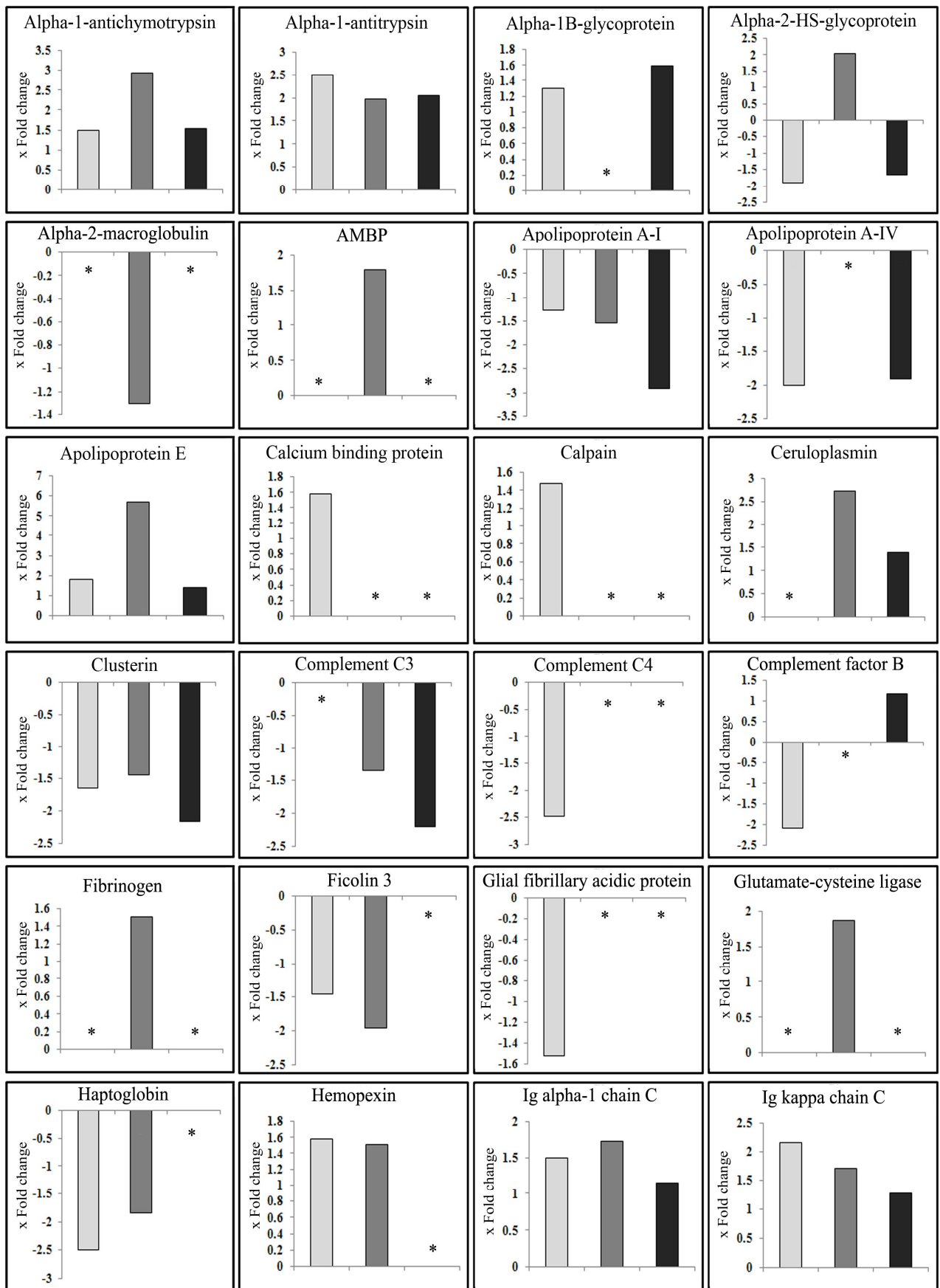

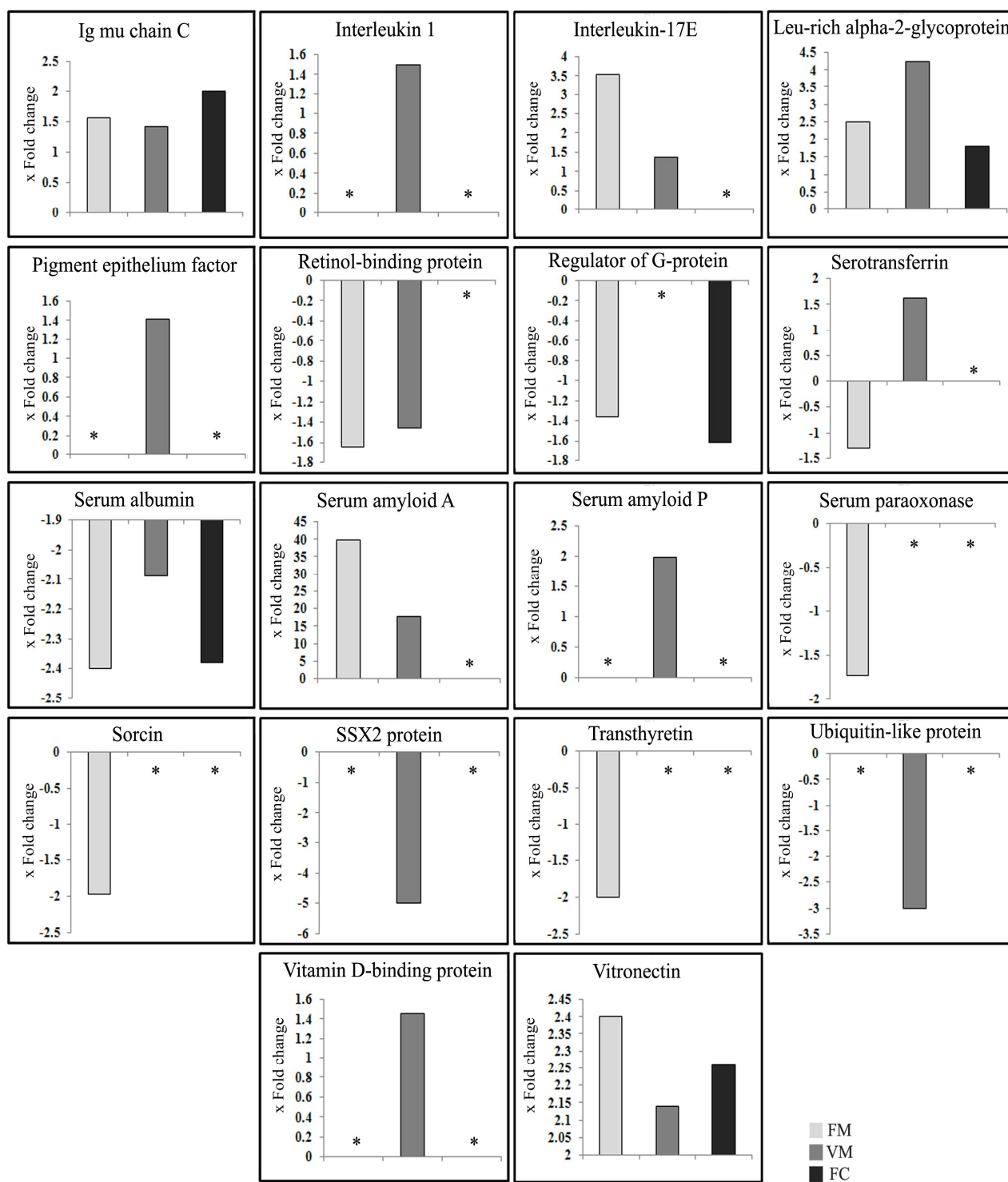

**Figure S4**

Supplement: Figure S4 — Comparative serum proteome analysis of falciparum malaria (FM), vivax malaria (VM) and febrile control (Leptospirosis). Bar-diagrams showing altered expression levels of different serum proteins in malaria (FM and VM) and febrile control (Leptospirosis patients). * Indicates “no differential expression” (alterations in expression level not statistically significant). Alterations in protein expression levels in malaria and leptospirosis patients were determined using healthy subjects as controls. Fold change values were calculated by keeping the expression level of the proteins (mean value) in healthy population as baseline. (PDF) [file pone.0041751.s004.pdf]

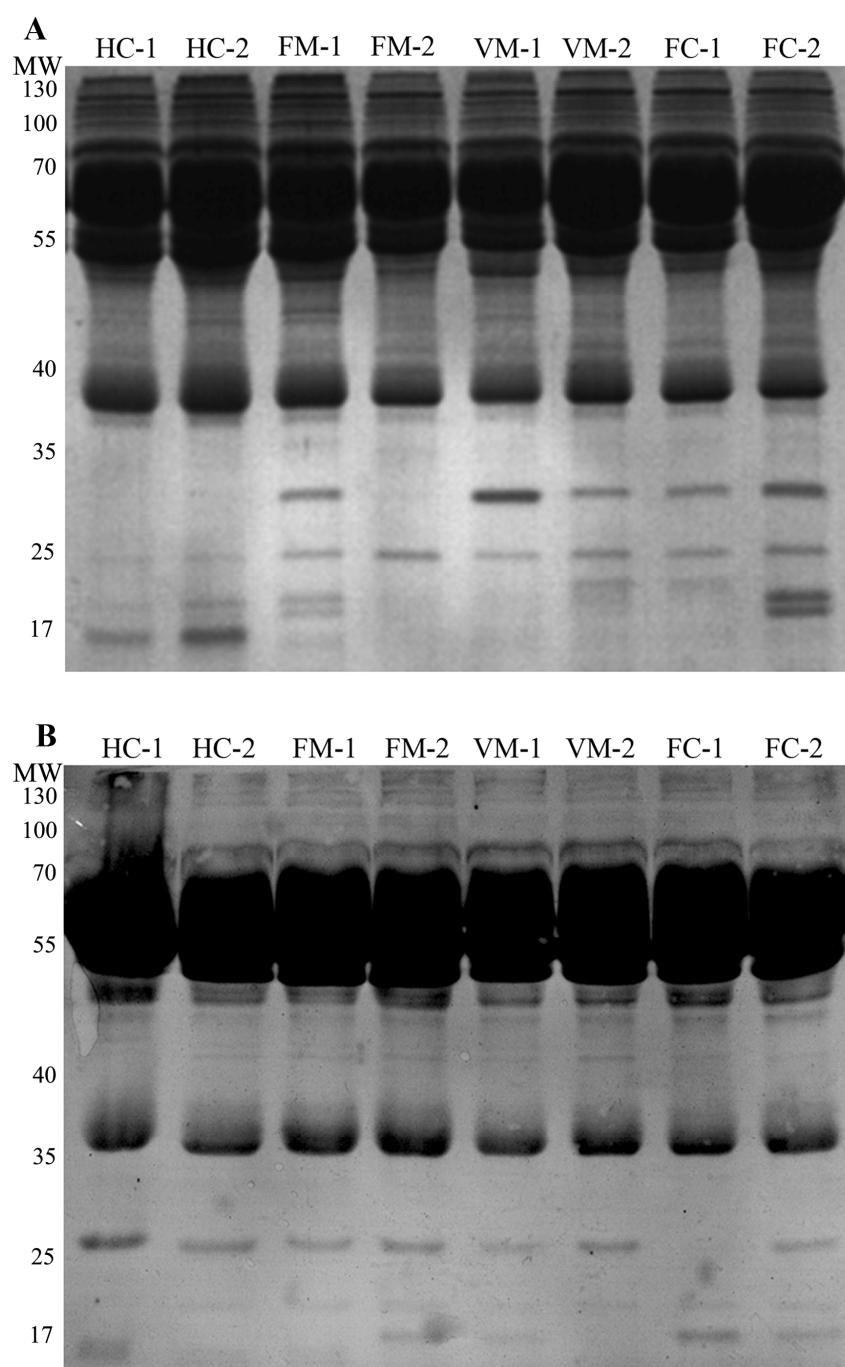

**Figure S5**

Supplement: Figure S5 — Equal loading of protein samples during western blot experiment. Representative CBB stained SDS-PAGE gel (A) and Ponceau stained blot (B) containing the resolved proteins depicting equal loading (50 µg) of the samples [malaria patients (FM and VM) and controls (FC and HC)] in every lane during western blot experiment. (PDF) [file pone.0041751.s005.pdf]

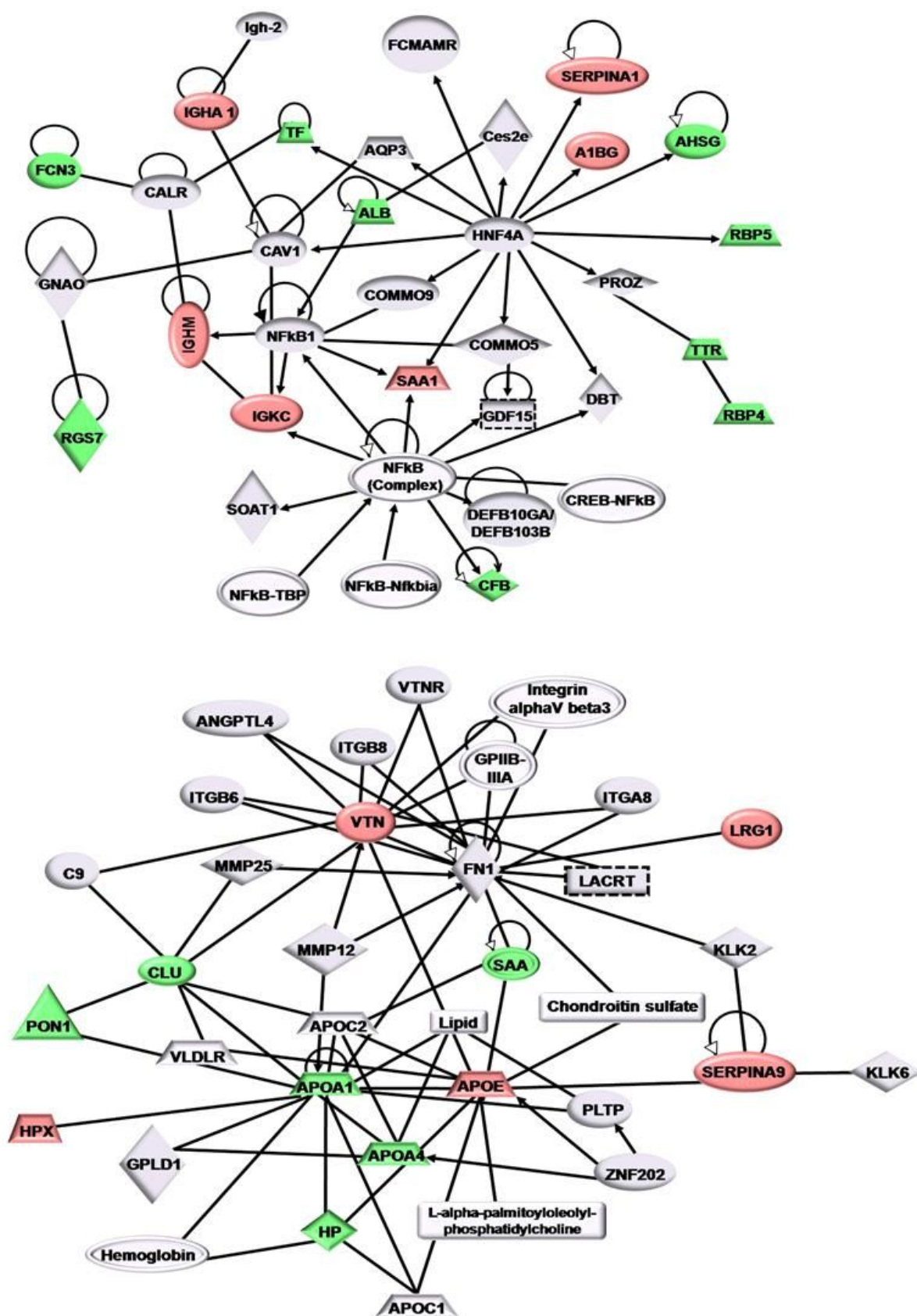

Figure S6

Supplement: Figure S6 — IPA defined interaction networks associated with the differentially expressed proteins in falciparum malaria. Differentially expressed serum proteins identified in FM patients were entered as focus molecules in the analytical software to generate biological processes, pathways and molecular networks associated with the identified proteins. (A) The top-scoring network (score 35); cell signaling, molecular transport, vitamin and mineral metabolism. This network incorporated 14 out of the 27 differentially expressed proteins (focus molecules), (B) The second net-work; lipid metabolism, molecular transport, small molecule biochemistry (score 23). This network incorporated 10 focus molecules. Green and red symbols represent proteins that were down and up-regulated in falciparum malaria, respectively (identified in this study). White symbols represent associated proteins identified in the functional analysis for which the difference in expression level did not achieve statistical significance in our study. (PDF) [file pone.0041751.s006.pdf]

**A**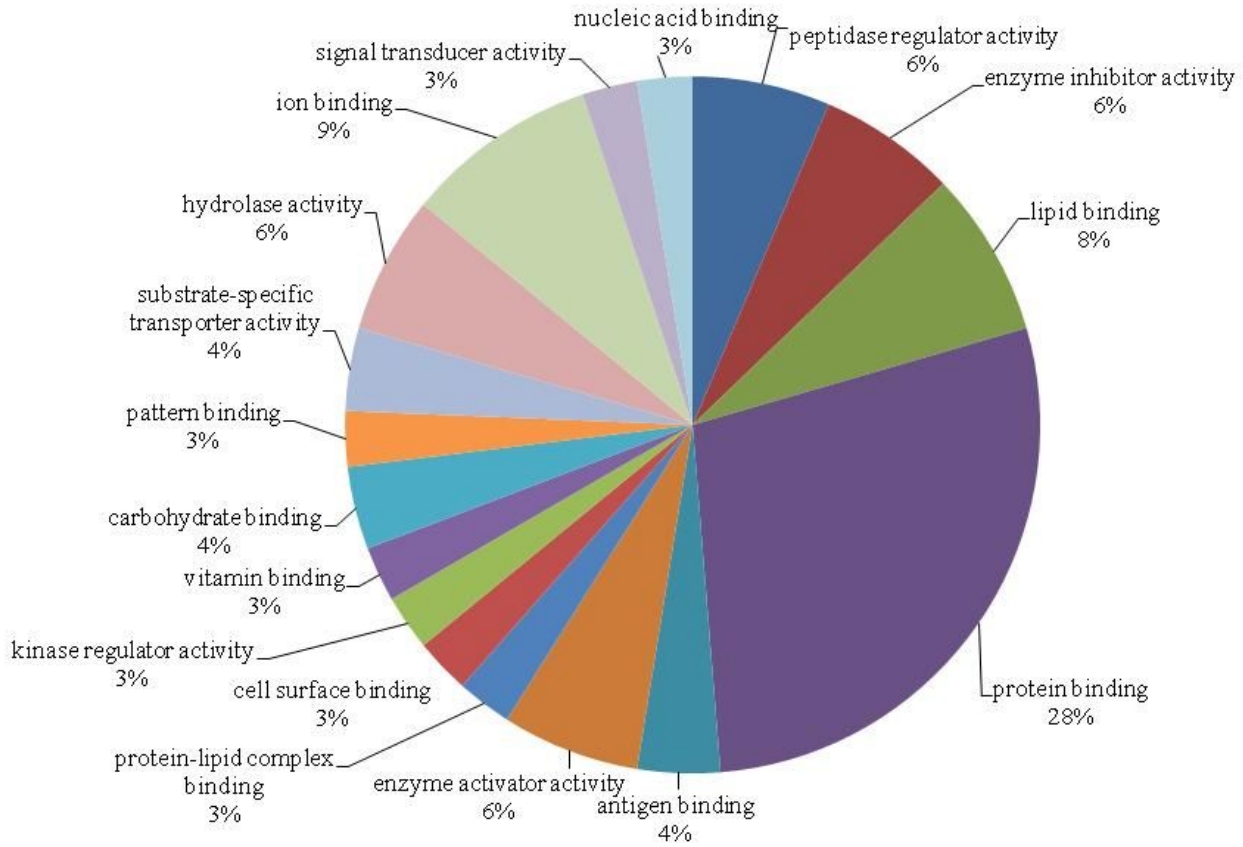**B**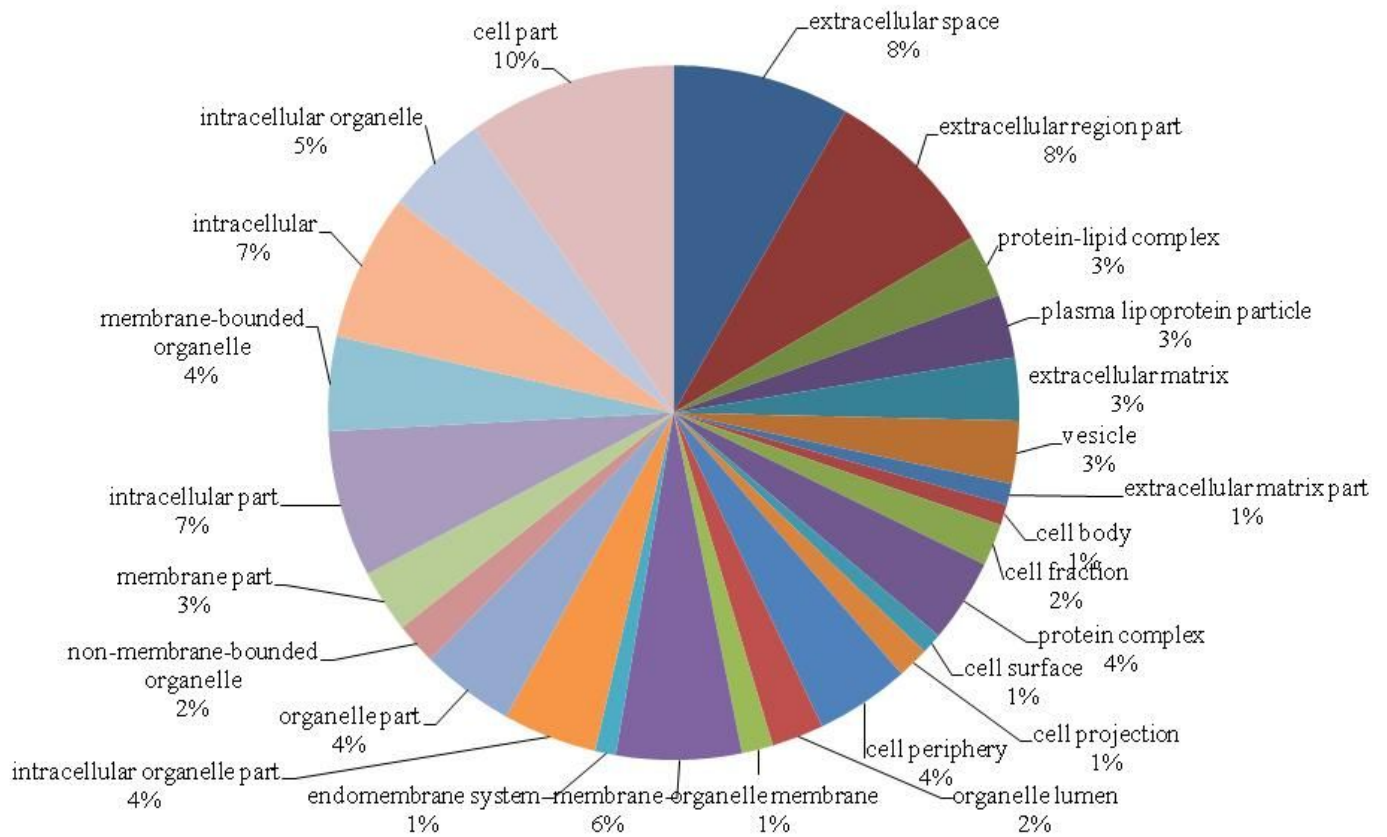

C

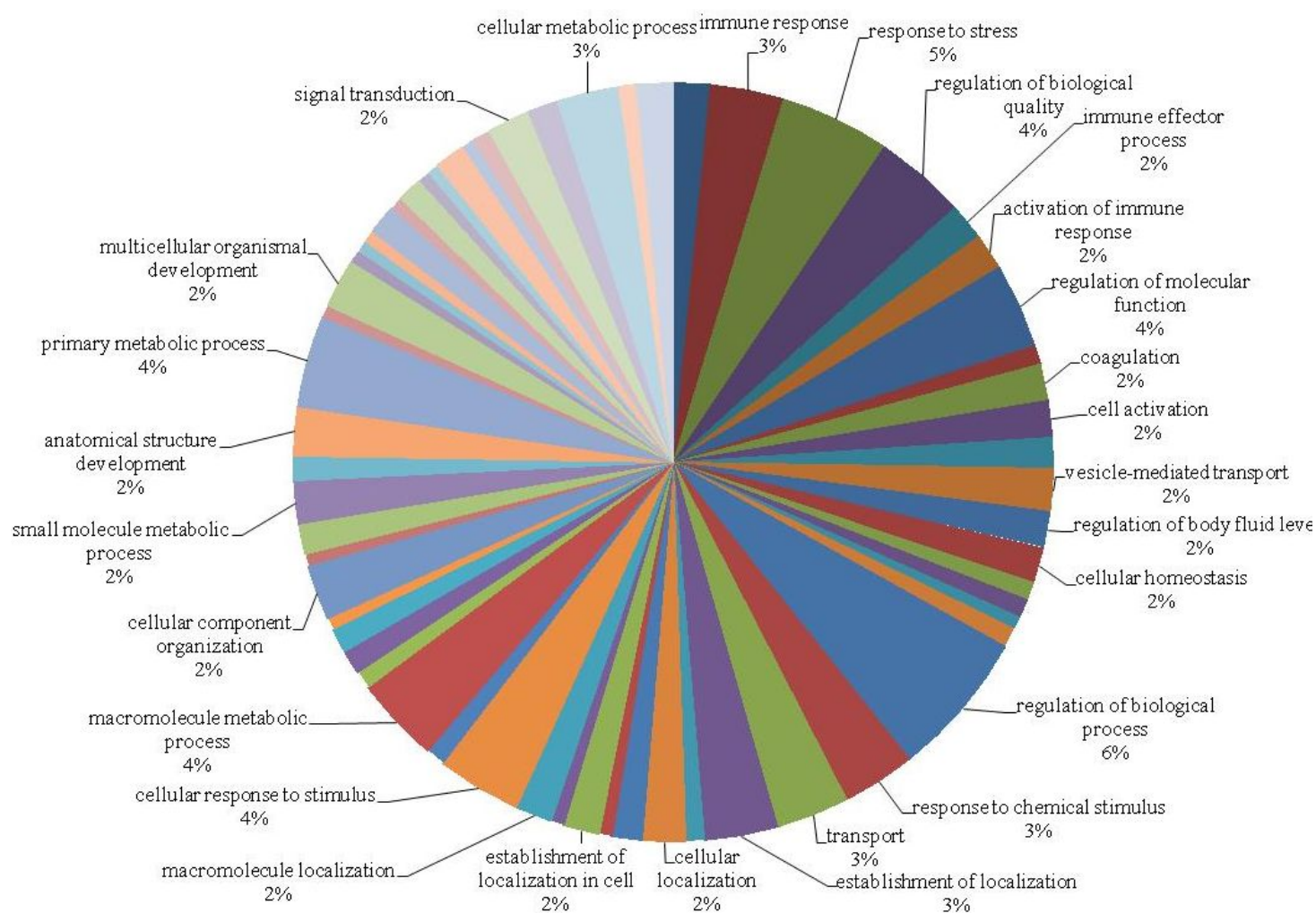

Figure S7

Supplement: Figure S7 — Gene Ontology (GO) terms for molecular functions, cellular components and biological processes associated with the differentially expressed serum proteins identified in falciparum malaria. A total of 1394 Gene Ontology (GO) terms were identified, of which the distribution of second level of GO terms that were enriched in two or more proteins is shown as molecular functions (A) and cellular components (B) and biological processes (C). (PDF) [file pone.0041751.s007.pdf]

# A

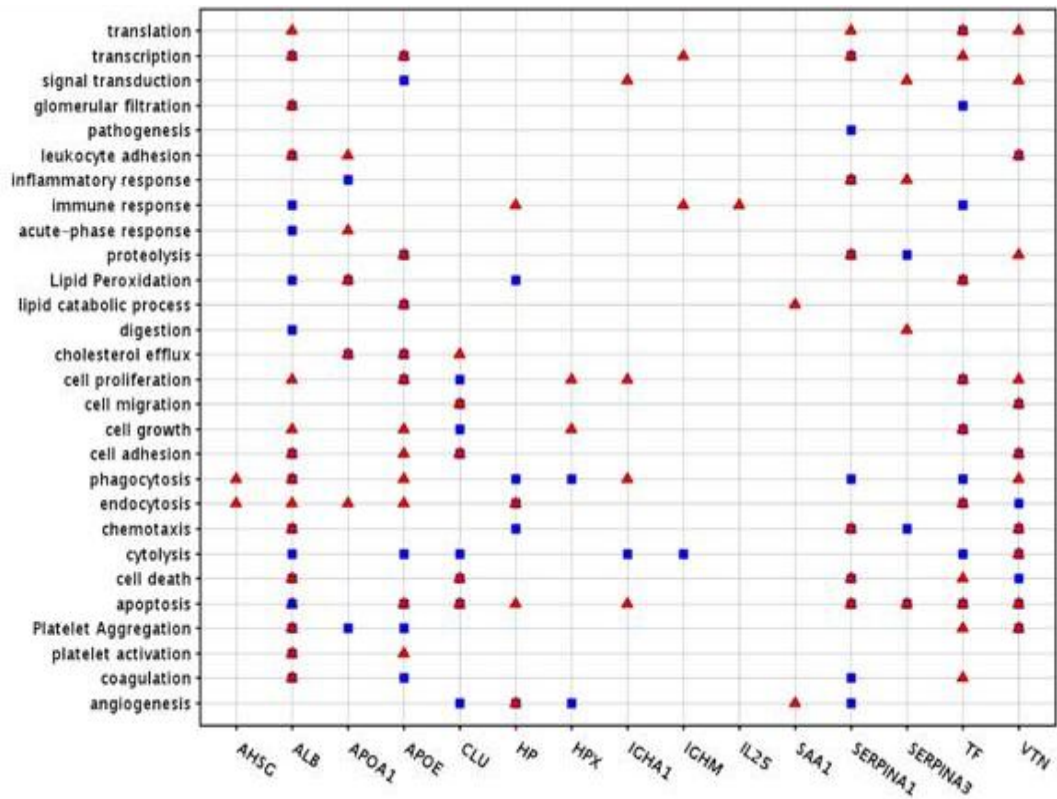

# B

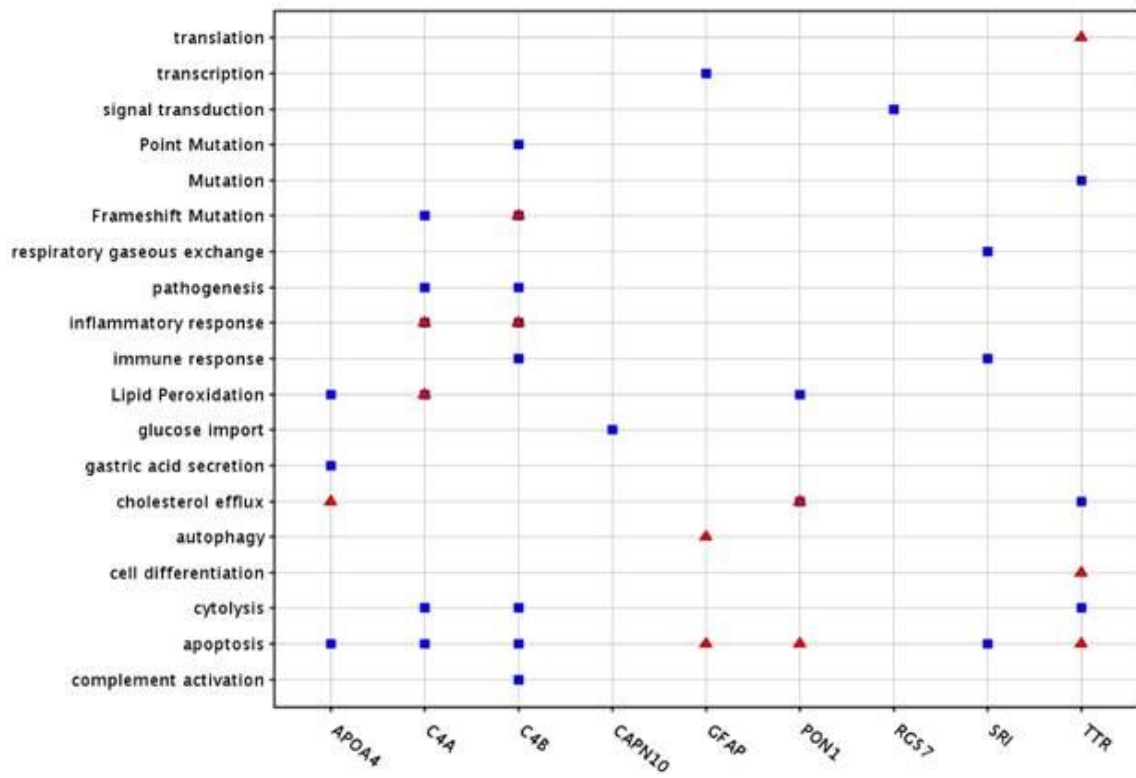

C

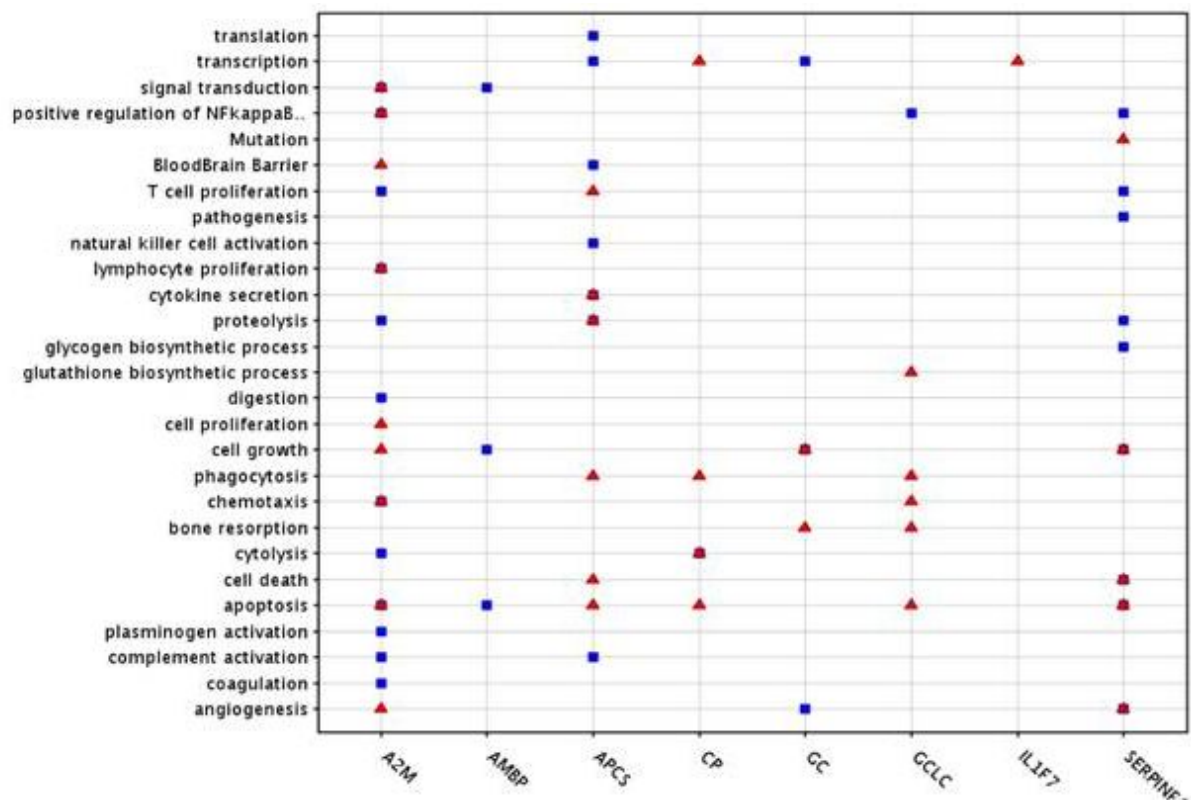

Figure S8

Supplement: Figure S8 — Biological process regulated by differentially expressed serum proteins identified in falciparum and vivax malaria patients. Regulations were based on Natural Language Processing performed on MEDLINE abstracts as available in GeneSpring software package (version 11.5, Agilent Technologies). Identified process (A) common in both the plasmodial infections (B) specific for P. falciparum (C) specific for P. vivax infection. Red triangles and blue squares represent positive and negative regulations, respectively. (PDF) [file pone.0041751.s008.pdf]

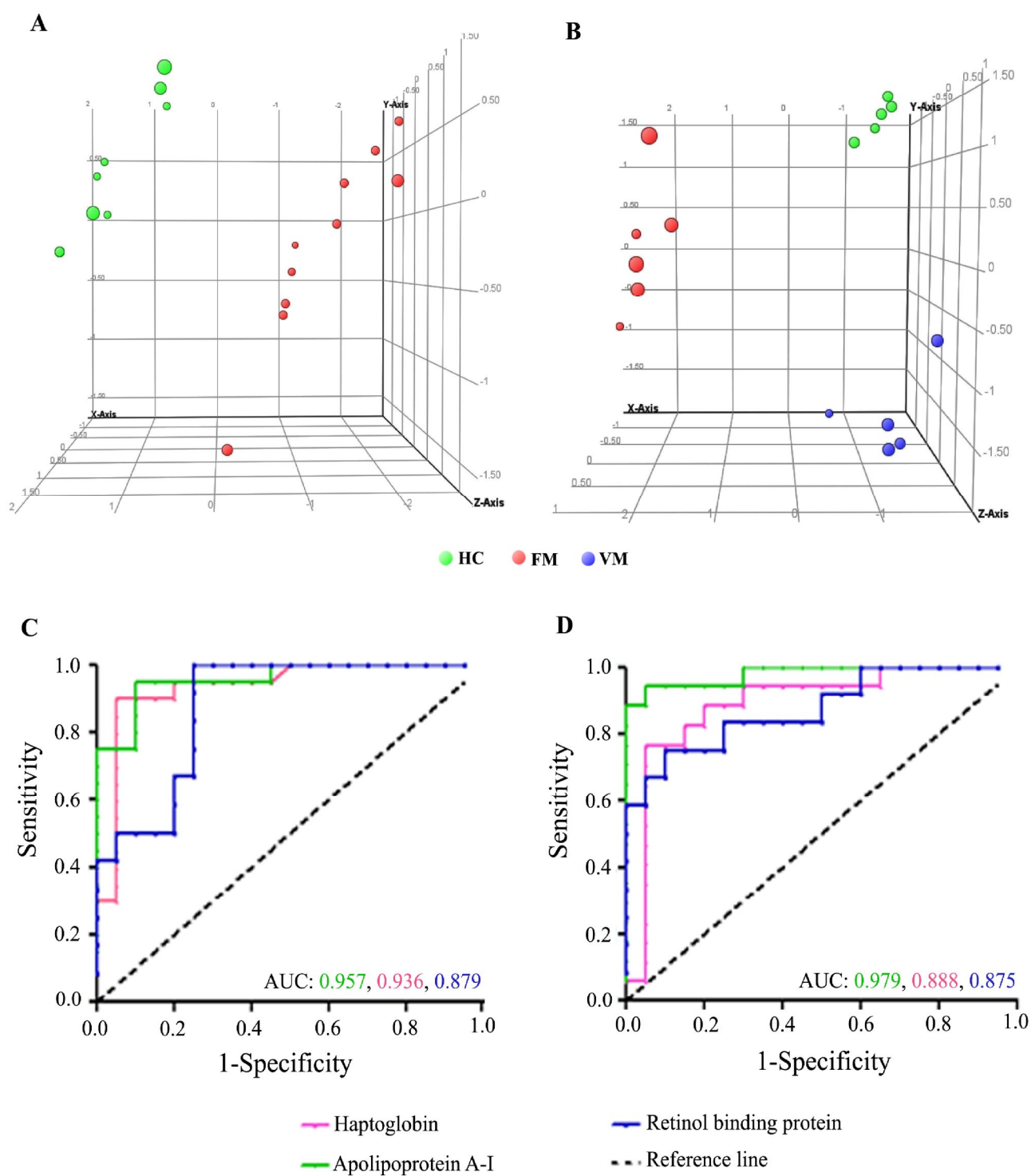

**Figure S9**

Supplement: Figure S9 — Discrimination of falciparum and vivax malaria from healthy controls on the basis of differential expressions of selected serum proteins. PLS-DA scores plot for (A) FM (red spheres, n = 10) and HC (green spheres, n = 10) samples, based on 5 differentially expressed proteins (Table S8.1A) identified using 2DE, (B) FM (red spheres, n = 6), VM (blue spheres, n = 5) and HC (green spheres, n = 5) samples based on 7 differentially expressed proteins (Table S8.2A) identified using 2D-DIGE. The axes of the plot indicate PLS-DA latent variables. (C & D) Receiver operating characteristic (ROC) curves depicting accuracy of 3 classifier proteins; haptoglobin, apolipoprotein A-I and retinol-binding protein for malaria prediction. The area under the ROC curve (AUC) signifies the accuracy of the different classifier proteins for distinguishing falciparum malaria (C) and vivax malaria (D) from the healthy controls. (PDF) [file pone.0041751.s009.pdf]
