# Supplementary material for: Proteomic Investigation of Falciparum and Vivax Malaria for Identification of Surrogate Protein Markers
Source: PLoS One. 2012 Aug 9;7(8):e41751. doi: 10.1371/journal.pone.0041751 (PMC3415403; doi:10.1371/journal.pone.0041751)
Supplement: Table S1 — Details of all statistically significant ( p <0.05) down-regulated (A) and up-regulated (B) proteins spots in falciparum malaria (compared to healthy controls and vivax malaria). (DOC) [file pone.0041751.s010.doc]

**Table S1.**  Details of all statistically significant (*p* < 0.05) down-regulated (A) and up-regulated (B) proteins spots in *falciparum* malaria (compared to healthy controls and *vivax* malaria)

| **A. Down-regulated spots** | | | | | | |
| --- | --- | --- | --- | --- | --- | --- |
| **Protein ID** | ***p* value**  **(t-test)** | **Average HC** | **SE HC** | **Average FM** | **SE FM** | **Average ratio** |
| D-1 | 0.01198 | 1.65173 | 0.25757 | 0.38571 | 0.13157 | -4.28 |
| D-2 | 0.03411 | 0.05092 | 0.00953 | 0.03217 | 0.00516 | -1.58 |
| D-3 | 0.03792 | 0.05804 | 0.00569 | 0.04183 | 0.00247 | -1.39 |
| D-4 | 0.02922 | 0.03066 | 0.00161 | 0.01604 | 0.00097 | -1.91 |
| D-5 | 0.00999 | 1.64743 | 0.08701 | 0.77779 | 0.06431 | -2.12 |
| D-6 | 0.01695 | 9.03067 | 0.79929 | 5.73896 | 0.44819 | -1.58 |
| D-7 | 0.03682 | 0.09232 | 0.01271 | 0.0513 | 0.01512 | -1.80 |
| D-8 | 0.04767 | 0.1277 | 0.0103 | 0.06315 | 0.01893 | -2.02 |

| **B. Up-regulated spots** | | | | | | |
| --- | --- | --- | --- | --- | --- | --- |
| **Protein ID** | ***p* value**  **(t-test)** | **Average HC** | **SE HC** | **Average FM** | **SE FM** | **Average ratio** |
| U-1 | 0.03728 | 0.38323 | 0.07469 | 0.76878 | 0.10781 | 2.00 |
| U-2 | 0.00464 | 0.02748 | 0.00263 | 0.09017 | 0.00647 | 3.28 |
| U-3 | 0.02437 | 3.3563 | 0.28557 | 5.97203 | 0.68015 | 1.78 |
| U-4 | 0.02043 | 0.16107 | 0.01081 | 0.29451 | 0.02964 | 1.83 |
| U-5 | 0.02247 | 0.03486 | 0.00409 | 0.13443 | 0.01116 | 3.85 |
| U-6 | 0.01299 | 0.01965 | 0.00125 | 0.07574 | 0.00549 | 3.85 |
| U-7 | 0.0337 | 0.041 | 0.00898 | 0.11552 | 0.01041 | 2.82 |
| U-8 | 0.02417 | 0.18994 | 0.03039 | 0.43598 | 0.01512 | 2.29 |
| U-9 | 0.02179 | 0.20601 | 0.0283 | 0.39668 | 0.03593 | 1.92 |
| U-10 | 0.04744 | 0.29338 | 0.03867 | 0.6637 | 0.09679 | 2.26 |
| U-11 | 0.03983 | 0.02581 | 0.0015 | 0.07147 | 0.00915 | 2.77 |
| U-12 | 0.04924 | 0.38879 | 0.0283 | 0.51021 | 0.05627 | 1.31 |
| U-13 | 0.00739 | 0.01735 | 0.0027 | 0.49223 | 0.04035 | 28.38 |
| U-14 | 0.00775 | 0.00905 | 0.00104 | 0.71221 | 0.06329 | 78.73 |

**Table S1.1.** Details of all statistically significant (*p* < 0.05) down-regulated (A) and up-regulated (B) proteins spots in *falciparum* malaria (compared to healthy controls) visualized in classical 2DE

FM: *Falciparum* malaria; HC: Healthy control

**Table S1.2.** Details of all statistically significant (*p* < 0.05) down-regulated (A) and up-regulated (B) proteins spots in *falciparum* malaria (compared to healthy controls) visualized in 2D-DIGE

| 1. **Down-regulated spots** | | | | | |
| --- | --- | --- | --- | --- | --- |
| **Sl. No.** | **Protein ID** | **Master No.** | **Appearance** | ***p* value**  **(t-test)** | **Av. Ratio** |
| 1 | 119 | 566 | 18 (24) | 0.015 | -1.21 |
| 2 | 68 | 234 | 21 (24) | 0.045 | -1.28 |
| 3 | 69 | 608 | 21 (24) | 0.032 | -1.35 |
| 4 | 37 | 393 | 24 (24) | 0.017 | -1.36 |
| 5 | 38 | 573 | 24 (24) | 0.038 | -1.38 |
| 6 | 120 | 139 | 18 (24) | 0.0068 | -1.4 |
| 7 | 39 | 463 | 24 (24) | 0.012 | -1.41 |
| 8 | 104 | 464 | 18 (24) | 0.0042 | -1.52 |
| 9 | 105 | 186 | 18 (24) | 0.041 | -1.53 |
| 10 | 40 | 816 | 24 (24) | 0.019 | -1.65 |
| 11 | 106 | 696 | 18 (24) | 0.014 | -1.65 |
| 12 | 70 | 524 | 21 (24) | 0.032 | -1.68 |
| 13 | 107 | 501 | 18 (24) | 0.0065 | -1.69 |
| 14 | 41 | 417 | 24 (24) | 0.014 | -1.73 |
| 15^($)^ | 42 | 485 | 24 (24) | 0.0011 | -1.79 |
| 16 | 43 | 531 | 24 (24) | 0.0026 | -1.85 |
| 17 | 71 | 447 | 21 (24) | 0.016 | -1.87 |
| 18^($)^ | 44 | 452 | 24 (24) | 0.0021 | -1.88 |
| 19 | 108 | 680 | 18 (24) | 0.0081 | -1.89 |
| 20 | 45 | 332 | 24 (24) | 0.0069 | -1.9 |
| 21 | 46 | 511 | 24 (24) | 0.015 | -1.94 |
| 22 | 72 | 1266 | 21 (24) | 0.047 | -1.97 |
| 23 | 73 | 1096 | 21 (24) | 0.02 | -2 |
| 24 | 47 | 480 | 24 (24) | 0.02 | -2.05 |
| 25 | 74 | 1172 | 21 (24) | 0.03 | -2.08 |
| 26 | 109 | 444 | 18 (24) | 0.024 | -2.08 |
| 27 | 48 | 532 | 24 (24) | 0.0032 | -2.09 |
| 28 | 121 | 981 | 18 (24) | 0.0051 | -2.1 |
| 29 | 49 | 406 | 24 (24) | 0.025 | -2.22 |
| 30 | 75 | 1177 | 21 (24) | 0.041 | -2.3 |
| 31 | 76 | 472 | 21 (24) | 0.016 | -2.35 |
| 32 | 110 | 516 | 18 (24) | 0.034 | -2.38 |
| 33 | 77 | 1323 | 21 (24) | 0.017 | -2.39 |
| 34^($)^ | 50 | 471 | 24 (24) | 0.00074 | -2.4 |
| 35 | 111 | 1174 | 18 (24) | 0.044 | -2.43 |
| 36^($)^ | 78 | 474 | 21 (24) | 0.00097 | -2.46 |
| 37^($)^ | 51 | 530 | 24 (24) | 0.0021 | -2.48 |
| 38 | 52 | 451 | 24 (24) | 0.0075 | -2.49 |
| 39 | 53 | 390 | 24 (24) | 0.033 | -2.58 |
| 40^($)^ | 54 | 520 | 24 (24) | 0.0013 | -2.59 |
| 41 | 55 | 440 | 24 (24) | 0.0066 | -2.84 |
| 42 | 56 | 421 | 24 (24) | 0.0063 | -2.95 |
| 43^($,†)^ | 57 | 466 | 24 (24) | 0.000067 | -3.05 |
| 44 | 79 | 512 | 21 (24) | 0.048 | -3.25 |
| 45 | 112 | 1330 | 18 (24) | 0.011 | -3.25 |
| 46 | 80 | 1125 | 21 (24) | 0.034 | -3.34 |
| 47 | 113 | 873 | 18 (24) | 0.011 | -4.42 |
| 48 | 58 | 1201 | 24 (24) | 0.035 | -7.38 |
| 49 | 114 | 1170 | 18 (24) | 0.0033 | -7.78 |
| 50 | 81 | 1169 | 21 (24) | 0.014 | -10.16 |
| 51 | 115 | 1375 | 18 (24) | 0.018 | -24.06 |

| 1. **Up-regulated spots** | | | | | |
| --- | --- | --- | --- | --- | --- |
| **Sl. No.** | **Protein ID** | **Master No.** | **Appearance** | ***p* value**  **(t-test)** | **Av. Ratio** |
| 52 | 118 | 217 | 18 (24) | 0.0069 | 1.02 |
| 53 | 36 | 322 | 24 (24) | 0.026 | 1.45 |
| 54 | 67 | 298 | 21 (24) | 0.049 | 1.45 |
| 55 | 103 | 633 | 18 (24) | 0.019 | 1.46 |
| 56 | 117 | 571 | 18 (24) | 0.042 | 1.46 |
| 57 | 102 | 303 | 18 (24) | 0.019 | 1.48 |
| 58 | 35 | 279 | 24 (24) | 0.038 | 1.49 |
| 59 | 99 | 318 | 18 (24) | 0.0049 | 1.5 |
| 60 | 100 | 313 | 18 (24) | 0.041 | 1.5 |
| 61 | 101 | 316 | 18 (24) | 0.045 | 1.5 |
| 62 | 33 | 287 | 24 (24) | 0.015 | 1.52 |
| 63 | 34 | 721 | 24 (24) | 0.027 | 1.52 |
| 64 | 32 | 292 | 24 (24) | 0.0095 | 1.53 |
| 65 | 31 | 265 | 24 (24) | 0.0029 | 1.55 |
| 66 | 30 | 223 | 24 (24) | 0.028 | 1.56 |
| 67 | 29 | 283 | 24 (24) | 0.0037 | 1.57 |
| 68 | 97 | 327 | 18 (24) | 0.0084 | 1.58 |
| 69 | 98 | 320 | 18 (24) | 0.011 | 1.58 |
| 70 | 116 | 320 | 18 (24) | 0.011 | 1.58 |
| 71 | 28 | 310 | 24 (24) | 0.0045 | 1.6 |
| 72^($,†)^ | 27 | 689 | 24 (24) | 0.0001 | 1.62 |
| 73 | 96 | 659 | 18 (24) | 0.022 | 1.63 |
| 74 | 66 | 293 | 21 (24) | 0.043 | 1.67 |
| 75 | 94 | 773 | 18 (24) | 0.0072 | 1.71 |
| 76 | 95 | 724 | 18 (24) | 0.013 | 1.71 |
| 77 | 65 | 788 | 21 (24) | 0.049 | 1.72 |
| 78 | 93 | 671 | 18 (24) | 0.02 | 1.74 |
| 79^($)^ | 26 | 731 | 24 (24) | 0.00032 | 1.76 |
| 80 | 25 | 321 | 24 (24) | 0.011 | 1.82 |
| 81 | 92 | 723 | 18 (24) | 0.0089 | 1.86 |
| 82^($,†)^ | 24 | 677 | 24 (24) | 5.40E-05 | 1.88 |
| 83 | 23 | 714 | 24 (24) | 0.0038 | 1.89 |
| 84 | 64 | 698 | 21 (24) | 0.007 | 1.89 |
| 85^($)^ | 22 | 759 | 24 (24) | 0.00025 | 1.9 |
| 86 | 63 | 341 | 21 (24) | 0.011 | 1.9 |
| 87 | 91 | 771 | 18 (24) | 0.0062 | 1.9 |
| 88 | 90 | 736 | 18 (24) | 0.0037 | 1.91 |
| 89 | 89 | 722 | 18 (24) | 0.0029 | 1.95 |
| 90^($)^ | 20 | 757 | 24 (24) | 0.0014 | 1.99 |
| 91 | 21 | 725 | 24 (24) | 0.01 | 1.99 |
| 92^($)^ | 19 | 730 | 24 (24) | 0.00035 | 2.02 |
| 93^($)^ | 62 | 684 | 21 (24) | 0.00084 | 2.04 |
| 94^($)^ | 88 | 772 | 18 (24) | 0.00065 | 2.04 |
| 95^($)^ | 18 | 747 | 24 (24) | 0.0008 | 2.06 |
| 96^($,†)^ | 17 | 760 | 24 (24) | 2.80E-05 | 2.07 |
| 97^($)^ | 16 | 720 | 24 (24) | 0.0004 | 2.11 |
| 98^($)^ | 87 | 726 | 18 (24) | 0.0011 | 2.13 |
| 99^($)^ | 15 | 763 | 24 (24) | 0.00021 | 2.16 |
| 100^($,†)^ | 14 | 753 | 24 (24) | 9.00E-05 | 2.17 |
| 101^($)^ | 13 | 718 | 24 (24) | 0.00035 | 2.19 |
| 102^($,†)^ | 12 | 717 | 24 (24) | 8.50E-05 | 2.2 |
| 103 | 61 | 299 | 21 (24) | 0.0033 | 2.27 |
| 104^($)^ | 60 | 699 | 21 (24) | 0.0015 | 2.28 |
| 105^($)^ | 11 | 371 | 24 (24) | 0.00025 | 2.3 |
| 106^($)^ | 10 | 306 | 24 (24) | 0.00065 | 2.32 |
| 107^($)^ | 9 | 764 | 24 (24) | 0.00058 | 2.38 |
| 108^($)^ | 8 | 294 | 24 (24) | 0.0017 | 2.4 |
| 109^($)^ | 86 | 746 | 18 (24) | 0.00023 | 2.4 |
| 110 | 59 | 783 | 21 (24) | 0.044 | 2.44 |
| 111^($,†)^ | 7 | 710 | 24 (24) | 2.60E-05 | 2.47 |
| 112 | 6 | 388 | 24 (24) | 0.0052 | 2.51 |
| 113^($)^ | 5 | 376 | 24 (24) | 0.0016 | 2.52 |
| 114^($)^ | 4 | 297 | 24 (24) | 0.00022 | 2.67 |
| 115 | 85 | 701 | 18 (24) | 0.0046 | 2.82 |
| 116^($)^ | 84 | 339 | 18 (24) | 0.0015 | 3 |
| 117 | 83 | 1233 | 18 (24) | 0.045 | 3.55 |
| 118^($)^ | 3 | 702 | 24 (24) | 0.00018 | 3.65 |
| 119 | 82 | 1229 | 18 (24) | 0.044 | 5.05 |
| 120^($,†)^ | 2 | 1248 | 24 (24) | 2.80E-05 | 28.64 |
| 121^($,†)^ | 1 | 1231 | 24 (24) | 2.00E-06 | 50.9 |

^$^ Protein spots significant after false discovery rate (FDR) correction (Benjamini-Hochberg)

† Protein spots significant after Bonferroni correction

**Table S1.3.** Details of all statistically significant (*p* < 0.05) down-regulated (A) and up-regulated (B) proteins spots in *falciparum* malaria (compared to *vivax* malaria) visualized in 2D-DIGE

| 1. **Down-regulated spots** | | | | | |
| --- | --- | --- | --- | --- | --- |
| **Sl. No.** | **Protein ID** | **Master No.** | **Appearance** | ***p* value**  **(t-test)** | **Av. Ratio** |
| 1 | 318 | 154 | 39 (48) | 0.036 | -1.28 |
| 2 | 112 | 213 | 45 (48) | 0.033 | -1.32 |
| 3 | 17 | 312 | 48 (48) | 0.037 | -1.39 |
| 4 | 190 | 683 | 45 (48) | 0.024 | -1.41 |
| 5 | 293 | 998 | 42 (48) | 0.01 | -1.44 |
| 6 | 360 | 624 | 39 (48) | 0.024 | -1.44 |
| 7 | 427 | 121 | 36 (48) | 0.044 | -1.44 |
| 8 | 152 | 428 | 45 (48) | 0.019 | -1.45 |
| 9 | 70 | 615 | 48 (48) | 0.035 | -1.45 |
| 10 | 323 | 173 | 39 (48) | 0.0054 | -1.48 |
| 11 | 26 | 335 | 48 (48) | 0.035 | -1.48 |
| 12 | 470 | 590 | 36 (48) | 0.03 | -1.49 |
| 13 | 357 | 596 | 39 (48) | 0.032 | -1.49 |
| 14 | 128 | 310 | 45 (48) | 0.035 | -1.49 |
| 15 | 187 | 668 | 45 (48) | 0.0091 | -1.5 |
| 16 | 310 | 99 | 39 (48) | 0.034 | -1.5 |
| 17 | 21 | 320 | 48 (48) | 0.0022 | -1.52 |
| 18 | 319 | 155 | 39 (48) | 0.0073 | -1.52 |
| 19 | 27 | 336 | 48 (48) | 0.022 | -1.52 |
| 20 | 453 | 470 | 36 (48) | 0.0088 | -1.56 |
| 21 | 212 | 111 | 42 (48) | 0.0054 | -1.57 |
| 22 | 30 | 343 | 48 (48) | 0.024 | -1.58 |
| 23 | 151 | 425 | 45 (48) | 0.022 | -1.59 |
| 24 | 76 | 679 | 48 (48) | 0.0022 | -1.6 |
| 25 | 173 | 546 | 45 (48) | 0.0053 | -1.6 |
| 26 | 460 | 510 | 36 (48) | 0.042 | -1.6 |
| 27^($)^ | 107 | 164 | 45 (48) | 0.00029 | -1.64 |
| 28 | 65 | 591 | 48 (48) | 0.0012 | -1.64 |
| 29 | 51 | 498 | 48 (48) | 0.003 | -1.65 |
| 30 | 40 | 381 | 48 (48) | 0.0052 | -1.66 |
| 31^($,†)^ | 358 | 600 | 39 (48) | 6.50E-05 | -1.68 |
| 32^($)^ | 64 | 582 | 48 (48) | 0.00023 | -1.7 |
| 33^($)^ | 3 | 186 | 48 (48) | 0.00062 | -1.7 |
| 34 | 4 | 202 | 48 (48) | 0.017 | -1.7 |
| 35 | 243 | 374 | 42 (48) | 0.013 | -1.71 |
| 36 | 266 | 585 | 42 (48) | 0.011 | -1.72 |
| 37 | 116 | 241 | 45 (48) | 0.05 | -1.75 |
| 38^($)^ | 22 | 326 | 48 (48) | 0.00039 | -1.76 |
| 39^($)^ | 61 | 566 | 48 (48) | 0.00039 | -1.77 |
| 40 | 126 | 291 | 45 (48) | 0.0015 | -1.77 |
| 41 | 68 | 605 | 48 (48) | 0.0062 | -1.78 |
| 42^($)^ | 24 | 330 | 48 (48) | 0.00039 | -1.79 |
| 43 | 115 | 235 | 45 (48) | 0.018 | -1.81 |
| 44 | 271 | 616 | 42 (48) | 0.0013 | -1.83 |
| 45 | 25 | 331 | 48 (48) | 0.0021 | -1.83 |
| 46 | 362 | 637 | 39 (48) | 0.025 | -1.84 |
| 47 | 305 | 51 | 39 (48) | 0.0096 | -1.85 |
| 48^($)^ | 246 | 391 | 42 (48) | 0.00059 | -1.87 |
| 49 | 253 | 433 | 42 (48) | 0.0076 | -1.88 |
| 50^($,†)^ | 181 | 601 | 45 (48) | 0.00012 | -1.94 |
| 51 | 449 | 431 | 36 (48) | 0.045 | -1.95 |
| 52 | 341 | 395 | 39 (48) | 0.0062 | -2 |
| 53 | 469 | 586 | 36 (48) | 0.033 | -2.15 |
| 54 | 354 | 565 | 39 (48) | 0.0011 | -2.29 |
| 55 | 374 | 736 | 39 (48) | 0.0011 | -2.39 |
| 56 | 256 | 461 | 42 (48) | 0.00084 | -2.52 |
| 57 | 403 | 1122 | 39 (48) | 0.033 | -2.66 |
| 58 | 359 | 602 | 39 (48) | 0.048 | -2.77 |
| 59 | 505 | 1098 | 36 (48) | 0.013 | -3.67 |
| 60 | 96 | 1016 | 48 (48) | 0.013 | -5.69 |
| 61 | 163 | 511 | 45 (48) | 0.0046 | -7.06 |

| 1. **Up-regulated spots** | | | | | |
| --- | --- | --- | --- | --- | --- |
| **Sl. No.** | **Protein ID** | **Master No.** | **Appearance** | ***p* value**  **(t-test)** | **Av. Ratio** |
| 62 | 84 | 772 | 48 (48) | 0.029 | 1.22 |
| 63 | 250 | 414 | 42 (48) | 0.021 | 1.45 |
| 64 | 245 | 386 | 42 (48) | 0.024 | 1.46 |
| 65 | 251 | 422 | 42 (48) | 0.026 | 1.5 |
| 66 | 39 | 380 | 48 (48) | 0.032 | 1.54 |
| 67 | 476 | 705 | 36 (48) | 0.014 | 1.55 |
| 68 | 18 | 313 | 48 (48) | 0.022 | 1.57 |
| 69 | 431 | 197 | 36 (48) | 0.037 | 1.68 |
| 70^($,†)^ | 343 | 420 | 39 (48) | 9.40E-06 | 1.69 |
| 71 | 332 | 281 | 39 (48) | 0.0061 | 1.78 |
| 72 | 124 | 288 | 45 (48) | 0.0034 | 1.79 |
| 73 | 38 | 379 | 48 (48) | 0.047 | 1.86 |
| 74 | 196 | 817 | 45 (48) | 0.017 | 1.9 |
| 75 | 475 | 700 | 36 (48) | 0.015 | 1.94 |
| 76 | 41 | 388 | 48 (48) | 0.0021 | 1.96 |
| 77 | 143 | 389 | 45 (48) | 0.0088 | 1.98 |
| 78 | 291 | 878 | 42 (48) | 0.0024 | 2.29 |
| 89 | 147 | 402 | 45 (48) | 0.017 | 2.36 |
| 80 | 516 | 1319 | 36 (48) | 0.037 | 2.62 |
| 81 | 302 | 1420 | 42 (48) | 0.045 | 6.13 |
| 82 | 412 | 1414 | 39 (48) | 0.0053 | 7.09 |
| 83 | 496 | 930 | 36 (48) | 0.0075 | 12.08 |
| 84 | 408 | 1308 | 39 (48) | 0.0098 | 12.63 |

^$^ Protein spots significant after false discovery rate (FDR) correction (Benjamini-Hochberg)

† Protein spots significant after Bonferroni correction
