# Supplementary material for: Proteomic Investigation of Falciparum and Vivax Malaria for Identification of Surrogate Protein Markers
Source: PLoS One. 2012 Aug 9;7(8):e41751. doi: 10.1371/journal.pone.0041751 (PMC3415403; doi:10.1371/journal.pone.0041751)
Supplement: Table S2 — Master tables for all MALDI-TOF/TOF identified proteins in falciparum malaria. (DOC) [file pone.0041751.s011.doc]

**Table S2.** Master tables for all MALDI-TOF/TOF identified proteins in *falciparum* malaria

**Table S2.1.** Master table for all MALDI-TOF/TOF identified proteins in *falciparum* malaria from classical 2DE experiment ^$^

| **Sl No** | **Spot ID** | **Fold**  **Change#** | Name of protein with UniProt **accession number** | **MW**  **(kDa)** | **Prote-in score** | **Total ion score** | **No. of matched**  **peptides** | **Peptide sequence** |
| --- | --- | --- | --- | --- | --- | --- | --- | --- |
| 1 | D-1 | - 4.28 | (P00738) Haptoglobin precursor | 45.17 | 217 | 150 | 13 | DYAEVGR  QLVEIEK  GSFPWQAK  ILGGHLDAK  VGYVSGWGR  VMPICLPSK  VTSIQDWVQK  DIAPTLTLYVGK  SCAVAEYGVYVK  YVMLPVADQDQCIR  VSVNERVMPICLPSK  VMPICLPSKDYAEVGR  SPVGVQPILNEHTFCAGMSK |
| 2 | D-2 | -1.58 | (O75636) Ficolin 3 precursor (Collagen/fibrinogen domain-containing protein 3) | 32.9 | 515 | 463 | 10 | VRMMLR  YGIDWASGR  QDGSVDFFR  TFAHYATFR  GEPGDPVNLLR  RQDGSVDFFR  LLGEVDHYQLALGK  ALPVFCDMDTEGGGWLVFQR  ELLSQGATLSGWYHLCLPEGR  *AGFGNQESEFWLGNENLHQLTLQGNWELR* |
| 3 | D-3 | -1.39 | (P02768) Serum albumin precursor | 69.32 | 456 | 386 | 30 | CCKADDK  YLYEIAR  LCTVATLR  DDNPNLPR  LKCASLQK  FQNALLVR  LVNEVTEFAK  FKDLGEENFK  HPDYSVVLLLR  AVMDDFAAFVEK  RHPDYSVVLLLR  CCAAADPHECYAK  DVFLGMFLYEYAR  KVPQVSTPTLVEVSR  QEPERNECFLQHK  HPYFYAPELLFFAK  RHPYFYAPELLFFAK  HPYFYAPELLFFAKR  RPCFSALEVDETYVPK  VFDEFKPLVEEPQNLIK  EFNAETFTFHADICTLSEK  ALVLIAFAQYLQQCPFEDHVK  TCVADESAENCDKSLHTLFGDK  MPCAEDYLSVVLNQLCVLHEK  EFNAETFTFHADICTLSEKER  QNCELFEQLGEYKFQNALLVR  RMPCAEDYLSVVLNQLCVLHEK  LVRPEVDVMCTAFHDNEETFLK  RMPCAEDYLSVVLNQLCVLHEK  LVRPEVDVMCTAFHDNEETFLKK |
| 4 | D-4 | -1.91 | (P10909) Clusterin precursor (Complement-associated protein SP-40) | 52.46 | 151 | 135 | 8 | VCRSGSGLVGR  EGDDDRTVCR  ELDESLQVAER  NPKFMETVAEK  ASSIIDELFQDR  EILSVDCSTNNPSQAK  LFDSDPITVTVPVEVSR  VTTVASHTSDSDVPSGVTEVVVK |
| 5 | D-5 | -2.12 | (P00738) Haptoglobin precursor | 45.17 | 248 | 198 | 12 | NYYKLR  NPANPVQR  VMPICLPSK  YQCKNYYK  LPECEAVCGKPK  TEGDGVYTLNDKK  LRTEGDGVYTLNDK  YVMLPVADQDQCIR  LRTEGDGVYTLNNEK  AVGDKLPECEAVCGKPK  LPECEADDGCPKPPEIAHGYVEHSVR  *AVGDKLPECEADDGCPKPPEIAHGYVEHSVR* |
| 6 | D-6 | -1.58 | (P02647) Apolipoprotein A-I precursor (Apo-AI) | 30.75 | 486 | 351 | 20 | QKVEPLR  AELQEGAR  LHELQEK  AKPALEDLR  LSPLGEEMR  QKLHELQEK  QGLLPVLESFK  DLATVYVDVLK  VQPYLDDFQK  WQEEMELYR  THLAPYSDELR  LSPLGEEMRDR  VQPYLDDFQKK  VSFLSALEEYTK  DYVSQFEGSALGK  KWQEEMELYR  VEPLRAELQEGAR  LLDNWDSVTSTFSK  DSGRDYVSQFEGSALGK  LREQLGPVTQEFWDNLEK |
| 7 | D-7 | -1.80 | (P02768) Serum albumin precursor | 69.32 | 663 | 568 | 26 | CCKADDK  YLYEIAR  DDNPNLPR  FQNALLVR  CASLQKFGER  HPDYSVVLLLR  AVMDDFAAFVEK  RHPDYSVVLLLR  CCAAADPHECYAK  DVFLGMFLYEYAR  KVPQVSTPTLVEVSR  QNCELFEQLGEYK  QEPERNECFLQHK  HPYFYAPELLFFAK  RPCFSALEVDETYVPK  NECFLQHKDDNPNLPR  VFDEFKPLVEEPQNLIK  VHTECCHGDLLECADDR  EFNAETFTFHADICTLSEK  MPCAEDYLSVVLNQLCVLHEK  EFNAETFTFHADICTLSEKER  VHTECCHGDLLECADDRADLAK  LVRPEVDVMCTAFHDNEETFLK  RMPCAEDYLSVVLNQLCVLHEK  LVRPEVDVMCTAFHDNEETFLKK  CCTESLVNRRPCFSALEVDETYVPK |
| 8 | D-8 | -2.02 | (P02647) Apolipoprotein A-I precursor (Apo-AI) | 30.75 | 509 | 268 | 25 | LAEYHAK  QKVEPLR  AELQEGAR  LHELQEK  AKPALEDLR  LSPLGEEMR  QKLHELQEK  QGLLPVLESFK  DLATVYVDVLK  VQPYLDDFQK  WQEEMELYR  THLAPYSDELR  ETEGLRQEMSK  LSPLGEEMRDR  VQPYLDDFQKK  VSFLSALEEYTK  DYVSQFEGSALGK  KWQEEMELYR  VKDLATVYVDVLK  VEPLRAELQEGAR  LLDNWDSVTSTFSK  DSGRDYVSQFEGSALGK  EQLGPVTQEFWDNLEK  HFWQQDEPPQSPWDR  LREQLGPVTQEFWDNLEK |
| 9 | U-1 | 2.00 | (P02790) Hemopexin precursor (Beta-1B-glycoprotein) | 51.64 | 239 | 171 | 14 | LHIMAGR  LWWLDLK  RLWWLDLK  DYFMPCPGR  VWVYPPEKK  FDPVRGEVPPR  YYCFQGNQFLR  GECQAEGVLFFQGDR  ALPQPQNVTSLLGCTH  LYLVQGTQVYVFLTK  SGAQATWTELPWPHEK  LLQDEFPGIPSPLDAAVECHR  EVGTPHGIILDSVDAAFICPGSSR  *SLGPNSCSANGPGLYLIHGPNLYCYSDVEK* |
| 10 | U-2 | 3.28 | (P01009) Alpha-1-antitrypsin precursor (Alpha-1 protease inhibitor) | 46.70 | 243 | 84 | 24 | SASLHLPK  RSASLHLPK  SVLGQLGITK  LSSWVLLMK  FLENEDRR  LSSWVLLMK  LSITGTYDLK  KLSSWVLLMK  LGMFNIQHCK  GKWERPFEVK  RLGMFNIQHCK  LGMFNIQHCKK  RLGMFNIQHCK  QINDYVEKGTQGK  ITPNLAEFAFSLYR  VFSNGADLSGVTEEAPLK  FNKPFVFLMIEQNTK  ELDRDTVFALVNYIFFK  LSITGTYDLKSVLGQLGITK  VFSNGADLSGVTEEAPLKLSK  KLYHSEAFTVNFGDTEEAK  LYHSEAFTVNFGDTEEAKK  TLNQPDSQLQLTTGNGLFLSEGLK  *TDTSHHDQDHPTFNKITPNLAEFAFSLYR* |
| 11 | U-3 | 1.78 | (P01009) Alpha-1-antitrypsin precursor (Alpha-1 protease inhibitor) | 46.70 | 151 | 66 | 16 | FLEDVKK  AVLTIDEK  FLENEDR  QINDYVEK  RSASLHLPK  SVLGQLGITK  FLENEDRR  WERPFEVK  LSITGTYDLK  GKWERPFEVK  DTVFALVNYIFFK  TDTSHHDQDHPTFNK  LQHLENELTHDIITK  DTEEEDFHVDQVTTVK  ELDRDTVFALVNYIFFK  LYHSEAFTVNFGDTEEAKK |
| 12 | U-4 | 1.83 | (P02649) Apolipoprotein E precursor (Apo-E) | 36.13 | 1100 | 955 | 21 | DADDLQK  AQAWGER  EQVAEVR  FWDYLR  WELALGR  ELQAAQAR  QWAGLVEK  LAVYQAGAR  LGPLVEQGR  LQAEAFQAR  LEEQAQQIR  LGADMEDVCGR  QQTEWQSGQR  AKLEEQAQQIR  AATVGSLAGQPLQER  SWFEPLVEDMQR  VEQAVETEPEPELR  GEVQAMLGQSTEELR  SELEEQLTPVAEETR  AYKSELEEQLTPVAEETR  WVQTLSEQVQEELLSSQVTQELR |
| 13 | U-5 | 3.85 | (P02750) Leucine-rich alpha-2-glycoprotein precursor (LRG) | 38.15 | 637 | 592 | 10 | GPLQLER  DCQVFR  VAAGAFQGLR  ALGHLDLSGNR  DLLLPQPDLR  ENQLEVLEVSWLHGLK  TLDLGENQLETLPPDLLR  NALTGLPPGLFQASATLDTLVLK  DGFDISGNPWICDQNLSDLYR  LQELHLSSNGLESLSPEFLRPVPQLR |
| 14 | U-6 | 3.85 | (P02750) Leucine-rich alpha-2-glycoprotein precursor (LRG) | 38.15 | 675 | 621 | 11 | GPLQLER  DCQVFR  VAAGAFQGLR  ALGHLDLSGNR  DLLLPQPDLR  LARVAAGAFQGLR  ENQLEVLEVSWLHGLK  TLDLGENQLETLPPDLLR  NALTGLPPGLFQASATLDTLVLK  DGFDISGNPWICDQNLSDLYR  LQELHLSSNGLESLSPEFLRPVPQLR |
| 15 | U-7 | 2.82 | (P02790) Hemopexin precursor (Beta-1B-glycoprotein) | 51.64 | 239 | 171 | 14 | LHIMAGR  LWWLDLK  RLWWLDLK  DYFMPCPGR  VWVYPPEKK  FDPVRGEVPPR  YYCFQGNQFLR  GECQAEGVLFFQGDR  ALPQPQNVTSLLGCTH  LYLVQGTQVYVFLTK  SGAQATWTELPWPHEK  LLQDEFPGIPSPLDAAVECHR  EVGTPHGIILDSVDAAFICPGSSR  *SLGPNSCSANGPGLYLIHGPNLYCYSDVEK* |
| 16 | U-8 | 2.29 | (P01011) Alpha-1-antichymotrypsin precursor (ACT) | 47.62 | 184 | 75 | 15 | ADLSGITGAR  EQLSLLDR  EIGELYLPK  WRDSLEFR  ITLLSALVETR  MEEVEAMLLPETLK  EQLSLLDRFTEDAK  WEMPFDPQDTHQSR  MEEVEAMLLPETLKR  LYGSEAFATDFQDSAAAK  AVLDVFEEGTEASAATAVK  AKWEMPFDPQDTHQSR  LYGSEAFATDFQDSAAAKK  RLYGSEAFATDFQDSAAAK  GTHVDLGLASANVDFAFSLYK |
| 17 | U-9 | 1.92 | (P01011) Alpha-1-antichymotrypsin precursor (ACT) | 47.62 | 258 | 182 | 15 | ADLSGITGAR  EQLSLLDR  EIGELYLPK  WRDSLEFR  ITLLSALVETR  WEMPFDPQDTHQSR  MEEVEAMLLPETLKR  LYGSEAFATDFQDSAAAK  AVLDVFEEGTEASAATAVK  AKWEMPFDPQDTHQSR  RLYGSEAFATDFQDSAAAK  DLDSQTMMVLVNYIFFK  GTHVDLGLASANVDFAFSLYK  DYNLNDILLQLGIEEAFTSK  FNRPFLMIIVPTDTQNIFFMSK |
| 18 | U-10 | 2.26 | (P01011) Alpha-1-antichymotrypsin precursor (ACT) | 47.62 | 284 | 130 | 20 | ADLSGITGAR  EQLSLLDR  KLINDYVK  EIGELYLPK  NLAVSQVVHK  WRDSLEFR  ITLLSALVETR  EQLSLLDRFTEDAK  WEMPFDPQDTHQSR  MEEVEAMLLPETLKR  LYGSEAFATDFQDSAAAK  AVLDVFEEGTEASAATAVK  AKWEMPFDPQDTHQSR  LYGSEAFATDFQDSAAAKK  RLYGSEAFATDFQDSAAAK  DLDSQTMMVLVNYIFFK  GTHVDLGLASANVDFAFSLYK  WVMVPMMSLHHLTIPYFR  YNLNDILLQLGIEEAFTSK  FNRPFLMIIVPTDTQNIFFMSK |
| 19 | U-11 | 2.77 | (P02649) Apolipoprotein E precursor | 36.13 | 1100 | 955 | 21 | DADDLQK  AQAWGER  EQVAEVR  FWDYLR  WELALGR  ELQAAQAR  QWAGLVEK  LAVYQAGAR  LGPLVEQGR  LQAEAFQAR  LEEQAQQIR  LGADMEDVCGR  QQTEWQSGQR  AKLEEQAQQIR  AATVGSLAGQPLQER  SWFEPLVEDMQR  VEQAVETEPEPELR  GEVQAMLGQSTEELR  SELEEQLTPVAEETR  AYKSELEEQLTPVAEETR  WVQTLSEQVQEELLSSQVTQELR |
| 20 | U-12 | 1.31 | (P04217) Alpha-1B-glycoprotein precursor (Alpha-1-BN glycoprotein) | 54.3 | 900 | 821 | 16 | GVTFLLR  LLELTGPK  CLAPLEGAR  ATWSGAVLAGR  LETPDFQLFK  SGLSTGWTQLSK  HQFLLTGDTQGR  CEGPIPDVTFELLR  NGVAQEPVHLDSPAIK  LELHVDGPPPRPQLR  VTLTCVAPLSGVDFQLR  IFFHLNAVALGDGGHYTCR  SLPAPWLSMAPVSWITPGLK  TPGAAANLELIFVGPQHAGNYR  SWVPHTFESELSDPVELLVAES  *LHDNQNGWSGDSAPVELILSDETLPAPEFSPEPESGR* |
| 21 | U-13 | 28.38 | (P02735) Serum amyloid A (SAA) | 13.52 | 212 | 164 | 7 | GPGGVWAAEAISDAR  SFFSFLGEAFDGAR  RGPGGVWAAEAISDAR  DPNHFRPAGLPEKY  EANYIGSDKYFHAR  MKLLTGLVFCSLVLGVSSR  FFGHGAEDSLADQAANEWGR |
| 22 | U-14 | 78.73 | (P02735) Serum amyloid A (SAA) | 13.52 | 215 | 176 | 6 | GPGGVWAAEAISDAR  SFFSFLGEAFDGAR  RGPGGVWAAEAISDAR  DPNHFRPAGLPEKY  EANYIGSDKYFHAR  FFGHGAEDSLADQAANEWGR |

^$^ [Analysis Type: Combined (MS+MS/MS); Database: SwissProt; Taxonomy: All entries]

# Alterations in protein expression levels in *falciparum* malaria (FM) were measured using healthy subjects as controls

* indicates the continuation of peptide sequence in the next line

**Table S2.2. Master table for all MALDI-TOF/TOF identified proteins in *falciparum* malaria from 2D-DIGE experiment^$^**

| **Sl No** | **Spot ID** | **Fold**  **Change#** | **Name of protein with UniProt accession number** | **MW**  **(kDa)** | **Protein score** | **Total ion score** | **No. of matched**  **peptides** | **Peptide sequence** |
| --- | --- | --- | --- | --- | --- | --- | --- | --- |
| 1 | 1 | 50.9 | (P02735) Serum amyloid A (SAA) | 13.52 | 212 | 164 | 7 | GPGGVWAAEAISDAR  SFFSFLGEAFDGAR  RGPGGVWAAEAISDAR  DPNHFRPAGLPEKY  EANYIGSDKYFHAR  MKLLTGLVFCSLVLGVSSR  FFGHGAEDSLADQAANEWGR |
| 2 | 2 | 28.64 | (P02735) Serum amyloid A (SAA) | 13.52 | 215 | 176 | 6 | GPGGVWAAEAISDAR  SFFSFLGEAFDGAR  RGPGGVWAAEAISDAR  DPNHFRPAGLPEKY  EANYIGSDKYFHAR  FFGHGAEDSLADQAANEWGR |
| 3 | 4 | 2.67 | (P01009) Alpha-1-antitrypsin precursor (Alpha-1protease inhibitor) | 46.70 | 505 | 383 | 20 | SVLGQLGITK  LSSWVLLMK  FLENEDRR  WERPFEVK  KLSSWVLLMK  LGMFNIQHCK  GKWERPFEVK  RLGMFNIQHCK  DTVFALVNYIFFK  ITPNLAEFAFSLYR  TDTSHHDQDHPTFNK  LQHLENELTHDIITK  FNKPFVFLMIEQNTK  DTEEEDFHVDQVTTVK  LYHSEAFTVNFGDTEEAK  ELDRDTVFALVNYIFFK  LYHSEAFTVNFGDTEEAKK  GTEAAGAMFLEAIPMSIPPEVK  TLNQPDSQLQLTTGNGLFLSEGLK  LQHLENELTHDIITKFLENEDR |
| 4 | 5 | 2.52 | (P02750) Leucine-rich alpha-2-glycoprotein precursor (LRG) | 38.15 | 637 | 592 | 10 | GPLQLER  DCQVFR  VAAGAFQGLR  ALGHLDLSGNR  DLLLPQPDLR  ENQLEVLEVSWLHGLK  TLDLGENQLETLPPDLLR  NALTGLPPGLFQASATLDTLVLK  DGFDISGNPWICDQNLSDLYR  LQELHLSSNGLESLSPEFLRPVPQLR |
| 5 | 6 | 2.51 | (P02750) Leucine-rich alpha-2-glycoprotein precursor (LRG) | 38.15 | 675 | 621 | 11 | GPLQLER  DCQVFR  VAAGAFQGLR  ALGHLDLSGNR  DLLLPQPDLR  LARVAAGAFQGLR  ENQLEVLEVSWLHGLK  TLDLGENQLETLPPDLLR  NALTGLPPGLFQASATLDTLVLK  DGFDISGNPWICDQNLSDLYR  LQELHLSSNGLESLSPEFLRPVPQLR |
| 6 | 8 | 2.4 | (P04004) Vitronectin precursor (Serum spreading factor) (S-protein) | 54.27 | 184 | 164 | 7 | GRNQNSR  QPQFISR  AVRPGYPK  NGSLFAFR  GQYCYELDEK  FEDGVLDPDYPR  DWHGVPGQVDAAMAGR |
| 7 | 10 | 2.32 | (P01009) Alpha-1-antitrypsin precursor (Alpha-1 protease inhibitor) | 46.70 | 151 | 66 | 16 | FLEDVKK  AVLTIDEK  FLENEDR  QINDYVEK  RSASLHLPK  SVLGQLGITK  FLENEDRR  WERPFEVK  LSITGTYDLK  GKWERPFEVK  DTVFALVNYIFFK  TDTSHHDQDHPTFNK  LQHLENELTHDIITK  DTEEEDFHVDQVTTVK  ELDRDTVFALVNYIFFK  LYHSEAFTVNFGDTEEAKK |
| 8 | 11 | 2.3 | (P02750) Leucine-rich alpha-2-glycoprotein precursor | 38.15 | 260 | 240 | 6 | GPLQLER  CAGPEAVK  VAAGAFQGLR  ALGHLDLSGNR  DLLLPQPDLR  TLDLGENQLETLPPDLLR |
| 9 | 14 | 2.17 | (P01834) Ig kappa chain C region | 11.6 | 217 | 196 | 4 | SGTASVVCLLNNFYPR  VYACEVTHQGLSSPVTK  TVAAPSVFIFPPSDEQLK  VDNALQSGNSQESVTEQDSK |
| 10 | 22 | 1.9 | (P01834) Ig kappa chain C region | 11.6 | 216 | 196 | 4 | SGTASVVCLLNNFYPR  VYACEVTHQGLSSPVTK  TVAAPSVFIFPPSDEQLK  VDNALQSGNSQESVTEQDSK |
| 11 | 25 | 1.82 | (P01009) Alpha-1-antitrypsin precursor | 46.70 | 670 | 538 | 23 | QINDYVEK  SVLGQLGITK  LSSWVLLMK  FLENEDRR  WERPFEVK  LSITGTYDLK  IVDLVKELDR  KLSSWVLLMK  LGMFNIQHCK  GKWERPFEVK  RLGMFNIQHCK  ITPNLAEFAFSLYR  TDTSHHDQDHPTFNK  LQHLENELTHDIITK  VFSNGADLSGVTEEAPLK  FNKPFVFLMIEQNTK  DTEEEDFHVDQVTTVK  LYHSEAFTVNFGDTEEAK  ELDRDTVFALVNYIFFK  LYHSEAFTVNFGDTEEAKK  GTEAAGAMFLEAIPMSIPPEVK  TLNQPDSQLQLTTGNGLFLSEGLK  WERPFEVKDTEEEDFHVDQVTTVK |
| 12 | 26 | 1.76 | (P01834) Ig kappa chain C region | 11.6 | 94 | 87 | 3 | SGTASVVCLLNNFYPR  VYACEVTHQGLSSPVTK  TVAAPSVFIFPPSDEQLK |
| 13 | 29 | 1.57 | (P01011)Alpha-1-antichymotrypsin precursor (ACT) | 47.62 | 284 | 130 | 20 | ADLSGITGAR  EQLSLLDR  KLINDYVK  EIGELYLPK  NLAVSQVVHK  WRDSLEFR  ITLLSALVETR  EQLSLLDRFTEDAK  WEMPFDPQDTHQSR  MEEVEAMLLPETLKR  LYGSEAFATDFQDSAAAK  AVLDVFEEGTEASAATAVK  AKWEMPFDPQDTHQSR  LYGSEAFATDFQDSAAAKK  RLYGSEAFATDFQDSAAAK  DLDSQTMMVLVNYIFFK  GTHVDLGLASANVDFAFSLYK  WVMVPMMSLHHLTIPYFR  YNLNDILLQLGIEEAFTSK  FNRPFLMIIVPTDTQNIFFMSK |
| 14 | 30 | 1.56 | (P01871) Ig mu chain C region | 49.52 | 522 | 484 | 15 | VSVFVPPR  DGFFGNPR  QIQVSWLR  DGFFGNPRSK  LICQATGFSPR  NVPLPVIAELPPK  YVTSAPMPEPQAPGR  VFAIPPSFASIFLTK  ESDWLSQSMFTCR  FTCTVTHTDLPSPLK  GVALHRPDVYLLPPAR  ESDWLSQSMFTCRVDHR  GLTFQQNASSMCVPDQDTAIR  YAATSQVLLPSKDVMQGTDEHVVCK  *YFAHSILTVSEEEWNTGETYTCVVAHEALPNR* |
| 15 | 31 | 1.55 | (P01009) Alpha-1-antitrypsin precursor (Alpha-1protease inhibitor) | 46.7 | 243 | 84 | 22 | SASLHLPK  RSASLHLPK  SVLGQLGITK  LSSWVLLMK  FLENEDRR  LSITGTYDLK  KLSSWVLLMK  LGMFNIQHCK  GKWERPFEVK  RLGMFNIQHCK  LGMFNIQHCKK  QINDYVEKGTQGK  ITPNLAEFAFSLYR  VFSNGADLSGVTEEAPLK  FNKPFVFLMIEQNTK  ELDRDTVFALVNYIFFK  LSITGTYDLKSVLGQLGITK  VFSNGADLSGVTEEAPLKLSK  KLYHSEAFTVNFGDTEEAK  LYHSEAFTVNFGDTEEAKK  TLNQPDSQLQLTTGNGLFLSEGLK  *TDTSHHDQDHPTFNKITPNLAEFAFSLYR* |
| 16 | 32 | 1.53 | (P01011) Alpha-1-antichymotrypsin precursor (ACT) | 47.62 | 184 | 75 | 15 | ADLSGITGAR  EQLSLLDR  EIGELYLPK  WRDSLEFR  ITLLSALVETR  MEEVEAMLLPETLK  EQLSLLDRFTEDAK  WEMPFDPQDTHQSR  MEEVEAMLLPETLKR  LYGSEAFATDFQDSAAAK  AVLDVFEEGTEASAATAVK  AKWEMPFDPQDTHQSR  LYGSEAFATDFQDSAAAKK  RLYGSEAFATDFQDSAAAK  GTHVDLGLASANVDFAFSLYK |
| 17 | 33 | 1.52 | (P01011) Alpha-1-antichymotrypsin precursor (ACT) | 47.62 | 258 | 182 | 15 | ADLSGITGAR  EQLSLLDR  EIGELYLPK  WRDSLEFR  ITLLSALVETR  WEMPFDPQDTHQSR  MEEVEAMLLPETLKR  LYGSEAFATDFQDSAAAK  AVLDVFEEGTEASAATAVK  AKWEMPFDPQDTHQSR  RLYGSEAFATDFQDSAAAK  DLDSQTMMVLVNYIFFK  GTHVDLGLASANVDFAFSLYK  DYNLNDILLQLGIEEAFTSK  FNRPFLMIIVPTDTQNIFFMSK |
| 18 | 35 | 1.49 | (P01011) Alpha-1-antichymotrypsin precursor (ACT) | 47.62 | 189 | 84 | 18 | LINDYVK  FTEDAKR  ADLSGITGAR  EQLSLLDR  KLINDYVK  EIGELYLPK  NLAVSQVVHK  WRDSLEFR  ITLLSALVETR  MEEVEAMLLPETLK  WEMPFDPQDTHQSR  LYGSEAFATDFQDSAAAK  AVLDVFEEGTEASAATAVK  AKWEMPFDPQDTHQSR  DLDSQTMMVLVNYIFFK  GTHVDLGLASANVDFAFSLYK  DYNLNDILLQLGIEEAFTSK  FNRPFLMIIVPTDTQNIFFMSK |
| 19 | 36 | 1.45 | (P01009) Alpha-1-antitrypsin precursor (Alpha-1 protease inhibitor) | 46.70 | 152 | 84 | 14 | AVLTIDEK  FLENEDR  QINDYVEK  RSASLHLPK  SVLGQLGITK  LSSWVLLMK  FLENEDRR  WERPFEVK  GKWERPFEVK  DTVFALVNYIFFK  LQHLENELTHDIITK  DTEEEDFHVDQVTTVK  ELDRDTVFALVNYIFFK  LYHSEAFTVNFGDTEEAKK |
| 20 | 37 | -1.36 | (P49802) Regulator of G-protein signaling 7 | 75.79 | 28 | - | 12 | IPNSQRK  LCKSGDCR  CQGSRLDDQR  HSMENMELMK  HLAIAQELNDR  HLEISREVGDK  DQNRDTDFGLK  MREDHSFHVR  MEASCLELALEGER  VLGNFDEAIVCCQR  DFLQNNALLEFKNSGK  TSSVPVVSPNTDEFLDLLASSQSRR |
| 21 | 39 | -1.41 | (P02787) Serotransferrin precursor (Transferrin) | 76.99 | 1160 | 906 | 42 | NPDPWAK  SCHTAVGR  APNHAVVTR  ASYLDCIR  YLGEEYVK  KASYLDCIR  WCALSHHER  DSGFQMNQLR  SASDLTWDNLK  HSTIFENLANK  EFQLFSSPHGK  EGYYGYTGAFR  WCAVSEHEATK  KDSGFQMNQLR  DYELLCLDGTR  KSASDLTWDNLK  SVIPSDGPSVACVK  MYLGYEYVTAIR  SKEFQLFSSPHGK  LKCDEWSVNSVGK  CSTSSLLEACTFR  DQYELLCLDNTR  DLLFRDDTVCLAK  FDEFFSEGCAPGSK  TAGWNIPMGLLYNK  KPVEEYANCHLAR  EDPQTFYYAVAVVK  KCSTSSLLEACTFR  DCHLAQVPSHTVVAR  FDEFFSEGCAPGSKK  IECVSAETTEDCIAK  EGTCPEAPTDECKPVK  WCAVSEHEATKCQSFR  ADRDQYELLCLDNTR  NLNEKDYELLCLDGTR  EDLIWELLNQAQEHFGK  SDNCEDTPEAGYFAVAVVK  SAGWNIPIGLLYCDLPEPR  NLREGTCPEAPTDECKPVK  KPVDEYKDCHLAQVPSHTVVAR  LCMGSGLNLCEPNNKEGYYGYTGAFR  *AIAANEADAVTLDAGLVYDAYLAPNNLKPVVAEFYGSK* |
| 22 | 40 | -1.65 | (P02753) Plasma retinol-binding protein precursor (PRBP) (RBP) (PRO2222) | 23.02 | 637 | 576 | 11 | QEELCLAR  FSGTWYAMAK  DPNGLPPEAQK  YWGVASFLQK  QRQEELCLAR  LIVHNGYCDGR  LLNLDGTCADSYSFVFSR  LLNNWDVCADMVGTFTDTEDPAK  GNDDHWIVDTDYDTYAVQYSCR  *DPEGLFLQDNIVAEFSVDETGQMSATAK*  *KDPEGLFLQDNIVAEFSVDETGQMSATAK* |
| 23 | 41 | -1.73 | (P27169) Serum paraoxonase/arylesterase 1 (EC3.1.1.2) (EC 3.1.8.1) | 39.59 | 272 | 227 | 11 | LLIGTVFHK  NHQSSYQTR  IQNILTEEPK  YVYIAELLAHK  LIALTLLGMGLALFR  EVQPVELPNCNLVK  IFFYDSENPPASEVLR  VVAEGFDFANGINISPDGK  ILLMDLNEEDPTVLELGITGSK  *SLDFNTLVDNISVDPETGDLWVGCHPNGMK*  *FDVSSFNPHGISTFTDEDNAMYLLVVNHPDAK* |
| 24 | 42 | -1.79 | (P10909) Clusterin precursor (Complement-associated protein SP-40) | 52.46 | 179 | 157 | 8 | IDSLLENDR  RPHFFFPK  QTCMKFYAR  TLLSNLEEAKK  NPKFMETVAEK  ASSIIDELFQDR  QQTHMLDVMQDHFSR  EPQDTYHYLPFSLPHR |
| 25 | 43 | -1.85 | (P02768) Serum albumin precursor | 69.32 | 456 | 386 | 30 | CCKADDK  YLYEIAR  LCTVATLR  DDNPNLPR  LKCASLQK  FQNALLVR  LVNEVTEFAK  FKDLGEENFK  HPDYSVVLLLR  AVMDDFAAFVEK  RHPDYSVVLLLR  CCAAADPHECYAK  DVFLGMFLYEYAR  KVPQVSTPTLVEVSR  QEPERNECFLQHK  HPYFYAPELLFFAK  RHPYFYAPELLFFAK  HPYFYAPELLFFAKR  RPCFSALEVDETYVPK  VFDEFKPLVEEPQNLIK  EFNAETFTFHADICTLSEK  ALVLIAFAQYLQQCPFEDHVK  TCVADESAENCDKSLHTLFGDK  MPCAEDYLSVVLNQLCVLHEK  EFNAETFTFHADICTLSEKER  QNCELFEQLGEYKFQNALLVR  RMPCAEDYLSVVLNQLCVLHEK  LVRPEVDVMCTAFHDNEETFLK  RMPCAEDYLSVVLNQLCVLHEK  LVRPEVDVMCTAFHDNEETFLKK |
| 26 | 44 | -1.88 | (P00738) Haptoglobin precursor | 45.17 | 713 | 649 | 14 | DYAEVGR  GSFPWQAK  VGYVSGWGR  VTSIQDWVQK  DIAPTLTLYVGK  TEGDGVYTLNDK  SCAVAEYGVYVK  YVMLPVADQDQCIR  VSVNERVMPICLPSK  VVLHPNYSQVDIGLIK  VMPICLPSKDYAEVGR  SPVGVQPILNEHTFCAGMSK  YVMLPVADQDQCIRHYEGSTVPEK *YQEDTCYGDAGSAFAVHDLEEDTWYATGILSFDK* |
| 27 | 45 | -1.9 | (P02765) Alpha-2-HS-glycoprotein precursor (Fetuin-A) | 39.3 | 495 | 450 | 10 | QYGFCK  FSVVYAK  HTLNQIDEVK  CDSSPDSAEDVRK  EHAVEGDCDFQLLK  HTFMGVVSLGSPSGEVSHPR  HTFMGVVSLGSPSGEVSHPRK  AQLVPLPPSTYVEFTVSGTDCVAK  *QPNCDDPETEEAALVAIDYINQNLPWGYK*  *VWPQQPSGELFEIEIDTLETTCHVLDPTPVAR* |
| 28 | 46 | -1.94 | (P00738) Haptoglobin precursor | 45.17 | 586 | 511 | 14 | DYAEVGR  GSFPWQAK  VGYVSGWGR  VMPICLPSK  VTSIQDWVQK  DIAPTLTLYVGK  SCAVAEYGVYVK  YVMLPVADQDQCIR  AVGDKLPECEAVCGKPK  VMPICLPSKDYAEVGR  SPVGVQPILNEHTFCAGMSK  LPECEAVCGKPKNPANPVQR  FTDHLKYVMLPVADQDQCIR  YQEDTCYGDAGSAFAVH |
| 29 | 47 | -2.05 | (P00738) Haptoglobin precursor | 45.17 | 151 | 114 | 10 | KQWINK  NYYKLR  VMPICLPSK  YQCKNYYK  LPECEAVCGKPK  LRTEGDGVYTLNDK  YVMLPVADQDQCIR  LRTEGDGVYTLNNEK  VVLHPNYSQVDIGLIK  AVGDKLPECEAVCGKPK |
| 30 | 49 | -2.22 | (P00738) Haptoglobin precursor | 45.17 | 289 | 231 | 13 | DYAEVGR  KQWINK  QLVEIEK  NPANPVQR  GSFPWQAK  VGYVSGWGR  VMPICLPSK  VTSIQDWVQK  DIAPTLTLYVGK  SCAVAEYGVYVK  YVMLPVADQDQCIR  VMPICLPSKDYAEVGR  SPVGVQPILNEHTFCAGMSK |
| 31 | 50 | -2.4 | (P02768) Serum albumin precursor | 69.3 | 674 | 619 | 23 | YLYEIAR  FQNALLVR  QTALVELVK  CASLQKFGER  ECCEKPLLEK  HPDYSVVLLLR  AVMDDFAAFVEK  RHPDYSVVLLLR  CCAAADPHECYAK  VPQVSTPTLVEVSR  DVFLGMFLYEYAR  YICENQDSISSKLK  KVPQVSTPTLVEVSR  QNCELFEQLGEYK  QEPERNECFLQHK  RPCFSALEVDETYVPK  VPQVSTPTLVEVSRNLGK  SLHTLFGDKLCTVATLR  AAFTECCQAADKAACLLPK  VFDEFKPLVEEPQNLIK  EFNAETFTFHADICTLSEK  MPCAEDYLSVVLNQLCVLHEK  RMPCAEDYLSVVLNQLCVLHEK |
| 32 | 51 | -2.48 | (P01028) Complement C4 precursor (Contains: C4aanaphylatoxin; C4b) | 192.6 | 733 | 691 | 23 | ANSFLGEK  VQQPDCR  EFHLHLR  DKGQAGLQR  SCEQRAAR  LGQYASPTAK  GLCVATPVQLR  LPMMRSCEQR  GQIVFMNREPK  GSFEFPVGDAVSK  NPSRNNVPCSPK  SFFPENWLWR  VHYTVCIWRNGK  CCQDGVTRLPMMR  EELVYELNPLDHR  EPFLSCCQFAESLR  GCGEQTMIYLAPTLAASR  LLATLCSAEVCQCAEGKCPR  ALEILQEEDLIDEDDIPVR  GHLFLQTDQPIYNPGQRVR  EGAIHREELVYELNPLDHR  VTASDPLDTLGSEGALSPGGVASLLR  TLEIPGNSDPNMIPDGDFNSYVR |
| 33 | 52 | -2.49 | (P00738) Haptoglobin precursor | 45.17 | 713 | 649 | 14 | DYAEVGR  GSFPWQAK  VGYVSGWGR  VTSIQDWVQK  DIAPTLTLYVGK  TEGDGVYTLNDK  SCAVAEYGVYVK  YVMLPVADQDQCIR  VSVNERVMPICLPSK  VVLHPNYSQVDIGLIK  VMPICLPSKDYAEVGR  SPVGVQPILNEHTFCAGMSK  YVMLPVADQDQCIRHYEGSTVPEK  *YQEDTCYGDAGSAFAVHDLEEDTWYATGILSFDK* |
| 34 | 53 | -2.58 | (P00738) Haptoglobin precursor | 45.17 | 205 | 150 | 12 | DYAEVGR  GSFPWQAK  VGYVSGWGR  VMPICLPSK  VTSIQDWVQK  TEGDGVYTLNDK  SCAVAEYGVYVK  YVMLPVADQDQCIR  VSVNERVMPICLPSK  VVLHPNYSQVDIGLIK  SPVGVQPILNEHTFCAGMSK  ADQDQCIRHYEGSTVPEK |
| 35 | 54 | -2.59 | (P02768) Serum albumin precursor | 69.32 | 663 | 568 | 26 | CCKADDK  YLYEIAR  DDNPNLPR  FQNALLVR  CASLQKFGER  HPDYSVVLLLR  AVMDDFAAFVEK  RHPDYSVVLLLR  CCAAADPHECYAK  DVFLGMFLYEYAR  KVPQVSTPTLVEVSR  QNCELFEQLGEYK  QEPERNECFLQHK  HPYFYAPELLFFAK  RPCFSALEVDETYVPK  NECFLQHKDDNPNLPR  VFDEFKPLVEEPQNLIK  VHTECCHGDLLECADDR  EFNAETFTFHADICTLSEK  MPCAEDYLSVVLNQLCVLHEK  EFNAETFTFHADICTLSEKER  VHTECCHGDLLECADDRADLAK  LVRPEVDVMCTAFHDNEETFLK  RMPCAEDYLSVVLNQLCVLHEK  LVRPEVDVMCTAFHDNEETFLKK  CCTESLVNRRPCFSALEVDETYVPK |
| 36 | 55 | -2.84 | (P00738) Haptoglobin precursor | 45.17 | 239 | 192 | 11 | DYAEVGR  GSFPWQAK  ILGGHLDAK  VGYVSGWGR  VTSIQDWVQK  SCAVAEYGVYVK  YVMLPVADQDQCIR  VVLHPNYSQVDIGLIK  SPVGVQPILNEHTFCAGMSK  SCAVAEYGVYVKVTSIQDWVQK  *YQEDTCYGDAGSAFAVHDLEEDTWYATGILSFDK* |
| 37 | 56 | -2.95 | (P00738) Haptoglobin precursor | 45.17 | 217 | 150 | 13 | DYAEVGR  QLVEIEK  GSFPWQAK  ILGGHLDAK  VGYVSGWGR  VMPICLPSK  VTSIQDWVQK  DIAPTLTLYVGK  SCAVAEYGVYVK  YVMLPVADQDQCIR  VSVNERVMPICLPSK  VMPICLPSKDYAEVGR  SPVGVQPILNEHTFCAGMSK |
| 38 | 57 | -3.05 | (P00738) Haptoglobin precursor | 45.17 | 248 | 198 | 12 | NYYKLR  NPANPVQR  VMPICLPSK  YQCKNYYK  LPECEAVCGKPK  TEGDGVYTLNDKK  LRTEGDGVYTLNDK  YVMLPVADQDQCIR  LRTEGDGVYTLNNEK  AVGDKLPECEAVCGKPK  LPECEADDGCPKPPEIAHGYVEHSVR  *AVGDKLPECEADDGCPKPPEIAHGYVEHSVR* |
| 39 | 61 | 2.27 | (P01009) Alpha-1-antitrypsin precursor (Alpha-1 protease inhibitor) | 46.70 | 243 | 84 | 24 | SASLHLPK  RSASLHLPK  SVLGQLGITK  LSSWVLLMK  FLENEDRR  LSSWVLLMK  LSITGTYDLK  KLSSWVLLMK  LGMFNIQHCK  GKWERPFEVK  RLGMFNIQHCK  LGMFNIQHCKK  RLGMFNIQHCK  QINDYVEKGTQGK  ITPNLAEFAFSLYR  VFSNGADLSGVTEEAPLK  FNKPFVFLMIEQNTK  ELDRDTVFALVNYIFFK  LSITGTYDLKSVLGQLGITK  VFSNGADLSGVTEEAPLKLSK  KLYHSEAFTVNFGDTEEAK  LYHSEAFTVNFGDTEEAKK  TLNQPDSQLQLTTGNGLFLSEGLK  *TDTSHHDQDHPTFNKITPNLAEFAFSLYR* |
| 40 | 66 | 1.67 | (P01011) Alpha-1-antichymotrypsin precursor (ACT) | 47.62 | 224 | 74 | 21 | LINDYVK  ADLSGITGAR  EQLSLLDR  KLINDYVK  EIGELYLPK  NLAVSQVVHK  WRDSLEFR  ITLLSALVETR  EQLSLLDRFTEDAK  WEMPFDPQDTHQSR  MEEVEAMLLPETLKR  DSLEFREIGELYLPK  LYGSEAFATDFQDSAAAK  AVLDVFEEGTEASAATAVK  AKWEMPFDPQDTHQSR  RLYGSEAFATDFQDSAAAK  DLDSQTMMVLVNYIFFK  GTHVDLGLASANVDFAFSLYK  WVMVPMMSLHHLTIPYFR  DYNLNDILLQLGIEEAFTSK  FNRPFLMIIVPTDTQNIFFMSK |
| 41 | 67 | 1.45 | (P01009) Alpha-1-antitrypsin precursor (Alpha-1 protease inhibitor) | 46.70 | 383 | 307 | 16 | LSSWVLLMK  FLENEDRR  WERPFEVK  LGMFNIQHCK  GKWERPFEVK  ITPNLAEFAFSLYR  TDTSHHDQDHPTFNK  LQHLENELTHDIITK  VFSNGADLSGVTEEAPLK  FNKPFVFLMIEQNTK  DTEEEDFHVDQVTTVK  LYHSEAFTVNFGDTEEAK  ELDRDTVFALVNYIFFK  LYHSEAFTVNFGDTEEAKK  GTEAAGAMFLEAIPMSIPPEVK  TLNQPDSQLQLTTGNGLFLSEGLK |
| 42 | 68 | -1.28 | (P02787) Serotransferrin precursor (Transferrin) | 76.99 | 674 | 590 | 25 | ASYLDCIR  WCALSHHER  DSGFQMNQLR  SASDLTWDNLK  EFQLFSSPHGK  EGYYGYTGAFR  DYELLCLDGTR  CLVEKGDVAFVK  NPDPWAKNLNEK  MYLGYEYVTAIR  CSTSSLLEACTFR  DQYELLCLDNTR  FDEFFSEGCAPGSK  TAGWNIPMGLLYNK  KPVEEYANCHLAR  EDPQTFYYAVAVVK  DCHLAQVPSHTVVAR  FDEFFSEGCAPGSKK  WCAVSEHEATKCQSFR  ADRDQYELLCLDNTR  NLNEKDYELLCLDGTR  EDLIWELLNQAQEHFGK  SAGWNIPIGLLYCDLPEPR  DSSLCKLCMGSGLNLCEPNNK  KPVDEYKDCHLAQVPSHTVVAR |
| 43 | 71 | -1.87 | (P06727) Apolipoprotein A-IV precursor (Apo-AIV)(ApoA-IV) | 45.34 | 760 | 592 | 24 | ISASAEELR  QLTPYAQR  LAPLAEDVR  VNSFFSTFK  LTPYADEFK  LEPYADQLR  ALVQQMEQLR  LLPHANEVSQK  IDQTVEELRR  TQVNTQAEQLR  IDQNVEELKGR  LVPFATELHER  DKVNSFFSTFK  SLAPYAQDTQEK  RVEPYGENFNK  LGEVNTYAGDLQK  KLVPFATELHER  TQVNTQAEQLRR  LNHQLEGLTFQMK  SELTQQLNALFQDK  LGPHAGDVEGHLSFLEK  SLAELGGHLDQQVEEFR  ENADSLQASLRPHADELK  SLAELGGHLDQQVEEFRR |
| 44 | 72 | -1.97 | (P30626) Sorcin (22 kDa protein) (CP-22) (V19) | 21.66 | 27 | - | 6 | LSPQAVNSIAK  SGTVDPQELQK  QHFISFDTDR  LSPQAVNSIAKR  ITFDDYIACCVKLR  RDTAQQGVVNFPYDDFIQCVMSV |
| 45 | 73 | -2 | (P02766) Transthyretin precursor (Prealbumin) (TBPA) (TTR) (ATTR) | 15.9 | 88 | 76 | 3 | GSPAINVAVHVFR  ALGISPFHEHAEVVFTANDSGPR  TSESGELHGLTTEEEFVEGIYK |
| 46 | 76 | -2.35 | (P00738) Haptoglobin precursor | 45.17 | 457 | 396 | 13 | DYAEVGR  GSFPWQAK  VGYVSGWGR  VMPICLPSK  VTSIQDWVQK  DIAPTLTLYVGK  TEGDGVYTLNDK  SCAVAEYGVYVK  YVMLPVADQDQCIR  VVLHPNYSQVDIGLIK  VMPICLPSKDYAEVGR  SPVGVQPILNEHTFCAGMSK  *YQEDTCYGDAGSAFAVHDLEEDTWYATGILSFDK* |
| 47 | 78 | -2.46 | (P02768) Serum albumin precursor | 69.3 | 1410 | 1296 | 26 | YLYEIAR  LCTVATLR  DDNPNLPR  FQNALLVR  NECFLQHK  CCTESLVNR  LVNEVTEFAK  ECCEKPLLEK  HPDYSVVLLLR  AVMDDFAAFVEK  AAFTECCQAADK  RHPDYSVVLLLR  CCAAADPHECYAK  VPQVSTPTLVEVSR  DVFLGMFLYEYAR  KVPQVSTPTLVEVSR  QNCELFEQLGEYK  HPYFYAPELLFFAK  RPCFSALEVDETYVPK  VHTECCHGDLLECADDR  VFDEFKPLVEEPQNLIK  EFNAETFTFHADICTLSEK  ALVLIAFAQYLQQCPFEDHVK  MPCAEDYLSVVLNQLCVLHEK  LVRPEVDVMCTAFHDNEETFLK  SHCIAEVENDEMPADLPSLAADFVESK |
| 48 | 79 | -3.25 | (P00738) Haptoglobin precursor | 45.17 | 306 | 262 | 9 | DYAEVGR  NYYKLR  GSFPWQAK  VGYVSGWGR  VMPICLPSK  YQCKNYYK  VTSIQDWVQK  SCAVAEYGVYVK  YVMLPVADQDQCIR |
| 49 | 83 | 3.55 | (Q9H293) Interleukin-17E precursor (I17E)(Interleukin-25) (IL-25) | 20.31 | 28 | 19 | 4 | ASEDGPLNSR  RPCHGEKGTHK  CLCPHCVSLQTGSHMDPR  GNSELLYHNQTVFYRRPCHGEK |
| 50 | 97 | 1.58 | (P02790) Hemopexin precursor (Beta-1B-glycoprotein) | 51.64 | 596 | 496 | 21 | LHIMAGR  LWWLDLK  GEVPPRYPR  RLWWLDLK  DYFMPCPGR  VWVYPPEKK  FDPVRGEVPPR  GDKVWVYPPEK  SWPAVGNCSSALR  YYCFQGNQFLR  EWFWDLATGTMK  GECQAEGVLFFQGDR  LYLVQGTQVYVFLTK  SGAQATWTELPWPHEK  SWPAVGNCSSALRWLGR  LLQDEFPGIPSPLDAAVECHR  EVGTPHGIILDSVDAAFICPGSSR  *CSPHLVLSALTSDNHGATYAFSGTHYWR*  *GECQAEGVLFFQGDREWFWDLATGTMK*  *DGWHSWPIAHQWPQGPSAVDAAFSWEEK*  *SLGPNSCSANGPGLYLIHGPNLYCYSDVEK* |
| 51 | 99 | 1.5 | (P01876) Ig alpha-1 chain C region | 37.63 | 491 | 432 | 14 | VAAEDWK  YLTWASR  SAVQGPPER  EKYLTWASR  WLQGSQELPR  TFTCTAAYPESK  DASGVTFTWTPSSGK  QEPSQGTTTFAVTSILR  GDTFSCMVGHEALPLAFTQK  TFTCTAAYPESKTPLTATLSK  KGDTFSCMVGHEALPLAFTQK  DLCGCYSVSSVLPGCAEPWNHGK  *NFPPSQDASGDLYTTSSQLTLPATQCLAGK*  *SGNTFRPEVHLLPPPSEELALNELVTLTCLAR* |
| 52 | 100 | 1.5 | (P01876) Ig alpha-1 chain C region | 37.63 | 588 | 532 | 12 | VAAEDWK  YLTWASR  SAVQGPPER  WLQGSQELPR  TFTCTAAYPESK  DASGVTFTWTPSSGK  QEPSQGTTTFAVTSILR  GDTFSCMVGHEALPLAFTQK  KGDTFSCMVGHEALPLAFTQK  DLCGCYSVSSVLPGCAEPWNHGK  *NFPPSQDASGDLYTTSSQLTLPATQCLAGK*  *SGNTFRPEVHLLPPPSEELALNELVTLTCLAR* |
| 53 | 102 | 1.48 | (Q9HC96) Calpain 10 (EC 3.4.22.) (Calcium-activated neutral proteinase 10) | 74.89 | 29 | - | 12 | IWQFGR  VGQTAGGSR  AGRGATPAR  IQNPWGR  LHAADWAGR  WEHRTCR  TCRQLLHLK  LCFSRCQR  DQCLISCCVLSPR  HYQAVGLHLWKVEK  DQCLISCCVLSPRAGAR  QLLHLKDQCLISCCVLSPR |
| 54 | 104 | -1.52 | (P14136) Glial fibrillary acidic protein, astrocyte (GFAP) | 49.84 | 38 | 23 | 7 | HEANDYRR  LEAENNLAAYR  SKFADLTDAAAR  LALDIEIATYR  GTNESLERQMR  LRLDQLTANSAR  ITIPVQTFSNLQIR |
| 55 | 105 | -1.53 | (P01028) Complement C4 precursor (Contains: C4a anaphylatoxin; C4b) | 192.6 | 201 | 170 | 19 | EDSRAAFR  QGSFQGGFR  GLCVATPVQLR  KADGSYAAWLSR  GQIVFMNREPK  SFFPENWLWR  VHYTVCIWRNGK  CCQDGVTRLPMMR  AINEKLGQYASPTAK  EELVYELNPLDHR  EPFLSCCQFAESLR  GCGEQTMIYLAPTLAASR  LLATLCSAEVCQCAEGKCPR  ALEILQEEDLIDEDDIPVR  EGAIHREELVYELNPLDHR  VTASDPLDTLGSEGALSPGGVASLLR  TLEIPGNSDPNMIPDGDFNSYVR  STQDTVIALDALSAYWIASHTTEER  LQETSNWLLSQQQADGSFQDPCPVLDR |
| 56 | 107 | -1.69 | (P10909) Clusterin precursor (Complement-associated protein SP-40) | 52.46 | 210 | 201 | 7 | FMETVAEK  ELDESLQVAER  ASSIIDELFQDR  EILSVDCSTNNPSQAK  LFDSDPITVTVPVEVSR  EPQDTYHYLPFSLPHR  CREILSVDCSTNNPSQAK |
| 57 | 109 | -2.08 | P06727) Apolipoprotein A-IV precursor (Apo-AIV)(ApoA-IV) | 45.34 | 128 | - | 20 | MERVLR  GNTEGLQK  ISASAEELR  QLTPYAQR  LAPLAEDVR  LTPYADEFK  LEPYADQLR  ALVQQMEQLR  LLPHANEVSQK  IDQTVEELRR  LVPFATELHER  RVEPYGENFNK  KLVPFATELHER  LNHQLEGLTFQMK  SELTQQLNALFQDK  LGPHAGDVEGHLSFLEK  SLAELGGHLDQQVEEFR  ENADSLQASLRPHADELK  QKLGPHAGDVEGHLSFLEK  SLAELGGHLDQQVEEFRR |
| 58 | 116 | 1.58 | (Q9Y376) Calcium binding protein 39 (Mo25 protein) (CGI-66) | 39.84 | 36 | - | 9 | DLLTRHK  LMMNLLRDK  LLHSENYVTK  NLKESMAVLEK  DVAQIFNNILR  GYESPEIALNCGIMLR  YISKPENLKLMMNLLR  YVEMSTFDIASDAFATFK  GYESPEIALNCGIMLRECIR |
| 59 | 117 | 1.46 | (P02649) Apolipoprotein E precursor (Apo-E) | 36.13 | 1100 | 955 | 21 | DADDLQK  AQAWGER  EQVAEVR  FWDYLR  WELALGR  ELQAAQAR  QWAGLVEK  LAVYQAGAR  LGPLVEQGR  LQAEAFQAR  LEEQAQQIR  LGADMEDVCGR  QQTEWQSGQR  AKLEEQAQQIR  AATVGSLAGQPLQER  SWFEPLVEDMQR  VEQAVETEPEPELR  GEVQAMLGQSTEELR  SELEEQLTPVAEETR  AYKSELEEQLTPVAEETR  WVQTLSEQVQEELLSSQVTQELR |
| 60 | 118 | 1.02 | (P04217) Alpha-1B-glycoprotein precursor (Alpha-1-BN glycoprotein) | 54.3 | 900 | 821 | 16 | GVTFLLR  LLELTGPK  CLAPLEGAR  ATWSGAVLAGR  LETPDFQLFK  SGLSTGWTQLSK  HQFLLTGDTQGR  CEGPIPDVTFELLR  NGVAQEPVHLDSPAIK  LELHVDGPPPRPQLR  VTLTCVAPLSGVDFQLR  IFFHLNAVALGDGGHYTCR  SLPAPWLSMAPVSWITPGLK  TPGAAANLELIFVGPQHAGNYR  SWVPHTFESELSDPVELLVAES  *LHDNQNGWSGDSAPVELILSDETLPAPEFSPEPESGR* |
| 61 | 119 | -1.21 | (O75636) Ficolin 3 precursor (Collagen/fibrinogen domain-containing protein 3) | 32.9 | 515 | 463 | 10 | VRMMLR  YGIDWASGR  QDGSVDFFR  TFAHYATFR  GEPGDPVNLLR  RQDGSVDFFR  LLGEVDHYQLALGK  ALPVFCDMDTEGGGWLVFQR  ELLSQGATLSGWYHLCLPEGR  *AGFGNQESEFWLGNENLHQLTLQGNWELR* |
| 62 | 120 | -1.4 | (P00751) Complement factor B precursor (EC 3.4.21.47) (C3/C5 convertase) (Properdin factor B) | 85.4 | 717 | 603 | 26 | ISVIRPSK  STGSWSTLK  VASYGVKPR  DISEVVTPR  EELLPAQDIK  ALFVSEEEKK  VKDISEVVTPR  YGLVTYATYPK  LPPTTTCQQQK  EKLQDEDLGFL  LEDSVTYHCSR  QLNEINYEDHK  DFHINLFQVLPWLK  FIQVGVISWGVVDVCK  EAGIPEFYDYDVALIK  FLCTGGVSPYADPNTCR  KEAGIPEFYDYDVALIK  SRFIQVGVISWGVVDVCK  DLEIEVVLFHPNYNINGK  YGQTIRPICLPCTEGTTR  AIHCPRPHDFENGEYWPR  LPPTTTCQQQKEELLPAQDIK  WSGQTAICDNGAGYCSNPGIPIGTR  LLQEGQALEYVCPSGFYPYPVQTR  EDYLDVYVFGVGPLVNQVNINALASK  *GHESCMGAVVSEYFVLTAAHCFTVDDKEHSIK* |
| 63 | 121 | -2.1 | (P02647) Apolipoprotein A-I precursor (Apo-AI) | 30.75 | 486 | 351 | 20 | QKVEPLR  AELQEGAR  LHELQEK  AKPALEDLR  LSPLGEEMR  QKLHELQEK  QGLLPVLESFK  DLATVYVDVLK  VQPYLDDFQK  WQEEMELYR  THLAPYSDELR  LSPLGEEMRDR  VQPYLDDFQKK  VSFLSALEEYTK  DYVSQFEGSALGK  KWQEEMELYR  VEPLRAELQEGAR  LLDNWDSVTSTFSK  DSGRDYVSQFEGSALGK  LREQLGPVTQEFWDNLEK |

^$^ [Analysis Type: Combined (MS+MS/MS); Database: SwissProt; Taxonomy: All entries]

# Alterations in protein expression levels in *falciparum* malaria (FM) were measured using healthy subjects as controls

* indicates the continuation of peptide sequence in the next line
