# Supplementary material for: Proteomic Investigation of Falciparum and Vivax Malaria for Identification of Surrogate Protein Markers
Source: PLoS One. 2012 Aug 9;7(8):e41751. doi: 10.1371/journal.pone.0041751 (PMC3415403; doi:10.1371/journal.pone.0041751)
Supplement: Table S3 — Comparison of differentially expressed serum proteins in falciparum and vivax malaria. (DOC) [file pone.0041751.s012.doc]

**Table S3.** Comparison of differentially expressed serum proteins in *falciparum* and *vivax* malaria

**Table S3.1.** Differentially expressed serum proteins common in both *falciparum* and *vivax* malaria #

| **Sl No.** | **Name of the proteins** | UniProt **accession number** | **Gene**  **symbol** | **Fold change in FM** | **Fold change in VM** |
| --- | --- | --- | --- | --- | --- |
| 1 | Serum amyloid A (SAA) | P02735 | SAA1 & SAA2 | 39.77 | 17.7 |
| 2 | Interleukin-17E precursor (IL-17E) | Q9H293 | IL25 | 3.55 | 1.38 |
| 3 | Alpha-1-antitrypsin precursor (Alpha-1 protease inhibitor) | P01009 | SERPINA1 | 2.5 | 1.97 |
| 4 | Leucine-rich alpha-2-glycoprotein precursor (LRG) | P02750 | LRG1 | 2.5 | 4.25 |
| 5 | Vitronectin precursor (Serum spreading factor) (S-protein) | P04004 | VTN | 2.4 | 2.14 |
| 6 | Ig kappa chain C region | P01834 | IGKC | 2.17 | 1.71 |
| 7 | Apolipoprotein E precursor | P02647 | APOE | 1.82 | 5.72 |
| 8 | Hemopexin precursor (Beta-1B-glycoprotein) | P02790 | HPX | 1.58 | 1.51 |
| 9 | Ig mu chain C region | P01871 | IGHM | 1.56 | 1.43 |
| 10 | Alpha-1-antichymotrypsin precursor (ACT) | P01011 | SERPINA3 | 1.5 | 2.94 |
| 11 | Ig alpha-1 chain C region | P01876 | IGHA1 | 1.5 | 1.73 |
| 12 | Haptoglobin precursor | P00738 | HPR & HP | -2.5 | -1.85 |
| 13 | Serum albumin precursor | P02768 | ALB | -2.4 | -2.09 |
| 14 | Alpha-2-HS-glycoprotein precursor (Fetuin-A)(Alpha-2-Z-globulin) | P02765 | AHSG | -1.9 | 2.05 |
| 15 | Clusterin precursor (Complement-associated protein SP-40) | P10909 | CLU | -1.65 | -1.44 |
| 16 | Plasma retinol-binding protein precursor (PRBP) | P02753 | RBP4 | -1.65 | -1.46 |
| 17 | Ficolin 3 precursor (Collagen/fibrinogen domain-containing protein 3) | O75636 | FCN3 | -1.45 | -1.96 |
| 18 | Serotransferrin precursor (Transferrin) | P02787 | TF | -1.3 | 1.63 |
| 19 | Apolipoprotein A-I precursor (Apo-AI) | P02647 | APOA1 | -1.28 | -1.54 |

**#** Alterations in protein expression levels in *falciparum* and *vivax* malaria were measured using healthy subjects as controls

**Table S3.2.** Serum proteins differentially expressed only in *falciparum* malaria (not in *vivax* malaria) #

| **Sl No.** | **Name of the proteins** | UniProt **Accession number** | **Gene**  **symbol** | **Fold change** |
| --- | --- | --- | --- | --- |
| 1 | Alpha-1B-glycoprotein precursor (Alpha-1-BN glycoprotein) | P04217 | A1BG | 1.3 |
| 2 | Calcium binding protein 39 (Mo25 protein) (CGI-66) | Q9Y376 | CAB39 | 1.58 |
| 3 | Calpain 10 (EC 3.4.22.-) (Calcium-activated neutral proteinase 10) | Q9HC96 | CAPN10 | 1.48 |
| 4 | Regulator of G-protein signaling 7 (RGS7) | P49802 | RGS7 | -1.36 |
| 5 | Transthyretin precursor (Prealbumin) (TBPA) (TTR) (ATTR) | P02766 | TTR | -2 |
| 6 | Sorcin (22 kDa protein) (CP-22) (V19) | P30626 | SRI | -1.97 |
| 7 | Complement C4 precursor [Contains: C4a anaphylatoxin; C4b] | P01028 | C4A & C4B | -2.48 |
| 8 | Complement factor B precursor (EC 3.4.21.47) (C3/C5 convertase) (Properdin factor B) | P00751 | CFB | -2.1 |
| 9 | Apolipoprotein A-IV precursor (Apo-AIV)(ApoA-IV) | P06727 | APOA4 | -2 |
| 10 | Serum paraoxonase/arylesterase 1 (EC3.1.1.2) (EC 3.1.8.1) (PON 1) (Serum aryldialkylphosp | P27169 | PON1 | -1.73 |
| 11 | Glial fibrillary acidic protein, astrocyte (GFAP) | P14136 | GFAP | -1.52 |

**#** Alterations in protein expression levels in *falciparum* malaria were measured using healthy subjects as controls

**Table S3.3.** Serum proteins differentially expressed only in *vivax* malaria (not in *falciparum* malaria) #

| **Sl No.** | **Name of the proteins** | UniProt **accession number** | **Gene**  **symbol** | **Fold change** |
| --- | --- | --- | --- | --- |
| 1 | Ubiquitin-like protein SMT3A precursor | P55854 | SUMO2 & SUMO3 | -3 |
| 2 | SSX2 protein (Synovial sarcoma, X breakpoint 2) (SSX) (HOM-MEL-40) | Q16385 | SSX2 | -5 |
| 3 | Alpha-2-macroglobulin precursor (α-2-M) | P01023 | A2M | -1.3 |
| 4 | Complement C3 precursor | P01024 | C3 | -1.34 |
| 5 | Ceruloplasmin precursor | P00450 | CP | 2.73 |
| 6 | Pigment epithelium-derived factor precursor | P36955 | SERPINF1 | 1.42 |
| 7 | Vitamin D-binding protein precursor (DBP) | P02774 | GC | 1.46 |
| 8 | Fibrinogen beta chain precursor | P02675 | FGB | 1.51 |
| 9 | AMBP protein precursor [Contains:Alpha-1-microglobulin (Protein HC) | P02760 | AMBP | 1.79 |
| 10 | Serum amyloid P-component precursor (SAP) | P02743 | APCS | 1.98 |
| 11 | Interleukin 1 family member 7 precursor (IL-1F7) | Q9NZH6 | IL1F7 | 1.5 |
| 12 | Glutamate--cysteine ligase (EC 6.3.2.2) | Q97IV1 | GCLM | 1.88 |

**#** Alterations in protein expression levels in *vivax* malaria were measured using healthy subjects as controls

**Table S3.4.** Differential expression of serum proteins in *falciparum* malaria compared to *vivax* malaria #

| **Sl No.** | **Name of the proteins** | UniProt **accession number** | **Gene**  **symbol** | **Fold change** |
| --- | --- | --- | --- | --- |
| 1 | Interleukin-17E precursor (IL-17E) | Q9H293 | IL25 | 2.57 |
| 2 | Serum amyloid A protein precursor (SAA) | P02735 | SAA1 & SAA2 | 2.25 |
| 3 | Ficolin 3 precursor (Collagen/fibrinogen domain-containing protein 3) | O75636 | FCN3 | 1.35 |
| 4 | Alpha-1-antitrypsin precursor (Alpha-1 protease inhibitor) | P01009 | SERPINA1 | 1.27 |
| 5 | Ig kappa chain C region | P01834 | IGKC | 1.27 |
| 6 | Alpha-2-HS-glycoprotein precursor (Fetuin-A)(Alpha-2-Z-globulin) | P02765 | AHSG | -3.89 |
| 7 | Apolipoprotein E precursor | P02647 | APOE | -3.14 |
| 8 | Serotransferrin precursor (Transferrin) | P02787 | TF | -2.12 |
| 9 | Alpha-1-antichymotrypsin precursor (ACT) | P01011 | SERPINA3 | -1.96 |
| 10 | Leucine-rich alpha-2-glycoprotein precursor (LRG) | P02750 | LRG1 | -1.7 |
| 11 | AMBP protein precursor [Contains:Alpha-1-microglobulin (Protein HC) | P02760 | AMBP | -1.6 |
| 12 | Vitamin D-binding protein precursor (DBP) | P02774 | GC | -1.52 |
| 13 | Haptoglobin precursor | P00738 | HPR & HP | -1.35 |

**#** Alterations in protein expression levels in *falciparum* malaria were measured compared to the expression levels in *vivax* malaria
