# Supplementary material for: Proteomic Investigation of Falciparum and Vivax Malaria for Identification of Surrogate Protein Markers
Source: PLoS One. 2012 Aug 9;7(8):e41751. doi: 10.1371/journal.pone.0041751 (PMC3415403; doi:10.1371/journal.pone.0041751)
Supplement: Table S4 — Comparative serum proteome analysis of malaria (FM and VM) and febrile control (Leptospirosis) by 2D-DIGE. (DOC) [file pone.0041751.s013.doc]

**Table S4. Comparative serum proteome analysis of malaria (FM and VM) and febrile control (Leptospirosis) by 2D-DIGE ^**

| **S. No.** | **Name of the proteins** | **Accession No.** | **Fold change** | | |
| --- | --- | --- | --- | --- | --- |
|  |  |  | **FM** | **VM** | **FC^$^** |
| 1 | Alpha-1-antichymotrypsin precursor (ACT) | P01011 | 1.5 | 2.94 | 1.40-1.68 |
| 2 | Alpha-1-antitrypsin precursor (Alpha-1 protease inhibitor) | P01009 | 2.5 | 1.97 | 1.81-2.29 |
| 3 | Alpha-1B-glycoprotein precursor (Alpha-1-BN glycoprotein) | P04217 | 1.3 | * | 1.30-1.89 |
| 4 | Alpha-2-HS-glycoprotein precursor (Fetuin-A)(Alpha-2-Z-globulin) | P02765 | -1.9 | 2.05 | -1.65 |
| 5 | Alpha-2-macroglobulin precursor (α-2-M) | P01023 | * | -1.3 | * |
| 6 | AMBP protein precursor [Contains:Alpha-1-microglobulin (Protein HC) | P02760 | * | 1.79 | * |
| 7 | Apolipoprotein A-I precursor (Apo-AI) (ApoA-I) | P02647 | -1.28 | -1.54 | -(3.01- 2.83) |
| 8 | Apolipoprotein A-IV precursor (Apo-AIV)(ApoA-IV) | P06727 | -2 | * | -1.91 |
| 9 | Apolipoprotein E precursor | P02647 | 1.82 | 5.72 | 1.43 |
| 10 | Calcium binding protein 39 (Mo25 protein) (CGI-66) | Q9Y376 | 1.58 | * | * |
| 11 | Calpain 10 (EC 3.4.22.-) (Calcium-activated neutral proteinase 10) | Q9HC96 | 1.48 | * | * |
| 12 | Ceruloplasmin precursor | P00450 | * | 2.73 | 1.19-1.59 |
| 13 | Clusterin precursor (Complement-associated protein SP-40,40) | P10909 | -1.65 | -1.44 | -2.16 |
| 14 | Complement C3 precursor | P01024 | * | -1.34 | -2.21 |
| 15 | Complement C4 precursor [Contains: C4a anaphylatoxin; C4b] | P01028 | -2.48 | * | * |
| 16 | Complement factor B precursor (EC 3.4.21.47) (C3/C5 convertase) | P00751 | -2.1 | * | 1.17 |
| 17 | Fibrinogen beta chain precursor | P02675 | * | 1.51 | * |
| 18 | Ficolin 3 precursor (Collagen/fibrinogen domain-containing protein 3)# | O75636 | -1.45 | -1.96 | * |
| 19 | Glial fibrillary acidic protein, astrocyte (GFAP) | P14136 | -1.52 | * | * |
| 20 | Glutamate--cysteine ligase (EC 6.3.2.2) | Q97IV1 | * | 1.88 | * |
| 21 | Haptoglobin precursor | P00738 | -2.5 | -1.85 | * |
| 22 | Hemopexin precursor (Beta-1B-glycoprotein)# | P02790 | 1.58 | 1.51 | * |
| 23 | Ig alpha-1 chain C region | P01876 | 1.5 | 1.73 | 1.15 |
| 24 | Ig kappa chain C region | P01834 | 2.17 | 1.71 | - (1.2-1.35) |
| 25 | Ig mu chain C region | P01871 | 1.56 | 1.43 | - (1.73-2.28) |
| 26 | Interleukin 1 family member 7 precursor (IL-1F7) | Q9NZH6 | * | 1.5 | * |
| 27 | Interleukin-17E precursor (IL-17E)# | Q9H293 | 3.55 | 1.38 | * |
| 28 | Leucine-rich alpha-2-glycoprotein precursor (LRG) | P02750 | 2.5 | 4.25 | 1.54-2.05 |
| 29 | Pigment epithelium-derived factor precursor | P36955 | * | 1.42 | * |
| 30 | Plasma retinol-binding protein precursor (PRBP)# | P02753 | -1.65 | -1.46 | * |
| 31 | Regulator of G-protein signaling 7 (RGS7) | P49802 | -1.36 | * | -1.62 |
| 32 | Serotransferrin precursor (Transferrin)# | P02787 | -1.3 | 1.63 | * |
| 33 | Serum albumin precursor | P02768 | -2.4 | -2.09 | - (1.76-3) |
| 34 | Serum amyloid A (SAA)# | P02735 | 39.77 | 17.7 | * |
| 35 | Serum amyloid P-component precursor (SAP) | P02743 | * | 1.98 | * |
| 36 | Serum paraoxonase/arylesterase 1 (EC3.1.1.2) (EC 3.1.8.1) (PON 1) | P27169 | -1.73 | * | * |
| 37 | Sorcin (22 kDa protein) (CP-22) (V19) | P30626 | -1.97 | * | * |
| 38 | SSX2 protein (Synovial sarcoma, X breakpoint 2) (SSX) | Q16385 | * | -5 | * |
| 39 | Transthyretin precursor (Prealbumin) (TBPA) (TTR) (ATTR) | P02766 | -2 | * | * |
| 40 | Ubiquitin-like protein SMT3A precursor | P55854 | * | -3 | * |
| 41 | Vitamin D-binding protein precursor (DBP) | P02774 | * | 1.46 | * |
| 42 | Vitronectin precursor (Serum spreading factor) (S-protein)# | P04004 | 2.4 | 2.14 | 2.26 |

^$^ Patients suffering from leptospirosis were selected as febrile controls for this study

**^** Alterations in protein expression levels in *falciparum* and *vivax* malaria and leptospirosis were measured using healthy subjects as controls

* No differential expression (statistically not significant), # Differential expression observed in malaria (both FM and VM) but not in FC (Leptospirosis)
