# Supplementary material for: Proteomic Investigation of Falciparum and Vivax Malaria for Identification of Surrogate Protein Markers
Source: PLoS One. 2012 Aug 9;7(8):e41751. doi: 10.1371/journal.pone.0041751 (PMC3415403; doi:10.1371/journal.pone.0041751)
Supplement: Table S5 — Details of the pathways associated with the differentially expressed proteins identified in falciparum malaria defined by IPA, PANTHER and DAVID analysis. (DOC) [file pone.0041751.s014.doc]

**Table S5.** Details of the pathways associated with the differentially expressed proteins identified in *falciparum* malaria defined by IPA, PANTHER and DAVID analysis

**Table S5.1.** Interaction networks defined by Ingenuity Pathways Analysis

| **ID** | **Molecules in network** | **Score** | **Focus molecules in**  **network**  **(in bold)** | **Top Functions** |
| --- | --- | --- | --- | --- |
| 1 | **A1BG**, **AQP3**, **ALB**, AQP3, CALR, CAV1, Ces2e, **CFB**, COMMD5, COMMD9, CREB-NFkB, CTAG1B (includes others), DEFB103A/DEFB103B, FCAMR, **FCN3**, GDF15, GNAQ, HNF4A, Igh-2, **IGHA1**, **IGHM**, **IGKC**, NFKB1, NFkB (complex), NfkB-Nfkbia,NFkB-TBP, **RBP4**, RBP5, **RGS7**, **SAA1**, SAA2, **SERPINA1**, SOAT1, **TF**, **TTR** | 35 | 14 | Cell signaling, Molecular transport, Vitamin and mineral metabolism |
| 2 | ANGPTL4, **APOA4**, **APOA1**, APOC1, APOC2, **APOE**, C9, cholesterol sulfate, chondroitin sulfate, **CLU**, Collagen type III, FN1, GPIIB-IIIA, GPLD1, Hemoglobin, **HP**, **HPX**, Integrin alpha V beta 3, ITGA8, ITGB6, ITGB8, L-alpha-palmitoyloleolyl-phosphatidylcholine, LACRT, lipid, **LRG1**, MMP12, MMP25, PLTP, **PON1**, SAA, **SERPINA3**, VLDLR, **VTN**, VTNR, ZNF202 | 23 | 10 | Lipid metabolism, Molecular transport, Small molecule Biochemistry |

Bold candidates are differentially expressed proteins in *falciparum* malaria identified in this study

**Table S5.2.** Pathways obtained from IPA functional analysis

| **Sl No.** | **Ingenuity canonical pathways** | **-log(p-value)** | **Ratio** | **Molecules** |
| --- | --- | --- | --- | --- |
| 1 | Acute Phase Response Signaling | 1.8E01 | 6.74E-02 | TTR, ALB, HPX, HP, TF, APOA1, AHSG, CFB, SERPINA3, SERPINA1, SAA1, RBP4 |
| 2 | Primary Immunodeficiency Signaling | 4.29E00 | 4.76E-^02^ | IGKC, IGHM, IGHA1 |
| 3 | LXR/RXR Activation | 3.74E00 | 3.23E-^02^ | APOE, APOA4, APOA1 |
| 4 | FXR/RXR Activation | 3.52E00 | 2.97E-^02^ | APOE, PON1, APOA1 |
| 5 | B Cell Development | 2.82E00 | 5.41E-02 | IGKC, IGHM |
| 6 | Complement System | 1.34E00 | 2.86E-02 | CFB |
| 7 | Coagulation System | 1.33E00 | 2.63E-02 | SERPINA1 |
| 8 | Communication between Innate and Adaptive Immune Cells | 9.29E-01 | 9.17E-03 | IGHA1 |
| 9 | cAMP-mediated signaling | 5.88E-01 | 4.59E-03 | RGS7 |
| 10 | G-Protein Coupled Receptor Signaling | 2.87E-01 | 1.89E-03 | RGS7 |

**Table S5.3.** Pathways obtained from PANTHER functional analysis

| **Sl No.** | **Pathways** | **%** | **Proteins involved** | **Protein**  **ID** | **GO molecular function** | **GO biological process** | **FM protein IDs (expected)** | **FM protein IDs (over/ under)** | ***p*-value** |
| --- | --- | --- | --- | --- | --- | --- | --- | --- | --- |
| 1 | Blood coagulation | 20 | 1 | P01009 | ● protein binding  ● peptidase inhibitor activity | protein metabolic  process | 0.07 | + | 7.21E-02 |
| 2 | Heterotrimeric G-protein signaling pathway-Gq alpha and Go alpha mediated pathway | 20 | 1 | P49802 | ● protein binding  ● small GTP ase regulator activity | ● cell surface receptor linked signal transduction ● signal transduction● dorsal/ventral axis specification | 0.21 | + | 1.89E-01 |
| 3 | Interleukin signaling pathway | 20 | 1 | Q9H293 | receptor binding | ● immune system process ● cell surface receptor linked signal transduction ● intra cellular signaling cascade ●cell-cell signaling ● signal transduction ●cell-cell signaling | 0.25 | + | 2.23E-01 |
| 4 | Heterotrimeric G-protein signaling pathway-Gi alpha and Gs alpha mediated pathway | 20 | 1 | P49802 | ● protein binding  ● small GTP ase regulator activity | ● cell surface receptor linked signal transduction ● signal transduction  ● dorsal/ventral axis specification | 0.26 | + | 2.29E-01 |
| 5 | Inflammation mediated by chemokine and cytokine signaling pathway | 20 | 1 | Q9H293 | receptor binding | ● immune system process ● cell surface receptor linked signal transduction ● intra cellular signaling cascade ●cell-cell signaling ● signal transduction ●cell-cell signaling | 0.44 | + | 3.58E-01 |

**Table S5.4.** Pathways obtained from DAVID analysis

| **Category** | **Term** | **Count** | **%** | ***p* value** | **Genes** | **List total** | **Pop hits** | **Pop total** | **Fold enrich-ment** | **Bonferroni** | **Benjamini** | **FDR** |
| --- | --- | --- | --- | --- | --- | --- | --- | --- | --- | --- | --- | --- |
| **KEGG- PATHWAY** | hsa04610:Complement and coagulation cascades | 4 | 12.12 | 1.28E-^04^ | P00751  P01009 P0C0L4  P0C0L5 | 9 | 69 | 5085 | 32.75 | 0.001020 | 0.001020 | 0.069823 |
