# Supplementary material for: Proteomic Investigation of Falciparum and Vivax Malaria for Identification of Surrogate Protein Markers
Source: PLoS One. 2012 Aug 9;7(8):e41751. doi: 10.1371/journal.pone.0041751 (PMC3415403; doi:10.1371/journal.pone.0041751)
Supplement: Table S6 — Gene Ontology (GO) terms for molecular functions, cellular components and biological processes associated with the differentially expressed proteins identified in falciparum malaria. (DOC) [file pone.0041751.s015.doc]

**Table S6.** Gene Ontology (GO) terms for molecular functions, cellular components and biological processes associated with the differentially expressed proteins identified in *falciparum* malaria #

| **Sl. No.** | **GO ID** | **GO ACCESSION** | **GO Term** | ***p*-value** | **corrected *p*-value** | **Count in selection*** | | **% Count in selection** • | **Count in total ^** | **% Count in total ^$^** |
| --- | --- | --- | --- | --- | --- | --- | --- | --- | --- | --- |
| **A. Molecular Function** | | | | | | | | | | |
| 1 | 3876 | GO:0005515| GO:0045308 | protein binding | 1.72E-04 | 0.013016 | 22 | 70.96774 | | 6772 | 37.66826 |
| 2 | 19470 | GO:0043167 | ion binding | 0.541288 | 1 | 7 | 22.58065 | | 3963 | 22.04361 |
| 3 | 5809 | GO:0008289 | lipid binding | 1.68E-04 | 0.012871 | 6 | 19.35484 | | 492 | 2.736678 |
| 4 | 3464 | GO:0004857 | enzyme inhibitor activity | 1.63E-04 | 0.012725 | 5 | 16.12903 | | 306 | 1.70208 |
| 5 | 5624 | GO:0008047 | enzyme activator activity | 5.48E-04 | 0.034753 | 5 | 16.12903 | | 398 | 2.213817 |
| 6 | 9836 | GO:0016787 | hydrolase activity | 0.386106 | 1 | 5 | 16.12903 | | 2362 | 13.13828 |
| 7 | 28100 | GO:0061134 | peptidase regulator activity | 2.12E-05 | 0.002546 | 5 | 16.12903 | | 199 | 1.106908 |
| 8 | 2673 | GO:0003823 | antigen binding | 1.72E-04 | 0.013016 | 3 | 9.67742 | | 63 | 0.350428 |
| 9 | 12574 | GO:0022892 | substrate-specific transporter activity | 0.239932 | 1 | 3 | 9.67742 | | 985 | 5.478919 |
| 10 | 12805 | GO:0030246 | carbohydrate binding | 3.26E-02 | 0.633889 | 3 | 9.67742 | | 408 | 2.26944 |
| 11 | 1169 | GO:0001871 | pattern binding | 0.039115 | 0.709765 | 2 | 6.451613 | | 182 | 1.012348 |
| 12 | 2627 | GO:0003676 | nucleic acid binding | 0.985124 | 1 | 2 | 6.451613 | | 3296 | 18.33352 |
| 13 | 3475 | GO:0004871| GO:0005062| GO:0009369| GO:0009370 | signal transducer activity | 0.883412 | 1 | 2 | 6.451613 | | 2051 | 11.40839 |
| 14 | 11210 | GO:0019207 | kinase regulator activity | 0.018393 | 0.439256 | 2 | 6.451613 | | 121 | 0.673045 |
| 15 | 11787 | GO:0019842 | vitamin binding | 0.02289 | 0.501249 | 2 | 6.451613 | | 136 | 0.75648 |
| 16 | 19764 | GO:0043498 | cell surface binding | 0.003754 | 0.155072 | 2 | 6.451613 | | 53 | 0.294805 |
| 17 | 30178 | GO:0071814 | protein-lipid complex binding | 0.001297 | 0.071436 | 2 | 6.451613 | | 31 | 0.172433 |
| **B. Cellular Component** | | | | | | | | | | |
| 1 | 20657 | GO:0044464 | cell part | 0.999712 | 1 | 20 | 64.51613 | | 15644 | 87.01746 |
| 2 | 3940 | GO:0005615 | extracellular space | 1.94E-15 | 1.06E-11 | 17 | 54.83871 | | 819 | 4.555568 |
| 3 | 20615 | GO:0044421 | extracellular region part | 1.82E-13 | 6.62E-10 | 17 | 54.83871 | | 1080 | 6.007342 |
| 4 | 3945 | GO:0005622 | intracellular | 0.997179 | 1 | 14 | 45.16129 | | 12155 | 67.61041 |
| 5 | 20618 | GO:0044424 | intracellular part | 0.994839 | 1 | 14 | 45.16129 | | 11860 | 65.96952 |
| 6 | 9266 | GO:0016020 | membrane | 0.753968 | 1 | 12 | 38.70968 | | 7776 | 43.25287 |
| 7 | 19528 | GO:0043229 | intracellular organelle | 0.998386 | 1 | 10 | 32.25806 | | 10219 | 56.84169 |
| 8 | 19526 | GO:0043227 | membrane-bounded organelle | 0.996374 | 1 | 9 | 29.03226 | | 9200 | 51.17366 |
| 9 | 20616 | GO:0044422 | organelle part | 0.833645 | 1 | 9 | 29.03226 | | 6429 | 35.76037 |
| 10 | 20640 | GO:0044446 | intracellular organelle part | 0.821383 | 1 | 9 | 29.03226 | | 6354 | 35.3432 |
| 11 | 30306 | GO:0071944 | cell periphery | 0.343088 | 1 | 9 | 29.03226 | | 4410 | 24.52998 |
| 12 | 19533 | GO:0043234 | protein complex | 0.149372 | 1 | 8 | 25.80645 | | 3086 | 17.16542 |
| 12 | 13471 | GO:0031012 | extracellular matrix | 6.85E-05 | 0.006388 | 6 | 19.35484 | | 418 | 2.325064 |
| 14 | 14393 | GO:0031982 | vesicle | 0.003381 | 0.14453 | 6 | 19.35484 | | 875 | 4.86706 |
| 15 | 15395 | GO:0032994 | protein-lipid complex | 2.95E-11 | 5.37E-08 | 6 | 19.35484 | | 36 | 0.200245 |
| 16 | 16739 | GO:0034358 | plasma lipoprotein particle | 2.95E-11 | 5.37E-08 | 6 | 19.35484 | | 36 | 0.200245 |
| 17 | 20619 | GO:0044425 | membrane part | 0.984516 | 1 | 6 | 19.35484 | | 6378 | 35.47669 |
| 18 | 19532 | GO:0043233 | organelle lumen | 0.64198 | 1 | 5 | 16.12903 | | 3110 | 17.29892 |
| 19 | 202 | GO:0000267 | cell fraction | 1.42E-01 | 1.00E+00 | 4 | 12.90323 | | 1178 | 6.552453 |
| 20 | 19527 | GO:0043228 | non-membrane-bounded organelle | 0.898681 | 1 | 4 | 12.90323 | | 3637 | 20.23028 |
| 21 | 13547 | GO:0031090 | organelle membrane | 0.746795 | 1 | 3 | 9.67742 | | 2192 | 12.19268 |
| 22 | 19312 | GO:0042995 | cell projection | 0.268132 | 1 | 3 | 9.67742 | | 1045 | 5.81266 |
| 23 | 7228 | GO:0009986| GO:0009928| GO:0009929 | cell surface | 0.180225 | 1 | 2 | 6.451613 | | 448 | 2.491935 |
| 24 | 8211 | GO:0012505 | endomembrane system | 0.78684 | 1 | 2 | 6.451613 | | 1635 | 9.094449 |
| 25 | 20546 | GO:0044297 | cell body | 0.074146 | 1 | 2 | 6.451613 | | 261 | 1.451774 |
| 26 | 20614 | GO:0044420 | extracellular matrix part | 0.037569 | 0.692066 | 2 | 6.451613 | | 178 | 0.990099 |
| **C. Biological Process** | | | | | | | | | | |
| 1 | 25094 | GO:0050789| GO:0050791 | regulation of biological process | 0.002081 | 0.100401 | 24 | 77.41936 | | 9108 | 50.66192 |
| 2 | 5018 | GO:0006950 | response to stress | 2.09E-08 | 1.42E-05 | 18 | 58.06452 | | 2588 | 14.39537 |
| 3 | 20488 | GO:0044238 | primary metabolic process | 0.195791 | 1 | 15 | 48.3871 | | 7064 | 39.29247 |
| 4 | 28365 | GO:0065008 | regulation of biological quality | 1.28E-06 | 3.67E-04 | 15 | 48.3871 | | 2268 | 12.61542 |
| 5 | 19473 | GO:0043170| GO:0043283 | macromolecule metabolic process | 0.057281 | 0.904008 | 14 | 45.16129 | | 5445 | 30.28702 |
| 6 | 25998 | GO:0051716 | cellular response to stimulus | 0.038377 | 0.705755 | 14 | 45.16129 | | 5175 | 28.78518 |
| 7 | 28366 | GO:0065009 | regulation of molecular function | 9.30E-06 | 0.001398 | 14 | 45.16129 | | 2296 | 12.77117 |
| 8 | 4909 | GO:0006810| GO:0015457| GO:0015460 | transport | 0.003878 | 0.158986 | 12 | 38.70968 | | 3113 | 17.31561 |
| 9 | 5022 | GO:0006955 | immune response | 6.05E-09 | 5.81E-06 | 12 | 38.70968 | | 838 | 4.661253 |
| 10 | 18582 | GO:0042221 | response to chemical stimulus | 0.002656 | 0.124333 | 12 | 38.70968 | | 2979 | 16.57025 |
| 11 | 25529 | GO:0051234 | establishment of localization | 0.004427 | 0.176199 | 12 | 38.70968 | | 3162 | 17.58816 |
| 12 | 20487 | GO:0044237 | cellular metabolic process | 0.829178 | 1 | 10 | 32.25806 | | 7008 | 38.98098 |
| 13 | 9285 | GO:0016043 | cellular component organization | 0.13654 | 1 | 9 | 29.03226 | | 3520 | 19.57949 |
| 14 | 5280 | GO:0007275 | multicellular organismal development | 0.247732 | 1 | 8 | 25.80645 | | 3511 | 19.52943 |
| 15 | 24235 | GO:0048856 | anatomical structure development | 0.187943 | 1 | 8 | 25.80645 | | 3265 | 18.16109 |
| 16 | 5195 | GO:0007165| GO:0023033 | signal transduction | 0.642255 | 1 | 7 | 22.58065 | | 4317 | 24.01268 |
| 17 | 9409 | GO:0016192| GO:0006899 | vesicle-mediated transport | 3.02E-04 | 0.021106 | 7 | 22.58065 | | 782 | 4.349761 |
| 18 | 20531 | GO:0044281 | small molecule metabolic process | 0.144519 | 1 | 7 | 22.58065 | | 2571 | 14.30081 |
| 19 | 25925 | GO:0051641 | cellular localization | 0.009256 | 0.285155 | 7 | 22.58065 | | 1421 | 7.904105 |
| 20 | 1073 | GO:0001775 | cell activation | 1.83E-04 | 0.013622 | 6 | 19.35484 | | 500 | 2.781177 |
| 21 | 1527 | GO:0002252 | immune effector process | 1.52E-06 | 3.86E-04 | 6 | 19.35484 | | 214 | 1.190344 |
| 22 | 1528 | GO:0002253 | activation of immune response | 4.91E-06 | 9.59E-04 | 6 | 19.35484 | | 262 | 1.457337 |
| 23 | 2674 | GO:0003824 | catalytic activity | 0.935438 | 1 | 6 | 19.35484 | | 5368 | 29.85872 |
| 24 | 11695 | GO:0019725 | cellular homeostasis | 3.88E-04 | 0.026785 | 6 | 19.35484 | | 575 | 3.198354 |
| 25 | 15436 | GO:0033036 | macromolecule localization | 0.035818 | 0.673458 | 6 | 19.35484 | | 1458 | 8.109912 |
| 26 | 25120 | GO:0050817 | coagulation | 1.40E-04 | 0.011564 | 6 | 19.35484 | | 476 | 2.647681 |
| 27 | 25178 | GO:0050878 | regulation of body fluid levels | 3.57E-04 | 0.024779 | 6 | 19.35484 | | 566 | 3.148292 |
| 28 | 25933 | GO:0051649 | establishment of localization in cell | 0.017148 | 0.4193 | 6 | 19.35484 | | 1232 | 6.85282 |
| 29 | 30735 | GO:0072376 | protein activation cascade | 4.32E-09 | 4.71E-06 | 6 | 19.35484 | | 80 | 0.444988 |
| 30 | 4906 | GO:0006807 | nitrogen compound metabolic process | 0.826805 | 1 | 5 | 16.12903 | | 3862 | 21.48181 |
| 31 | 6921 | GO:0009653 | anatomical structure morphogenesis | 0.14252 | 1 | 5 | 16.12903 | | 1622 | 9.022139 |
| 32 | 6985 | GO:0009719 | response to endogenous stimulus | 0.01188 | 0.329911 | 5 | 16.12903 | | 812 | 4.516632 |
| 33 | 15341 | GO:0032940 | secretion by cell | 2.42E-04 | 0.017245 | 5 | 16.12903 | | 333 | 1.852264 |
| 34 | 24247 | GO:0048869 | cellular developmental process | 0.35326 | 1 | 5 | 16.12903 | | 2271 | 12.63211 |
| 35 | 30205 | GO:0071841 | cellular component organization or biogenesis at cellular level | 0.56102 | 1 | 5 | 16.12903 | | 2857 | 15.89165 |
| 36 | 6425 | GO:0009056 | catabolic process | 0.408404 | 1 | 4 | 12.90323 | | 1877 | 10.44054 |
| 37 | 6882 | GO:0009605 | response to external stimulus | 0.102414 | 1 | 4 | 12.90323 | | 1043 | 5.801535 |
| 38 | 20336 | GO:0044085 | cellular component biogenesis | 0.172151 | 1 | 4 | 12.90323 | | 1269 | 7.058627 |
| 39 | 26931 | GO:0055085 | transmembrane transport | 0.11559 | 1 | 4 | 12.90323 | | 1090 | 6.062966 |
| 40 | 2208 | GO:0003008 | system process | 0.626052 | 1 | 3 | 9.67742 | | 1835 | 10.20692 |
| 41 | 5544 | GO:0007586 | digestion | 5.42E-04 | 0.034587 | 3 | 9.67742 | | 93 | 0.517299 |
| 42 | 6427 | GO:0009058 | biosynthetic process | 0.898413 | 1 | 3 | 9.67742 | | 2912 | 16.19758 |
| 43 | 6884 | GO:0009607 | response to biotic stimulus | 0.092033 | 1 | 3 | 9.67742 | | 626 | 3.482034 |
| 44 | 11616 | GO:0019637 | organophosphate metabolic process | 0.009157 | 0.28289 | 3 | 9.67742 | | 252 | 1.401713 |
| 45 | 25530 | GO:0051235 | maintenance of location | 9.08E-04 | 0.053226 | 3 | 9.67742 | | 111 | 0.617421 |
| 46 | 25935 | GO:0051651 | maintenance of location in cell | 5.25E-04 | 0.033708 | 3 | 9.67742 | | 92 | 0.511737 |
| 47 | 25989 | GO:0051707| GO:0009613| GO:0042828 | response to other organism | 0.050201 | 0.838187 | 3 | 9.67742 | | 485 | 2.697742 |
| 48 | 30191 | GO:0071827 | plasma lipoprotein particle organization | 1.32E-05 | 0.001756 | 3 | 9.67742 | | 27 | 0.150184 |
| 49 | 2206 | GO:0003006 | developmental process involved in reproduction | 0.139608 | 1 | 2 | 6.451613 | | 381 | 2.119257 |
| 50 | 5001 | GO:0006928 | cellular component movement | 0.304889 | 1 | 2 | 6.451613 | | 643 | 3.576594 |
| 51 | 5184 | GO:0007154 | cell communication | 0.590619 | 1 | 2 | 6.451613 | | 1134 | 6.307709 |
| 52 | 5185 | GO:0007155 | cell adhesion | 0.373058 | 1 | 2 | 6.451613 | | 750 | 4.171765 |
| 53 | 5756 | GO:0008219 | cell death | 0.453027 | 1 | 2 | 6.451613 | | 881 | 4.900434 |
| 54 | 9286 | GO:0016044 | cellular membrane organization | 0.221191 | 1 | 2 | 6.451613 | | 513 | 2.853488 |
| 55 | 12481 | GO:0022414 | reproductive process | 0.534936 | 1 | 2 | 6.451613 | | 1026 | 5.706975 |
| 56 | 16762 | GO:0034381 | plasma lipoprotein particle clearance | 5.92E-04 | 0.036686 | 2 | 6.451613 | | 21 | 0.116809 |
| 57 | 18791 | GO:0042445 | hormone metabolic process | 0.02289 | 0.501249 | 2 | 6.451613 | | 136 | 0.75648 |
| 58 | 20613 | GO:0044419 | interspecies interaction between organisms | 0.130254 | 1 | 2 | 6.451613 | | 365 | 2.030259 |
| 59 | 24248 | GO:0048870 | cell motility | 0.251343 | 1 | 2 | 6.451613 | | 560 | 3.114918 |
| 60 | 24249 | GO:0048871 | multicellular organismal homeostasis | 0.013817 | 0.360488 | 2 | 6.451613 | | 104 | 0.578485 |
| 61 | 25957 | GO:0051674 | localization of cell | 0.251343 | 1 | 2 | 6.451613 | | 560 | 3.114918 |

# Second level of GO terms that were enriched in two or more proteins depicted.

**^*^** Proteins identified in *falciparum* malaria that has the term, **^•^** Percentage of proteins identified in *falciparum* malaria that has the term, **^** Number of proteins in Biological Genome for Human that has the term, ^$^ Percentage of proteins in Biological Genome for Human that has the term
