# Supplementary material for: Proteomic Investigation of Falciparum and Vivax Malaria for Identification of Surrogate Protein Markers
Source: PLoS One. 2012 Aug 9;7(8):e41751. doi: 10.1371/journal.pone.0041751 (PMC3415403; doi:10.1371/journal.pone.0041751)
Supplement: Table S7 — Summary of different physiological pathways associated with the differentially expressed proteins identified in falciparum and vivax malaria. (DOC) [file pone.0041751.s016.doc]

**Table S7**. Summary of different physiological pathways associated with the differentially expressed proteins identified in *falciparum* and *vivax* malaria using multiple software*

1. *Falciparum* malaria

| **Sl No.** | **Pathways** | **IPA** | **DAVID** | **PANTHER** | **GeneSpring** |
| --- | --- | --- | --- | --- | --- |
| 1 | Coagulation cascades | 1 | 1 | 1 | 1 |
| 2 | Complement cascades | 1 | 1 | 0 | 3 |
| 3 | Lipid and Lipoprotein metabolism/transport | 2 | 0 | 0 | 8 |
| 4 | G-protein- signaling | 2 | 0 | 2 | 1 |
| 5 | APR Signaling and inflammation | 1 | 0 | 1 | 2 |
| 6 | Immune signaling | 3 | 0 | 1 | 2 |
| 7 | Transcription activation | 0 | 0 | 0 | 3 |
| 8 | p73 transcription factor network | 0 | 0 | 0 | 1 |
| 9 | Transport of vitamins, nucleosides, and related molecules | 0 | 0 | 0 | 2 |
| 10 | Cell surface interactions | 0 | 0 | 0 | 5 |

1. *Vivax* malaria

| **Sl No.** | **Pathways** | **IPA** | **DAVID** | **PANTHER** | **GeneSpring** |
| --- | --- | --- | --- | --- | --- |
| 1 | Coagulation cascades | 1 | 2 | 1 | 1 |
| 2 | Complement cascades | 1 | 1 | 0 | 1 |
| 3 | Lipid and Lipoprotein metabolism/transport | 2 | 0 | 0 | 7 |
| 4 | APR Signaling and inflammation | 1 | 0 | 1 | 1 |
| 5 | Immune signaling | 3 | 0 | 1 | 2 |
| 6 | Vitamin D metabolism and pathway | 0 | 0 | 1 | 1 |
| 7 | IL-6 and IL-10 signaling | 2 | 0 | 1 | 2 |
| 8 | Transport of vitamins, nucleosides, and related molecules | 0 | 0 | 0 | 2 |
| 9 | p73 transcription factor network | 0 | 0 | 0 | 1 |
| 10 | Cell surface interactions | 0 | 0 | 0 | 6 |
| 11 | Transcription activation | 0 | 0 | 0 | 4 |

***** Scores indicates the number of similar types of pathways identified by particular software

IPA-: Ingenuity Pathway Analysis (*version* 9.0)

DAVID-: Database for Annotation, Visualization and Integrated Discovery (*version* 6.7)

PANTHER-: Protein ANalysis THrough Evolutionary Relationships (*version* 7)

GeneSpring Software (*version* 11.5)
