# Supplementary material for: Proteomic Investigation of Falciparum and Vivax Malaria for Identification of Surrogate Protein Markers
Source: PLoS One. 2012 Aug 9;7(8):e41751. doi: 10.1371/journal.pone.0041751 (PMC3415403; doi:10.1371/journal.pone.0041751)
Supplement: Table S8 — Prediction models for discrimination of malaria (FM and VM) and controls (HC and FC). (DOC) [file pone.0041751.s017.doc]

**Table S8.** Prediction models for discrimination of malaria (FM and VM) and controls (HC and FC)

**Table S8. 1A.** Selected proteins from 2DE data used to build prediction model for discrimination of FM and HC

| **Sl No.** | UniProt **accession number** | **Name of the proteins** |
| --- | --- | --- |
| 1 | P00738 | Haptoglobin (HP) |
| 2 | P02647 | Apolipoprotein A-I (Apo-AI) |
| 3 | P02790 | Hemopexin (Beta-1B-glycoprotein) |
| 4 | P02647 | Apolipoprotein E (Apo-E) |
| 5 | P02735 | Serum amyloid A (SAA) |

**Table S8.1B.** Prediction summary of various classification methods for discrimination of FM and HC based on the 5 proteins identified using 2DE

| **PLSDA** |  |  | **FM** | **HC** | **Accuracy** |
| --- | --- | --- | --- | --- | --- |
|  | Model Training | FM | 10 | 0 | 100% |
|  |  | HC | 0 | 10 | 100% |
|  | Cross Validation | FM | 10 | 0 | 100% |
|  |  | HC | 0 | 10 | 100% |
|  | Prediction | FM | 19 | 0 | 100% |
|  |  | HC | 1 | 18 | 94.74% |
| **Decision Tree** | Model Training | FM | 10 | 0 | 100% |
|  |  | HC | 0 | 10 | 100% |
|  | Cross Validation | FM | 10 | 0 | 100% |
|  |  | HC | 0 | 10 | 100% |
|  | Prediction | FM | 19 | 0 | 100% |
|  |  | HC | 1 | 18 | 94.74% |
| **SVM** | Model Training | FM | 10 | 0 | 100% |
|  |  | HC | 0 | 10 | 100% |
|  | Cross Validation | FM | 9 | 1 | 90% |
|  |  | HC | 1 | 9 | 90% |
|  | Prediction | FM | 19 | 0 | 100% |
|  |  | HC | 1 | 18 | 94.74% |
| **Naïve Bayes** | Model Training | FM | 9 | 1 | 90% |
|  |  | HC | 0 | 10 | 100% |
|  | Cross Validation | FM | 8 | 2 | 80% |
|  |  | HC | 0 | 10 | 100% |
|  | Prediction | FM | 19 | 0 | 100% |
|  |  | HC | 1 | 18 | 94.74% |

**Table S8.1C.** Strength of the prediction (confidence measure) associated with independent blinded prediction performed on FM and HC samples using model created with 5 proteins identified in 2DE

|  | **PLS-DA** | | **Decision Tree** | | **SVM** | | **Naïve Bayes** | |
| --- | --- | --- | --- | --- | --- | --- | --- | --- |
| **Sample ID** | **Predicted** | **Confidence Measure** | **Predicted** | **Confidence Measure** | **Predicted** | **Confidence Measure** | **Predicted** | **Confidence Measure** |
| HC03 | [HC] | 1 | [HC] | 1 | [HC] | 0.440209 | [HC] | 0.999768 |
| HC08 | [HC] | 1 | [HC] | 1 | [HC] | 0.418606 | [HC] | 0.999727 |
| HC09 | [HC] | 1 | [HC] | 1 | [HC] | 0.371226 | [HC] | 0.999738 |
| HC11 | [HC] | 1 | [HC] | 1 | [HC] | 0.349611 | [HC] | 0.999691 |
| HC12 | [HC] | 1 | [HC] | 1 | [HC] | 0.418606 | [HC] | 0.999727 |
| HC13 | [HC] | 1 | [HC] | 1 | [HC] | 0.630753 | [HC] | 0.999898 |
| HC14 | [HC] | 1 | [HC] | 1 | [HC] | 0.271677 | [HC] | 0.999607 |
| HC15 | [HC] | 1 | [HC] | 1 | [HC] | 0.652368 | [HC] | 0.999913 |
| HC20 | [HC] | 1 | [HC] | 1 | [HC] | 0.315866 | [HC] | 0.998353 |
| HC21 | [HC] | 1 | [HC] | 1 | [HC] | 0.315867 | [HC] | 0.999825 |
| HC22 | [HC] | 1 | [HC] | 1 | [HC] | 0.316655 | [HC] | 0.95901 |
| HC29 | [HC] | 1 | [HC] | 1 | [HC] | 0.794179 | [HC] | 1 |
| HC30 | [HC] | 1 | [HC] | 1 | [HC] | 0.369796 | [HC] | 0.949882 |
| HC31 | [HC] | 1 | [HC] | 1 | [HC] | 0.401255 | [HC] | 0.999999 |
| HC32 | [HC] | 1 | [HC] | 1 | [HC] | 0.794179 | [HC] | 1 |
| HC33 | [HC] | 1 | [HC] | 1 | [HC] | 0.794179 | [HC] | 1 |
| HC34 | [HC] | 1 | [HC] | 1 | [HC] | 0.994728 | [HC] | 1 |
| HC37 | [HC] | 1 | [HC] | 1 | [HC] | 1 | [HC] | 1 |
| HC38 | [FM]* | 1 | [FM]* | 1 | [FM]* | 1 | [FM]* | 0.545221 |
| P02 | [FM] | 1 | [FM] | 1 | [FM] | 0.10191 | [FM] | 0.551057 |
| P03 | [FM] | 1 | [FM] | 1 | [FM] | 0.772445 | [FM] | 0.920782 |
| P04 | [FM] | 1 | [FM] | 1 | [FM] | 0.643653 | [FM] | 0.999993 |
| P05 | [FM] | 1 | [FM] | 1 | [FM] | 0.520787 | [FM] | 0.984178 |
| P22 | [FM] | 1 | [FM] | 1 | [FM] | 0.780166 | [FM] | 0.99999 |
| P26 | [FM] | 1 | [FM] | 1 | [FM] | 0.52571 | [FM] | 0.79283 |
| P27 | [FM] | 1 | [FM] | 1 | [FM] | 0.595207 | [FM] | 0.994234 |
| P28 | [FM] | 1 | [FM] | 1 | [FM] | 1 | [FM] | 0.999949 |
| P29 | [FM] | 1 | [FM] | 1 | [FM] | 0.528143 | [FM] | 0.998964 |
| P30 | [FM] | 1 | [FM] | 1 | [FM] | 1 | [FM] | 0.999909 |
| P31 | [FM] | 1 | [FM] | 1 | [FM] | 0.772445 | [FM] | 0.920782 |
| P32 | [FM] | 1 | [FM] | 1 | [FM] | 1 | [FM] | 1 |
| P33 | [FM] | 1 | [FM] | 1 | [FM] | 0.348522 | [FM] | 0.770211 |
| P34 | [FM] | 1 | [FM] | 1 | [FM] | 0.3818 | [FM] | 0.99401 |
| P35 | [FM] | 1 | [FM] | 1 | [FM] | 0.849295 | [FM] | 1 |
| P36 | [FM] | 1 | [FM] | 1 | [FM] | 0.563137 | [FM] | 0.998237 |
| P37 | [FM] | 1 | [FM] | 1 | [FM] | 1 | [FM] | 1 |
| P38 | [FM] | 1 | [FM] | 1 | [FM] | 0.689535 | [FM] | 0.99993 |
| P39 | [FM] | 1 | [FM] | 1 | [FM] | 0.348875 | [FM] | 0.995111 |

* Improper prediction

**Table S8.2A.** Selected proteins from 2D-DIGE data used to build prediction models for discrimination of FM, VM and HC

| **Sl No.** | UniProt **accession number** | **Name of the proteins** |
| --- | --- | --- |
| 1 | P00738 | Haptoglobin (HP) |
| 2 | P02647 | Apolipoprotein A-I (Apo-AI) |
| 3 | P02790 | Hemopexin (Beta-1B-glycoprotein) |
| 4 | P02647 | Apolipoprotein E (Apo-E) |
| 5 | P02735 | Serum amyloid A (SAA) |
| 6 | P02753 | Retinol-binding protein (RBP) |
| 7 | P02774 | Vitamin D-binding protein (DBP) |

**Table S8. 2B.** Prediction summary of various classification methods for discrimination of FM, VM and HC based on the 7 proteins identified using 2D-DIGE

| **PLSDA** |  |  | **FM** | **VM** | **HC** | **Accuracy** |
| --- | --- | --- | --- | --- | --- | --- |
|  | Model Training | FM | 6 | 0 | 0 | 100.00% |
|  |  | VM | 0 | 5 | 0 | 100.00% |
|  |  | HC | 0 | 0 | 5 | 100.00% |
|  | Cross Validation | FM | 6 | 0 | 0 | 100.00% |
|  |  | VM | 0 | 5 | 0 | 100.00% |
|  |  | HC | 0 | 0 | 5 | 100.00% |
|  | Prediction | FM | 8 | 0 | 0 | 100.00% |
|  |  | VM | 0 | 7 | 0 | 100.00% |
|  |  | HC | 0 | 1 | 7 | 87.50% |
| **Decision Tree** | Model Training | FM | 6 | 0 | 0 | 100.00% |
|  |  | VM | 0 | 5 | 0 | 100.00% |
|  |  | HC | 0 | 0 | 5 | 100.00% |
|  | Cross Validation | FM | 6 | 0 | 0 | 100.00% |
|  |  | VM | 1 | 4 | 0 | 80.00% |
|  |  | HC | 1 | 0 | 4 | 80.00% |
|  | Prediction | FM | 8 | 0 | 0 | 100.00% |
|  |  | VM | 0 | 7 | 0 | 100.00% |
|  |  | HC | 0 | 0 | 8 | 100.00% |
| **SVM** | Model Training | FM | 6 | 0 | 0 | 100.00% |
|  |  | VM | 0 | 5 | 0 | 100.00% |
|  |  | HC | 0 | 0 | 5 | 100.00% |
|  | Cross Validation | FM | 6 | 0 | 0 | 100.00% |
|  |  | VM | 0 | 5 | 0 | 100.00% |
|  |  | HC | 0 | 0 | 5 | 100.00% |
|  | Prediction | FM | 8 | 0 | 0 | 100.00% |
|  |  | VM | 0 | 7 | 0 | 100.00% |
|  |  | HC | 0 | 1 | 7 | 87.50% |
| **Naïve Bayes** | Model Training | FM | 6 | 0 | 0 | 100.00% |
|  |  | VM | 0 | 5 | 0 | 100.00% |
|  |  | HC | 0 | 0 | 5 | 100.00% |
|  | Cross Validation | FM | 5 | 0 | 1 | 83.33% |
|  |  | VM | 1 | 4 | 0 | 80.00% |
|  |  | HC | 1 | 0 | 4 | 80.00% |
|  | Prediction | FM | 6 | 1 | 1 | 75.00% |
|  |  | VM | 0 | 7 | 0 | 100.00% |
|  |  | HC | 0 | 0 | 8 | 100.00% |

**Table S8.2C. Strength of the prediction (confidence measure) associated with independent blinded prediction performed on FM, VM and HC samples using model created with 7 proteins identified in DIGE**

|  | **PLS-DA** | | **Decision Trees** | | **SVM** | | **Naïve Bayes** | |
| --- | --- | --- | --- | --- | --- | --- | --- | --- |
| **Sample ID** | **Predicted** | **Confidence measure** | **Predicted** | **Confidence Measure** | **Predicted** | **Confidence Measure** | **Predicted** | **Confidence Measure** |
| HC1 | [HC] | 0.8692318 | [HC] | 1 | [HC] | 1 | [HC] | 1 |
| HC2 | [HC] | 0.9741194 | [HC] | 1 | [HC] | 0.877528 | [HC] | 0.999964 |
| HC3 | [HC] | 0.8091353 | [HC] | 1 | [HC] | 0.877589 | [HC] | 1 |
| HC4 | [HC] | 0.8484311 | [HC] | 1 | [HC] | 0.971986 | [HC] | 0.999978 |
| HC5 | [VM]* | 0.914948 | [HC] | 1 | [VM]* | 0.84228 | [HC] | 1 |
| HC6 | [HC] | 0.8402068 | [HC] | 1 | [HC] | 0.922744 | [HC] | 0.999994 |
| HC7 | [HC] | 0.7624396 | [HC] | 1 | [HC] | 0.791335 | [HC] | 0.999999 |
| HC8 | [HC] | 0.80341333 | [HC] | 1 | [HC] | 0.674115 | [HC] | 0.998998 |
| FM1 | [FM] | 0.9471071 | [FM] | 1 | [FM] | 0.885737 | [FM] | 0.999991 |
| FM2 | [FM] | 0.8381384 | [FM] | 1 | [FM] | 0.898764 | [FM] | 0.999996 |
| FM3 | [FM] | 0.8929898 | [FM] | 1 | [FM] | 0.896551 | [FM] | 0.99998 |
| FM4 | [FM] | 0.8901021 | [FM] | 1 | [FM] | 0.90205 | [FM] | 0.999998 |
| FM5 | [FM] | 0.7987965 | [FM] | 1 | [FM] | 0.935461 | [FM] | 1 |
| FM6 | [FM] | 0.78664726 | [FM] | 1 | [FM] | 0.877527 | [FM] | 0.999462 |
| FM7 | [FM] | 0.80169743 | [FM] | 1 | [FM] | 0.928133 | [HC]* | 0.99905 |
| FM8 | [FM] | 0.7587608 | [FM] | 1 | [FM] | 0.955072 | [VM]* | 1 |
| VM1 | [VM] | 0.831925 | [VM] | 1 | [VM] | 0.883102 | [VM] | 1 |
| VM2 | [VM] | 0.8603673 | [VM] | 1 | [VM] | 0.877573 | [VM] | 1 |
| VM4 | [VM] | 0.84051543 | [VM] | 1 | [VM] | 0.935545 | [VM] | 0.999999 |
| VM5 | [VM] | 0.8802684 | [VM] | 1 | [VM] | 0.880737 | [VM] | 1 |
| VM6 | [VM] | 0.97583246 | [VM] | 1 | [VM] | 0.877562 | [VM] | 0.999999 |
| VM7 | [VM] | 0.9225021 | [VM] | 1 | [VM] | 0.877556 | [VM] | 0.999556 |
| VM8 | [VM] | 0.85696614 | [VM] | 1 | [VM] | 0.916288 | [VM] | 1 |

* Improper prediction

**Table S8. 3A.** Selected proteins from DIGE data used to build prediction models for discrimination of FM, VM and FC (Leptospirosis)

| **Sl No.** | UniProt **accession number** | **Name of the proteins** |
| --- | --- | --- |
| 1 | P02735 | Serum amyloid A (SAA) |
| 3 | P02790 | Hemopexin (Beta-1B-glycoprotein) |
| 3 | P02647 | Apolipoprotein E (Apo-E) |
| 4 | P00738 | Haptoglobin (HP) |
| 5 | P02753 | Retinol-binding protein (RBP) |
| 6 | P02647 | Apolipoprotein A-I (Apo-AI) |

**Table S8. 3B.** Prediction summary for various classification methods for discrimination of FM, VM and FC (Leptospirosis) based on the 6 proteins identified using 2D-DIGE

| **Decision Tree** |  |  | **FM** | **Lep** | **VM** | **Accuracy** |
| --- | --- | --- | --- | --- | --- | --- |
|  | Model Training | FM | 7 | 0 | 0 | 100.00% |
|  |  | Lep | 0 | 6 | 0 | 100.00% |
|  |  | VM | 0 | 0 | 7 | 100.00% |
|  | Cross Validation | FM | 7 | 0 | 0 | 100.00% |
|  |  | Lep | 0 | 6 | 0 | 100.00% |
|  |  | VM | 0 | 0 | 7 | 100.00% |
|  | Prediction | FM | 8 | 0 | 0 | 100.00% |
|  |  | Lep | 0 | 6 | 0 | 100.00% |
|  |  | VM | 0 | 0 | 8 | 100.00% |
| **Naïve Bayes** | Model Training | FM | 7 | 0 | 0 | 100.00% |
|  |  | Lep | 0 | 6 | 0 | 100.00% |
|  |  | VM | 1 | 0 | 6 | 85.71% |
|  | Cross Validation | FM | 4 | 0 | 3 | 57.14% |
|  |  | Lep | 0 | 6 | 0 | 100.00% |
|  |  | VM | 2 | 0 | 5 | 71.42% |
|  | Prediction | FM | 7 | 0 | 1 | 87.50% |
|  |  | Lep | 0 | 6 | 0 | 100.00% |
|  |  | VM | 0 | 0 | 8 | 100.00% |
| **PLSDA** | Model Training | FM | 6 | 1 | 1 | 71.42% |
|  |  | Lep | 0 | 6 | 0 | 100.00% |
|  |  | VM | 0 | 0 | 7 | 100.00% |
|  | Cross Validation | FM | 4 | 2 | 1 | 57.14% |
|  |  | Lep | 0 | 6 | 0 | 100.00% |
|  |  | VM | 1 | 0 | 6 | 85.71% |
|  | Prediction | FM | 7 | 0 | 1 | 87.50% |
|  |  | Lep | 0 | 6 | 0 | 100.00% |
|  |  | VM | 1 | 0 | 7 | 87.50% |
| **SVM** | Model Training | FM | 7 | 0 | 0 | 100.00% |
|  |  | Lep | 0 | 6 | 0 | 100.00% |
|  |  | VM | 0 | 0 | 7 | 100.00% |
|  | Cross Validation | FM | 6 | 1 | 0 | 85.71% |
|  |  | Lep | 0 | 6 | 0 | 100.00% |
|  |  | VM | 2 | 1 | 4 | 57.14% |
|  | Prediction | FM | 8 | 0 | 0 | 100.00% |
|  |  | Lep | 0 | 6 | 0 | 100.00% |
|  |  | VM | 1 | 0 | 7 | 87.50% |

**Table S8. 3C. Strength of the prediction (confidence measure) associated with independent blinded prediction performed on FM, VM and FC (Leptospirosis) samples using model created with 6 proteins identified in DIGE**

|  |  | **Decision Tree** | | **Naive Bayes** | | **PLSDA** | | **SVM** | |
| --- | --- | --- | --- | --- | --- | --- | --- | --- | --- |
| **Sample** | **Actual Group** | **Predicted Group** | **Confidence Measure** | **Predicted Group** | **Confidence Measure** | **Predicted Group** | **Confidence Measure** | **Predicted Group** | **Confidence Measure** |
| FM1 | [FM] | [FM] | 1 | [FM] | 0.9910882 | [FM] | 0.6626713 | [FM] | 0.1964062 |
| FM2 | [FM] | [FM] | 1 | [FM] | 0.7983825 | [FM] | 0.5160511 | [FM] | 0.1650509 |
| FM3 | [FM] | [FM] | 1 | [FM] | 0.6823516 | [FM] | 0.9869202 | [FM] | 0.1242963 |
| FM4 | [FM] | [FM] | 1 | [FM] | 0.9637253 | [FM] | 0.8307237 | [FM] | 0.9973446 |
| FM5 | [FM] | [FM] | 1 | [VM]* | 0.5345048 | [FM] | 0.5524298 | [FM] | 1 |
| FM6 | [FM] | [FM] | 1 | [FM] | 0.8924397 | [VM]* | 0.704438 | [FM] | 0.0969757 |
| FM7 | [FM] | [FM] | 1 | [FM] | 0.992229 | [FM] | 0.5104709 | [FM] | 0.0861475 |
| FM8 | [FM] | [FM] | 1 | [FM] | 0.9976184 | [FM] | 0.8332528 | [FM] | 0.6974816 |
| Lep1 | [Lep] | [Lep] | 1 | [Lep] | 0.9999408 | [Lep] | 0.7467884 | [Lep] | 0.0950166 |
| Lep2 | [Lep] | [Lep] | 1 | [Lep] | 0.9999809 | [Lep] | 0.9287416 | [Lep] | 0.0951259 |
| Lep3 | [Lep] | [Lep] | 1 | [Lep] | 0.9999906 | [Lep] | 0.8391516 | [Lep] | 0.1077943 |
| Lep4 | [Lep] | [Lep] | 1 | [Lep] | 0.9999763 | [Lep] | 0.9053305 | [Lep] | 0.0987363 |
| Lep5 | [Lep] | [Lep] | 1 | [Lep] | 0.999984 | [Lep] | 0.7322529 | [Lep] | 0.1211276 |
| Lep6 | [Lep] | [Lep] | 1 | [Lep] | 0.9999918 | [Lep] | 0.8662718 | [Lep] | 0.1060622 |
| VM1 | [VM] | [VM] | 1 | [VM] | 0.9961779 | [VM] | 0.9835747 | [VM] | 0.1823657 |
| VM2 | [VM] | [VM] | 1 | [VM] | 0.9726488 | [VM] | 0.6253945 | [VM] | 0.0831396 |
| VM3 | [VM] | [VM] | 1 | [VM] | 0.9998047 | [FM]* | 0.5396896 | [FM]* | 1 |
| VM4 | [VM] | [VM] | 1 | [VM] | 0.9579532 | [VM] | 0.9420067 | [VM] | 0.2549216 |
| VM5 | [VM] | [VM] | 1 | [VM] | 0.6971855 | [VM] | 0.6509802 | [VM] | 0.0894331 |
| VM6 | [VM] | [VM] | 1 | [VM] | 0.9999996 | [VM] | 0.668416 | [VM] | 0.3800836 |
| VM7 | [VM] | [VM] | 1 | [VM] | 0.9999994 | [VM] | 0.8630779 | [VM] | 0.3551832 |
| VM8 | [VM] | [VM] | 1 | [VM] | 0.9999991 | [VM] | 0.8990431 | [VM] | 0.5157065 |

* Improper prediction
