# Supplementary material for: Proteomic Investigation of Falciparum and Vivax Malaria for Identification of Surrogate Protein Markers
Source: PLoS One. 2012 Aug 9;7(8):e41751. doi: 10.1371/journal.pone.0041751 (PMC3415403; doi:10.1371/journal.pone.0041751)
Supplement: Table S9 — Receiver operating characteristic (ROC) curve for evaluating performance of different serum proteins on malaria prediction. (DOC) [file pone.0041751.s018.doc]

**Table S9.** Receiver operating characteristic (ROC) curve parameters showing performance of different serum proteins on malaria and leptospirosis (febrile control) prediction*

| **1. Apolipoprotein A1** | | | |
| --- | --- | --- | --- |
|  | ***Falciparum* Malaria** | ***Vivax* Malaria** | **Leptospirosis** |
| Area under the curve (AUC) | 0.9575 | 0.9794 | 0.7833 |
| Sensitivity | 95% | 94.12% | 66.6% |
| Specificity | 90% | 95% | 90% |
| Threshold value | 112.1 mg/dL | 96.59 mg/dL | 111.1 mg/dL |
| **2. Haptoglobin** | | | |
| Area under the curve (AUC) | 0.9363 | 0.8882 | 0.5083 |
| Sensitivity | 90% | 76.47% | 66.6% |
| Specificity | 95% | 95% | 50% |
| Threshold value | 0.465 g/L | 0.45 g/L | 0.845 g/L |
| **3. Retinol binding protein** | | | |
| Area under the curve (AUC) | 0.8792 | 0.875 | 0.5583 |
| Sensitivity | 66.6% | 75% | 50% |
| Specificity | 80% | 90% | 65% |
| Threshold value | 30.88μg/mL | 28.61 μg/mL | 34.99 μg/mL |

* ROC curve parameters for disease cases (malaria and leptospirosis) versus healthy controls
